# Membranes of Polymer of Intrinsic Microporosity PIM-1 for Gas Separation: Modification Strategies and Meta-Analysis

**DOI:** 10.1007/s40820-024-01610-2

**Published:** 2025-01-23

**Authors:** Boya Qiu, Yong Gao, Patricia Gorgojo, Xiaolei Fan

**Affiliations:** 1 Department of Chemical Engineering, Faculty of Science and Engineering, The University of Manchester, Manchester, M13 9PL UK; 2 Institute of Wenzhou, Zhejiang University, Wenzhou, 325006 People’s Republic of China; 3 Instituto de Nanociencia y Materiales de Aragón (INMA) CSIC-Universidad de Zaragoza, Mariano Esquillor, 50018 Zaragoza, Spain; 4 Departamento de Ingeniería Química y Tecnologías del Medio Ambiente, Universidad de Zaragoza, Pedro Cerbuna 12, 50009 Zaragoza, Spain; 5 Ningbo China Beacons of Excellence Research and Innovation Institute, University of Nottingham Ningbo China, 211 Xingguang Road, Ningbo, 315048 People’s Republic of China

**Keywords:** Polymers of intrinsic microporosity (PIMs), PIM-1, Gas separation, Meta-analysis, Upper bound

## Abstract

Polymers of intrinsic microporosity (PIMs) have received considerable attention for making high-performance membranes for carbon dioxide separation over the last two decades, owing to their highly permeable porous structures. However, challenges regarding its relatively low selectivity, physical aging, and plasticisation impede relevant industrial adoptions for gas separation. To address these issues, several strategies including chain modification, post-modification, blending with other polymers, and the addition of fillers, have been developed and explored. PIM-1 is the most investigated PIMs, and hence here we review the state-of-the-arts of the modification strategies of PIM-1 critically and discuss the progress achieved for addressing the aforementioned challenges via meta-analysis. Additionally, the development of PIM-1-based thin film composite membranes is commented as well, shedding light on their potential in industrial gas separation. We hope that the review can be a timely snapshot of the relevant state-of-the-arts of PIMs guiding future design and optimisation of PIMs-based membranes for enhanced performance towards a higher technology readiness level for practical applications.

**Supplementary Information:**

The online version contains supplementary material available at 10.1007/s40820-024-01610-2.

## Highlights

- Critical review of the polymers of intrinsic microporosity (PIM)-1-based membranes for applications in selective CO_2_ separation.
- State-of-the-art modification strategies for PIM-1 are thoroughly compared via meta-analysis.
- Key perspectives for progressing PIM-1 thin film membranes towards practical applications are suggested.

**Supplementary Information:**

The online version contains supplementary material available at 10.1007/s40820-024-01610-2.

## Graphical abstract

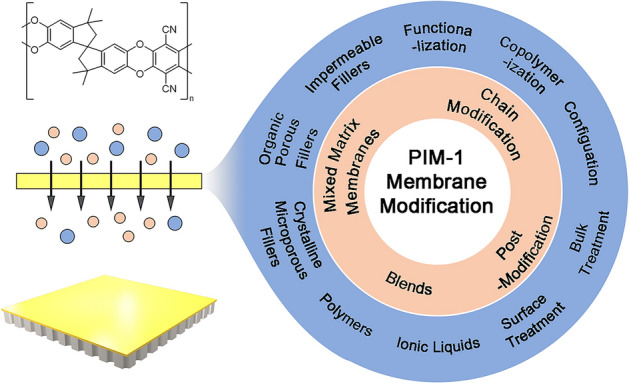

## Introduction to PIMs and PIM-1 Membranes

Polymers of intrinsic microporosity (PIMs) refer to a relatively new class of porous materials. The synthesis of these organic nanoporous materials, classified as microporous as their pore diameters are smaller than 2 nm, was first disclosed in a patent in 2003 [1] and later reported in the literature in 2004 [2]. The macromolecular backbone of the PIMs is composed of fused rings that prohibit large-scale conformational changes and incorporates sites of contortion, such as spiro-centres, giving rise to a randomly twisted structure that cannot efficiently fill space in the solid state [1]. Therefore, the fractional free volume in PIMs is high, and free volume elements are effectively interconnected, behaving like micropores. The bottleneck or gates interconnecting micropores behave as sieves for gas molecules with different sizes and shapes. Such interconnected microporosity is analogous to the framework structure of ordered molecular sieves such as zeolites. Additionally, such high free volume endows PIMs with a large accessible internal surface area (700–900 m^2^ g^−1^), giving rise to high sorption capacity for many molecules. The high adsorption capacity also contributes towards the fast transport of molecules, considering PIM-1 membranes follow the solution-diffusion model. Consequently, PIMs offer remarkable combinations of permeability and selectivity with higher values than most traditional synthetic polymeric membranes [3–5].

PIMs-based membranes are reported for various gas separation applications, including air separation [6], hydrogen recovery [7, 8], and other more challenging scenarios such as separation of ethylene (C_2_H_4_)/ethane (C_2_H_6_) and corrosive fluorinated gases [9]. Owing to their appropriate pore size and preferential adsorption towards carbon dioxide (CO_2_), PIMs membranes have been extensively investigated for selective CO_2_ separation. In 2005, the first gas permeation data of a prototype PIMs, PIM-1 membrane was reported, revealing a CO_2_ permeability of 2300 barrer, along with reasonable selectivity of 25 for CO_2_/nitrogen (N_2_) [10], which surpassed the upper bound established by Robeson in 1991 [11] and led to the revision of the upper bounds of performance in 2008 [3]. In following years, other PIMs with enhanced separation performance have been developed and have led to two more recent upper bounds [4, 5]. To date, over 600 publications have been published on CO_2_ separation using PIM-based membranes, with 80% of these published in the last decade. The exceptional high permeability (up to 50,000 barrer for CO_2_ [5]) and reasonably good selectivity makes PIMs membranes one of the most competitive candidates for their use in carbon capture and storage to achieve the net zero goals set by different governments [12].

Based on the solution-diffusion theory, enhancing gas sorption and increasing fractional free volume are two key aspects for boosting membrane selectivity and permeability [13]. Accordingly, various strategies are proposed and explored to modify PIM-1 membranes to improve gas separation performance. Furthermore, strategies for addressing physical aging and plasticisation, which are two major problems that hinders the commercialisation of PIMs membranes in industry, have been reported. Specifically, physical aging refers to the movement of polymer chains that interact by relatively weak van der Waals forces. Over time, the polymer chain of PIMs tend to densify due to the relaxation of non-equilibrium polymer chains to the equilibrium state, leading to what is known as physical aging [14]. This leads to a rapid degradation in permeability over time; for example, the CO_2_ permeability of self-standing PIM-1 membranes decreases by over 50% within 3 months [15, 16]. Although alcohols (such as methanol and ethanol) can used to partially recover the membrane permeance [17–19], the long-term effectiveness of such strategies is unclear, which needs significant research effort.

Plasticisation is another important problem that has significant practical implications in membrane-based gas separation [20]. The high sorption of condensable gases (e.g., CO_2_) at high pressure causes the polymeric matrix of PIMs to swell, leading to increased permeability of all gases, including the less permeable ones, and decreasing the membrane selectivity. The plasticisation is particularly important in natural gas sweetening, which is one of the fastest growing applications of membrane-based gas separation technology. The decrease in selectivity due to membrane plasticisation increases product methane (CH_4_) loss into the low-pressure permeate and compromises competitiveness against standard amine absorption methods.

For further improving gas separation performance and addressing physical aging and plasticisation, various PIMs with advanced chemical structures have been synthesised. These strategies focus on developing new chemistry of main chain of PIMs and have been extensively reviewed by other authors [6, 21–23]. On the other hand, the modification of existing PIMs polymers and membranes is another efficient route for achieving these same goals. To date, several reviews are available in the open literature, exemplified by [24–28], which light-touches one or a few modification strategies for PIMs-based membranes, without a systematic and comparative analysis to compare the effects of different strategies critically. Hence, a designated review on this aspect of PIMs polymers and membranes is needed to progress the field.

The protype PIM, PIM-1, has been recognised as the most extensively investigated of all PIMs and is highly representative in terms of modification strategies due to its relative simplicity of molecular structure, remarkable membrane-forming properties and good gas-separation performance. Herein, taking PIM-1 as an example, we present a comprehensive analysis of the modification strategies for improving membrane separation performance of PIMs membranes. Different strategies are summarised in this review, including (i) chain modification, (ii) post-modification, (iii) blending with other polymers, and (iv) filler addition (to synthesise mixed matrix membranes (MMMs)). In specific, the chain modification and post-modification represents the strategies of (i) PIM-1 chain engineering (such as grafting functional groups [29, 30]) and (ii) post membrane synthesis treatment of the PIM-1-based membranes (such as thermal crosslinking [31, 32]), respectively. To ensure a thorough and critical comparison, the meta-analysis of 150 + studies was conducted to systematically analyse and compare the effectiveness of various strategies concerning target membrane properties (such as permeability, selectivity, aging resistance, and plasticisation resistance). Note that meta-analysis is a statistical process combining data from multiple studies to identify common results and overall trends.

Finally, based on differences in specific purposes and fabrication methods, PIM-1-based membranes can be categorised into free-standing membranes and thin film composite (TFC) membranes. While free-standing membrane configurations (ranging from 40 to 100 μm) are well investigated due to their suitability for laboratory purposes, TFC membranes with thin selective layers (typically < 5 μm) are more desirable for industrial applications. These TFC membranes maximise productivity while offering the necessary mechanical properties for industrial gas separation. However, the differences in membrane configurations also impact their separation properties. Therefore, the development of PIM-1 thin film membranes is also reviewed and several key issues hindering the application of PIM-1 thin film membranes in gas separation applications are commented. This work provides a timely snapshot of the state-of-the-art modification strategies of the PIM-1 and their effectiveness and offers perspectives to guide further research to advance the development of PIMs membranes for potential practical applications in gas separation.

## PIM-1 Membrane Modification

**Fig. 1:**
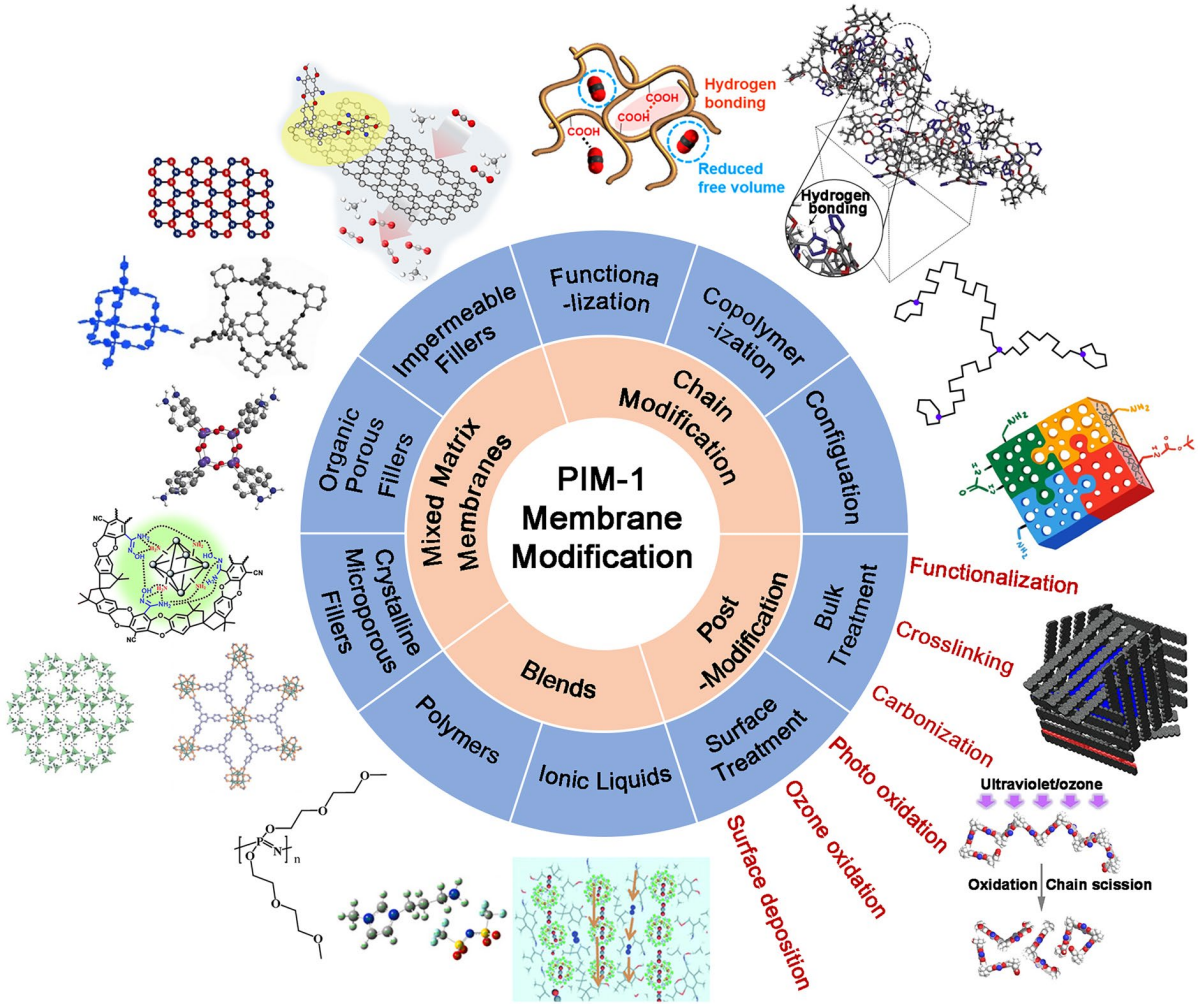
Schematic of the developed modification strategies for PIM-1 membranes

The modification strategies of PIM-1 include chain modification, post-modification, blending with other polymers, and the addition of fillers has been performed for improving gas separation performance and addressing problems of physical aging and plasticisation, as shown in Fig. 1. We will comment on them with specific examples.

### Chain Modification

**Fig. 2:**
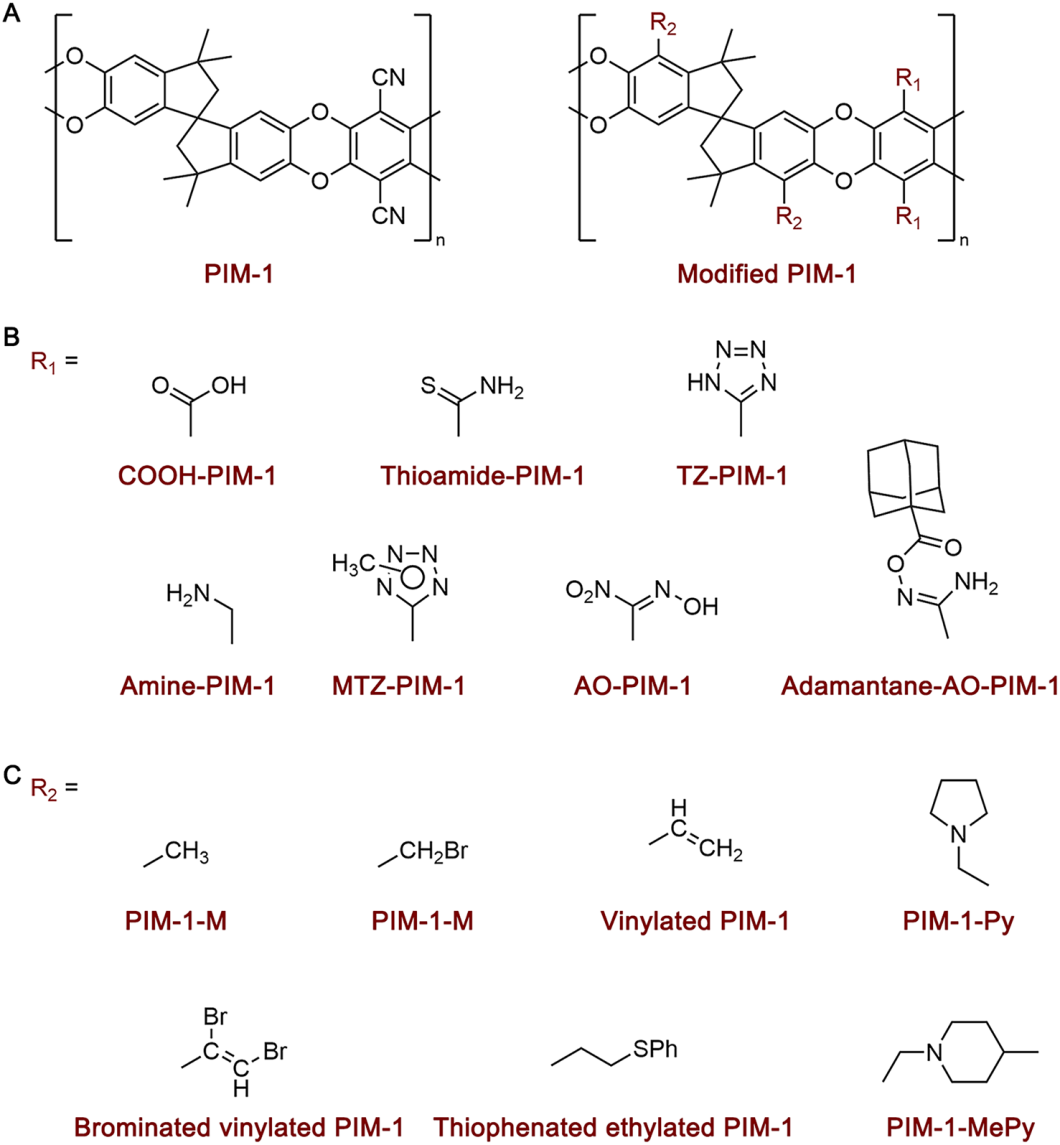
Chemical structures of the typical modified PIM-1 polymers (scheme for the modification (**A**) and typical functional groups introduced by functionalisation of as-synthesised PIM-1 chains (**B**) and by copolymerisation (**C**)

Chain modification of PIM-1 refers to altering the chemical structure of PIM-1 through chemical reaction (Fig. 2). The cyano (− CN) groups of the PIM-1 structure (R_1_ in Fig. 2A) can be transformed into a variety of functional groups, thus providing a great opportunity for chemical modifications and functionalised design of the as-synthesised PIM-1. Examples of the functional groups include carboxylic acid (− COOH) [33–36], thioamide (–thio) [37], tetrazole (–TZ) [30], amino (− NH_2_) [38], methyl tetrazole (–MTZ) [39], amidoxime (–AO) [29], and adamantane [40] (Fig. 2B). Another strategy for PIM-1 modification is copolymerisation with monomers with specific groups (R_2_ in Fig. 2A). Examples include substituting the hydrogen groups in spirobisindane moieties with different side groups including methyl (− CH_3_ [41]), bromomethyl (− CH_2_Br [41]), vinyl (− CH = CH_2_ [42]), brominated vinyl (− CHBrCH_2_Br [42]), thiophenated ethyl (− CH_2_CH_2_SPh [42]), pyrrolidinated methyl (− Py [41]), and 4-methylpiperidinated methyl (− MePi [41]) (Fig. 2C).

Functional groups interacting selectively with a specific guest gas molecule can enhance the selectivity of PIM-1 membranes. Such interactions are expected to enable the selective adsorption of specific molecules over others (e.g., CO_2_ over N_2_), thus increasing membrane selectivity. For example, after modifying PIM-1 with CO_2_-affinity − TZ groups, the CO_2_ adsorption of polymers increased by approximately 10%, whilst N_2_ adsorption decreased by ~ 95%. Additionally, the strong sorption of CO_2_ molecules on − TZ groups could exclude the sorption or passage of lighter gases in membranes. As a result, the CO_2_/N_2_ selectivity of TZ − PIM-1 membranes is 2.5 times higher than the PIM-1 counterpart [30]. However, it is very difficult to increase selectivity without compromising permeability by this strategy. Commonly, secondary interactions could be induced by the presence of functional groups (e.g., hydrogen bonding) leading to polymer matrix contraction, and thus the hindered molecular diffusion and decreased membrane permeability. Such trade-off between selectivity and permeability was observed in various modified PIMs, including TZ − PIM-1 [30], Thio − PIM-1 [37], AO − PIM-1 [29], COOH − PIM-1 [33], and PIM-1 − NH_2_ [38], with membrane permeability decreased by 70% − 90% compared to their pure PIM-1 counterparts.

As PIM-1 has a “bimodal” pore size distribution, that is, ultra-micropores (< 7 Å) serves as molecular sieves for separating gas molecules with different sizes and shapes, and micropores (7 − 20 Å) facilitate rapid gas diffusion. It was found that the side groups in spirobisindane moieties mainly fill the micropores having insignificant effect on ultra-micropores, and therefore, they could reduce the permeability but showing little effect on selectivity [41]. Conversely, modification of the − CN groups on PIM-1 chains could improve the membrane selectivity effectively [33–36], leading to the hypothesis of ultra-micropores being related to the interaction between − CN groups, and hence, modifying the − CN groups on the PIM-1 chains is more effective to improve the separation selectivity.

The incorporation of specific functional groups into PIM-1 could also improve the rigidity of polymer chains and alleviate membrane plasticisation through the secondary interactions. For example, − AO groups in PIM-1 could introduce pervasive intermolecular hydrogen bonding networks within the membrane, mitigating CO_2_-induced matrix dilations. It was reported that these modifications maintain stable mixed-gas selectivity over a pressure range of 20 bar (compared to 8 bar for pure PIM-1) [29]. In addition to the functionalisation approaches discussed above, a cyclic-locked (defined here as PIM-C1) was also developed by introducing intramolecular locking on PIM-1 (between R_2_ groups in Fig. 2A). This strategy enhanced the rigidity and reduced the packing density of the polymer, thereby achieving simultaneous improvements in both permeability and selectivity for CO_2_ separation [43, 44].

Moreover, simply adjusting the synthetic conditions of PIM-1 polymers result in a series of PIM-1 polymers with different physical properties (e.g., molar mass and interconnected colloidal network content) and structural features (e.g., branching and ring topology), which consequently affect their gas transport properties in membrane separation. Foster et al. [45] reported membranes cast from solutions of PIM-1 samples containing high proportions of branched and colloidal network content which exhibited higher selectivity for CO_2_/CH_4_ and CO_2_/N_2_ gas pairs. Additionally, PIM-1 with small loops (< 20 nm) demonstrated improved separation performance (CO_2_ permeability of 4835 barrer and CO_2_/N_2_ selectivity of 55.5) due to the high rigidity of the small loops which enhanced gas selectivity [46].

### Post-Modification

In most cases, membranes composed of functionalised PIM-1 have a lower fractional free volume, thus restricting molecular diffusion. This is because that the secondary interactions introduced by the new functionality increase the attraction between the chains, thus decreasing the fractional free volume. Post-synthetic functionalisation could circumvent this issue, as the movement of the polymer chains caused by the secondary interaction of new functionalities is relatively restricted in solid-state membranes. Therefore, post-modification can leverage the sorption benefits of post-synthesised functionalisation while preserving the beneficial properties provided by the PIM-1 backbone [47]. Furthermore, post-modification also introduces the possibility to synthesize asymmetric membrane by surface treatment for optimizing the gas separation performance. Additionally, given that the chain modification methods typically involve chemical reactions and rather complex purification steps, post-modification offers the advantage of simplicity with less requirements for additional purification steps.

#### Bulk Treatment

##### Incorporating Functional Groups

**Fig. 3:**
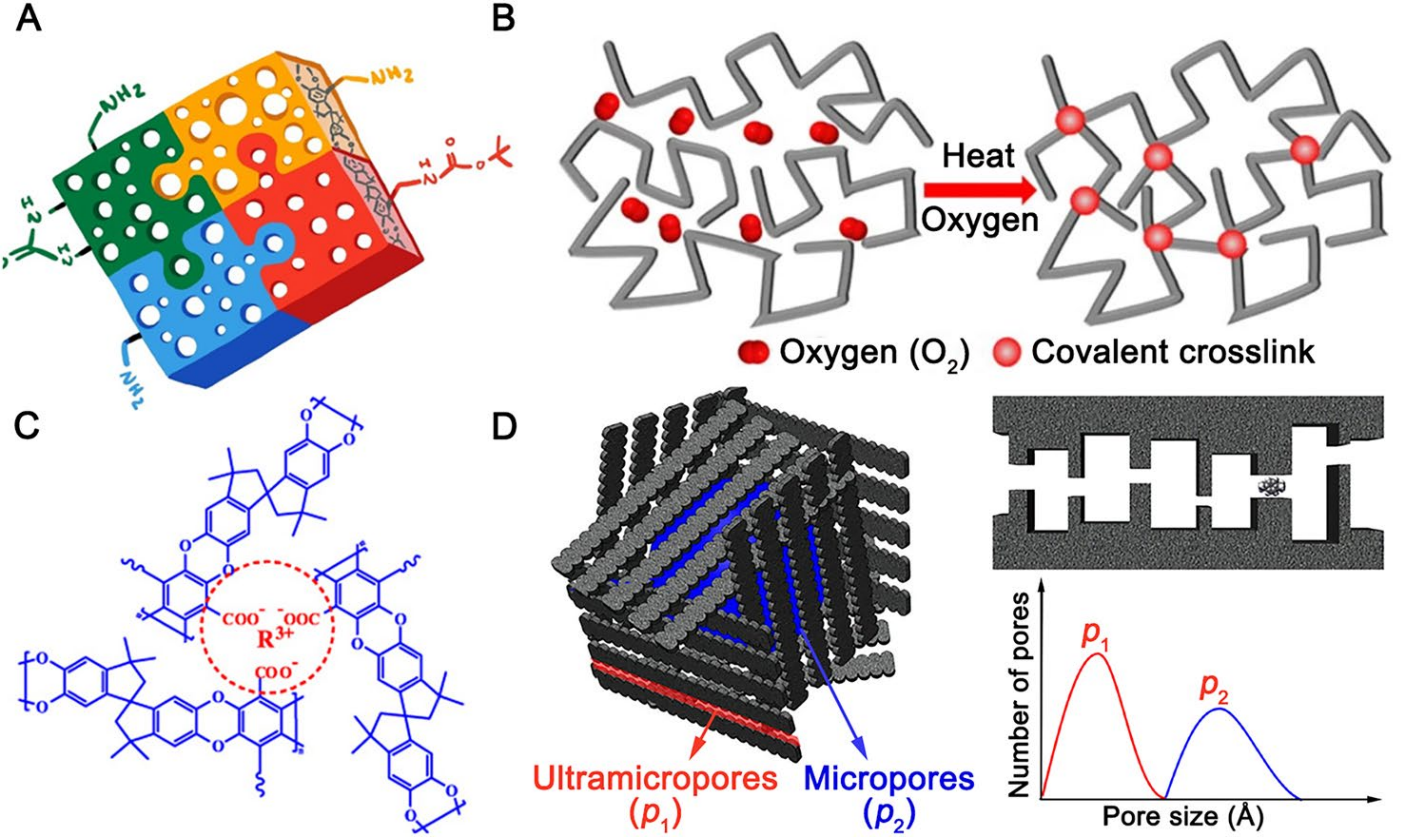
Strategies of post-modification of PIM-1 (**A**: post chemical modification; **B**: thermal crosslinking; **C**: metal ions crosslinking; **D**: carbonisation). Adapted from Refs. [32, 47, 56, 63] with permission

Similar to the modification of the PIM-1 chain, most efforts in post-modification have been concentrated on altering the − CN groups on PIM-1. In 2021, Rodriguez et al. [47] reported a series of modified PIM-1 including PIM-1 − NH_2_, PIM-1 − *t*BOC, and PIM-1 − deBOC(acid) by chemical modification (Fig. 3A). Following this, sulfonated (− SO_3_H) [48], aminated [49], amidated [50], and carboxylated (− COOH) [50] PIM-1 were also reported. The CO_2_ selectivity was typically improved due to the improved CO_2_-philicity and tailored passageway (e.g., strong hydrogen bonding in the modified PIM-1 membrane matrix induce the formation of additional size-sieving ultra-micropores [48]). For example, the post-aminated PIM-1 demonstrated a CO_2_ permeability of 2590 barrer and a CO_2_/N_2_ selectivity > 30 [49].

In addition, Ma et al. [7] reported a feasible fluorination strategy. Unlike other chemical modifications methods that altered the − CN groups on PIM-1, fluorine (0.57 Å) replaced the hydrogen atoms (0.31 Å) in PIM-1. This replacement largely preserved the intrinsic microporosity but reduced the effective average pore size of PIM-1, consequently enhancing the kinetic separation efficiency. Meanwhile, the introduction of C − F bonds in the micropores also improved the sorption selectivity of hydrogen (H_2_)/CH_4_, resulting in a record-high performance in the application of helium (He) enrichment from natural gas (with He/CH_4_ and H_2_/CH_4_ selectivity of 3770 and 1630, respectively) [7].

##### Crosslinking

The decomposition temperature of PIM-1 is approximately 415 °C. Below this temperature, thermal treatment of the PIM-1 (at 300 − 400 °C) induces inherent crosslinking [31]. Typically, thermal crosslinking tends to draw the polymer chains closer, resulting in a decrease in free volume [31]. Moreover, the improved rigidity of the polymer chains in the membrane constrained by the crosslinking, increased the selectivity for CO_2_/CH_4_ by approximately fourfold to 54.8. Additionally, in the presence of trace amounts of oxygen, oxidative crosslinking of PIM-1 chains was reported to occur at the ultra-micropores of PIM-1 membranes [32]. This oxidative crosslinking resulted in narrower sieving gates, offering remarkably improved size and shape selectivity (CO_2_/CH_4_ selectivity up to 70) (Fig. 3B).

Besides the thermal crosslinking of PIM-1, crosslinking reagents (e.g., diazides [51]) and catalysts (e.g., trifluoromethanesulfonic acid (TFSA) [52]) have also been used to form crosslinked PIM-1. Additionally, thermal crosslinking of PIM-1 − COOH [53], mono-esterified PIM-1 [54], bromoalkylated PIM-1 [55], and PIM-1 − deBOC [47] have been reported. These strategies can reduce the crosslinking temperature of PIM-1, shorten the crosslinking time, and regulate the pore size distribution. Notably, the crosslinking could endow PIM-1 membranes with improved anti-plasticisation properties, due to the strong interactions that prevented the movement of the polymer chains. For example, the crosslinked mono-esterified PIM-1 membrane exhibits good plasticizing resistance under feed pressure up to 42 bar [54].

Metal ions were also introduced into modified PIM-1 (e.g., carboxylated PIM-1) [56, 57] (Fig. 3C). These metal ions provided selective adsorption of propylene (C_3_H_6_) over propane (C_3_H_8_) due to the formation of strong π-complexation between C_3_H_6_ and the unsaturated metal sites. However, metal ion-induced crosslinking has little effect in improving the membranes’ anti-plasticisation performance. This might be due to the lower binding energy of the coordination bonding between the metal ions and the − COOH compared to the covalent bonding formed by thermal/chemical crosslinking. Theoretically, stronger interactions can help to maintain the structural integrity of a membrane and prevent plasticisation [58].

##### Carbonisation

Above 500 °C and in an inert atmosphere, PIM-1 membranes undergo pyrolysis and form carbon molecular sieve (CMS) membranes. CMS membranes have rigid, size-sieving pore structures and a large array of ultra-micropores, which helps in distinguishing permeants based on the small differences in their molecular dimensions (Fig. 3D).

Pyrolysis has been successfully applied to PIM-1 membranes, which have shown good separation performance for C_2_H_4_/C_2_H_6_ [59–61], C_3_H_6_/C_3_H_8_ [62] and *p*-xylene/*o*-xylene gas mixtures [63, 64]. Incorporating thermally labile *β*-cyclodextrin (β-CD) and bulky boron compounds into PIM-1 can increase the permeability of the CMS due to the formation of additional micropores upon pyrolysis [61, 62]. Similar to PIM-1, the PIM-1-derived CMS also has a “bimodal” pore size distribution, with narrow gates (ultra-micropores) for remarkably better size and shape selectivity, while the micropores are maintained sufficiently high for rapid gas diffusion (Fig. 3D). For example, Ma et al. [63], reported that the PIM-1-derived CMS pyrolysis at 550 °C exhibited high *p*-xylene/*o*-xylene diffusion selectivity and comparable diffusivity to that in MFI zeolites. Specifically, the ultra-micro slit-type channels in the CMS require higher energy for *o*-xylene to pass through compared to the smaller molecule, *p*-xylene, while the micropores inside the CMS provide little resistance to the diffusion of guest molecules. Furthermore, in another work they found that pyrolysis of PIM-1 in an H_2_ environment resulted in larger ultra-micropore dimension, referred to as “mid-sized” micropores [64]. This led to a drastic increase in *p*-xylene permeability with a slight loss in *p*-xylene/*o*-xylene selectivity.

In addition, Jue et al. [60] also demonstrated that PIM-1 can be utilised to fabricate CMS hollow fibre membranes (HFMs). Due to the high glass transition temperature of PIM-1 (442 °C, higher than the polymer decomposition temperature of 415 °C), the PIM-1 HFMs remain in the glassy state throughout pyrolysis. This property is critical for maintaining an asymmetric membrane structure during pyrolysis. The synthesised HFMs displayed higher ideal selectivity than their reported corresponding flat sheet counterpart pyrolysed at the same temperature, and met/exceeded the 2008 upper bounds for gas pairs including CO_2_/CH_4_ [3].

An intermediate annealing strategy at 450 °C was developed by He et al. [65]. The PIM-1 membrane was crosslinked, while simultaneously, the stacking of small molecules produced by pyrolysis partially filled the pores. The optimised membrane exhibited exceptionally high selectivity (CO_2_/CH_4_ selectivity of 200), although the permeability was reduced by over 2 orders of magnitude.

#### Surface Treatment

Integral modification of PIM-1 membranes may reduce molecular diffusion due to secondary interactions introduced by the new functionalities or structural changes. However, surface modification may confer enhanced size sieving on the membrane surface without affecting the permeability of the bulk membrane. Additionally, modification of the membrane surface could exacerbate the difference in molecular sorption between the upside and the downside of the membrane, thereby increasing the mass transfer driving force and facilitating permeation. The simplicity and time-saving of surface modification is also more advantageous than the other techniques. Typical strategies of surface treatment include:

##### Photo Oxidation

**Fig. 4:**
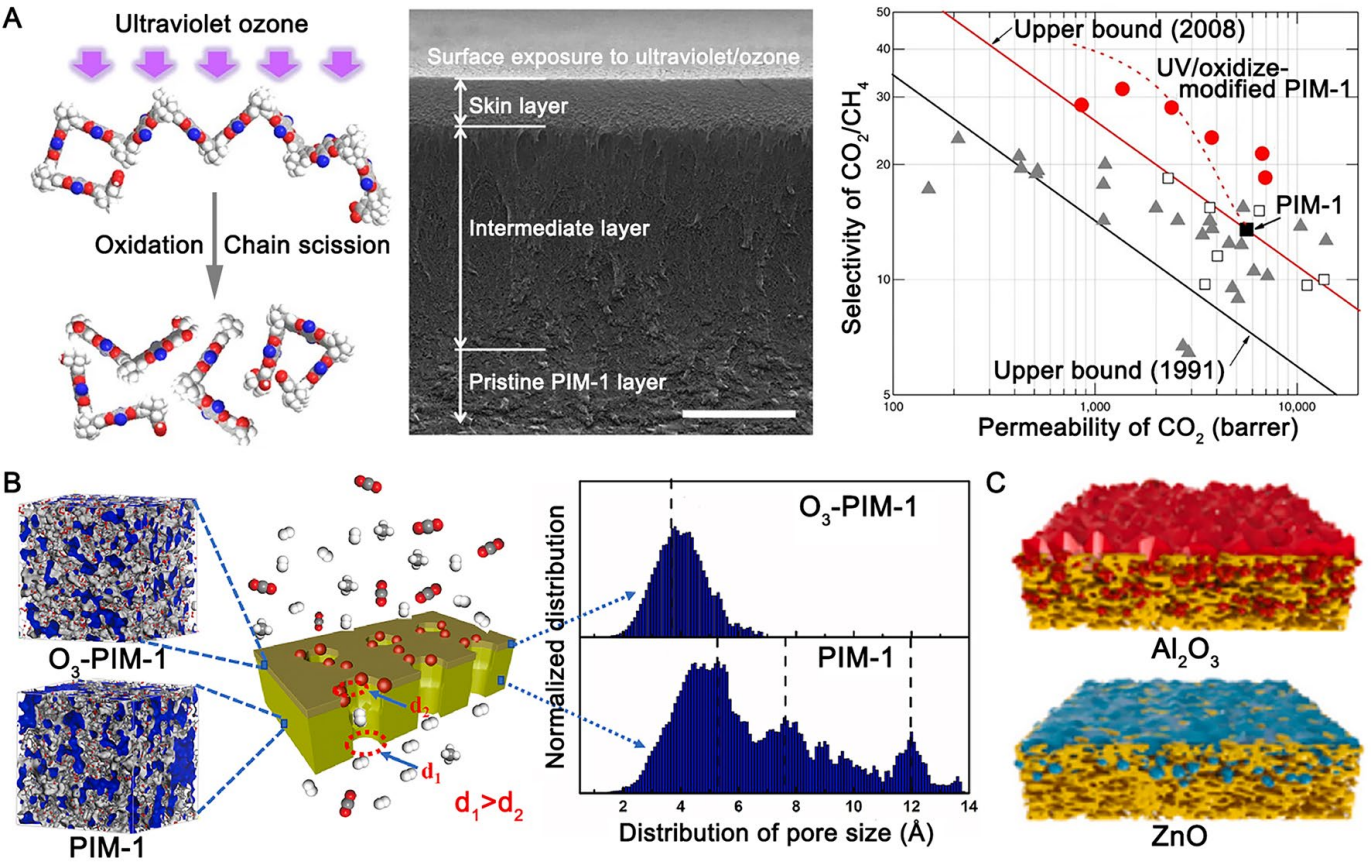
Surface treatment of PIM-1 by **A**: photo-oxidation, **B**: ozone-oxidation, and **C**: surface deposition). Adapted from Refs [66, 69, 70]. with permission

Photo oxidation is a simple and powerful method to enhance the gas-separation performance of membranes, especially the high free-volume polymeric ones, owing to their inherently greater potential for structural collapse than conventional denser polymers. Song et. al. [66], and Li et al. [67], reported the photo-oxidative enhancement of PIM-1 membranes by ultraviolet (UV) irradiation in air (Fig. 4A). Under short-wavelength UV irradiation (wavelength of 254 nm), processes such as chain scission [66], the formation of carbonyl and hydroxyl groups [66], and the destruction of the spiro-carbon centre [67] can occur in the presence of oxygen. These processes lead to the shrinkage of large micropores and narrow the free volume distribution. This phenomenon has been observed in PIM-1 membranes when exposed to a UV light field, which induces photo-oxidation with a penetration depth of ~ 500 nm. This leads to the densification of membranes and improved selectivity for gas separation, specifically, a CO_2_/N_2_ selectivity of 27.7 (CO_2_ permeability of 3781 barrer) and 5 to 7 times higher H_2_/N_2_ and H_2_/CH_4_ selectivity [66].

Hou et al. [68], further investigated the photo oxidation of PIM-1. It was found that extended exposure time could further increase selectivity (i.e., a 24-fold improvement for H_2_/CH_4_ selectivity, from 5.4 to 127, after 4.5 h of exposure), with reduced membrane permeability (i.e., an 81% decrease in H_2_ permeability down to 1028 barrer). It is worth noting that photo oxidation had a bigger effect on thinner PIM-1 membranes (minimum 9 μm) due to the higher ratio of the densified skin layer. By incorporating the porous filler PAF-1, the reduction of permeability during photo oxidation could be partially compensated, resulting in a high H_2_ permeability of 4800 barrer along with a high H_2_/CH_4_ selectivity of 90.

##### Ozone Oxidation

Ji et al. [69], reported a simple direct ozone oxidation technology to improve the size sieving and H_2_ separation performance of PIM-1 (Fig. 4B). The O_3_ oxidisation occurred in seconds and introduced C = O and − COOH groups on the PIM-1 chains. Thus, according to molecular simulation, this altered the pores to single and narrow ultra-micropores (3.8 Å). The fast oxidation speed and the narrow pore sizes prevented the transfer of ozone (O_3_) and made the ozone oxidation technology self-terminating, with the oxidation depth below 10 μm. Combining the size-sieving pores on the surface and large pores in the bulk, the O_3_-treated PIM-1 membrane showed a H_2_ permeability of 1294 barrer and a H_2_/CH_4_ selectivity of 121.

##### Surface Deposition

In the past decade, the atomic layer deposition (ALD) technique has emerged as a promising new route for membrane surface functionalisation due to its combined advantages of exceptional conformality and thickness control at the nanoscale. Chen et al. [71], pioneered the application of ALD for the deposition of aluminium oxide (Al_2_O_3_) on the surface of PIM-1 to tailor its micropores at the sub-nanometre scale, leading to a prominent size-sieving effect on the membrane surface (Fig. 4C). The performance of the optimised membranes far exceeded the latest trade-off lines for H_2_/N_2_, H_2_/CH_4_, CO_2_/CH_4_, and O_2_/N_2_ gas pairs (e.g., H_2_/CH_4_ selectivity of 225 and H_2_ permeability of 2492 barrer). Titanium dioxide (TiO_2_) and zinc oxide (ZnO) were also deposited in the micropores of PIM-1 [70]. Unlike membranes with Al_2_O_3_ and ZnO (diffusion-dominated enhancements obtained), higher affinity of TiO_2_ for CO_2_ increased the CO_2_ adsorption on the membrane surface, hence enhancing the mass transfer driving force and therefore increasing the CO_2_ permeability and the CO_2_/CH_4_ selectivity. Alongside the ALD, polymerisation of tannic acid (TA), which contains abundant oxygen-containing groups, was reported on another work to form a CO_2_-affinity surface on PIM-1 membranes and enhance its mass transfer [72].

### PIM-1 Mixed Matrix Membranes (MMMs)

#### Mechanism of Mass Transfer in MMMs

According to the Maxwell model, the gas permeability of MMMs is a combination of the permeability through the bulk matrix and through the inner pores of fillers as Eq. (1).

1 $$P_{{{\text{MMM}}}} = P_{{\text{m}}} \frac{{P_{{\text{f}}} \left( {1 + 2\varphi_{{\text{f}}} } \right) + P_{{\text{m}}} \left( {2 - 2\varphi_{{\text{f}}} } \right)}}{{P_{{\text{f}}} \left( {1 - \varphi_{{\text{f}}} } \right) + P_{{\text{m}}} \left( {2 + \varphi_{{\text{f}}} } \right)}}$$

where* P*_MMM_, *P*_m_, and *P*_f_, are the permeability of the MMM, the polymer, and the filler, respectively; *φ*_f_ is the volume fraction of the filler. However, the mass transfer of PIM-1 MMMs differs from theoretical calculation. Apart from the mass transfer through the aforementioned 2 pathways, 3 other pathways are also important in the mass transfer in MMMs.

**Fig. 5:**
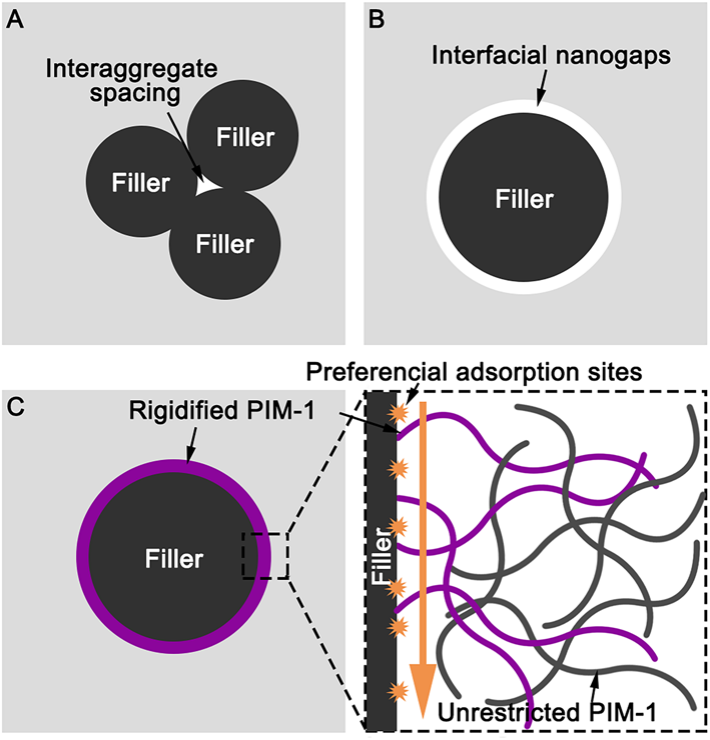
Schematic of mass transfer through the rigidified PIM-1 in the vicinity of fillers (**A**: interaggregate spacing; **B**: interfacial nanogaps; and **C**: interfacial rigidification)

(i) The interaggregate spacing. The less compatibility between the filler and the polymer would lead to the aggregation of the filler, and interaggregate spacing would be formed between the aggregates, which becomes bigger at higher particle concentrations (Fig. 5A). Aggregation can create unfavourable non-selective shortcuts in the membrane cross section, which can seriously affect membrane selectivity.
(ii) Interfacial nanogaps. The polymer chains may not tightly contact the fillers if the compatibility between them is low, thus forming a narrow gap surrounding the fillers (Fig. 5B). The gas diffusion path is shortened, resulting in increased gas diffusivity and permeability. However, this is not a favourable scenario as the gaps are non-gas-selective.
(iii) The presence of the nanofiller restricts the conformational freedom of polymer chains in its vicinity and frustrates the ability of the chains to pack together, i.e., rigidification (Fig. 5C). This can alter the fractional free volume of the membrane and, simultaneously, prevent the physical aging of PIM-1. Additionally, as molecules pass through, the selective affinity sites or functional groups on the filler can preferentially adsorb the target gases (e.g., CO_2_), thus improving membrane selectivity.

Overall, high compatibility between the filler and the polymer matrix is necessary for avoiding unselective interaggregate spacing and interfacial nanogaps. With homogeneous dispersion and in the absence of significant interfacial gaps, the MMMs can not only combine the mass transfer characteristics of the polymer and the filler, but also create rigidified regions at the interface between the fillers and the polymers. This can further improve the fractional free volume, prevent physical aging, and potentially lead to additional selectivity for certain gases (Fig. 5).

#### Impermeable Fillers

For impermeable fillers lacking inner mass transfer pathways, the interfacial interaction between the filler and the polymer matrix is of vital importance. Inorganic fillers in general lack strong interfacial interaction with PIM-1, susceptible to form interfacial nanogaps and interaggregate spacing. For example, without surface functional groups, adding 23.5 wt% of silica increased the CO_2_ permeability to 13,400 barrer but halved the CO_2_/N_2_ selectivity value (7.5) [73]. With abundant surface functional groups, organic impermeable fillers have higher compatibility with PIM-1, due to stronger interfacial interaction such as hydrogen bonding between the functional groups and PIM-1 chains, thus reducing interfacial defects. A typical example of organic impermeable filler is polydopamine-derived submicron spheres (PDASS), which improved the CO_2_/N_2_ selectivity to 35 in PIM-1 MMMs, while the permeability decreased to 1678 barrer [74]. Introducing organic functionalities such as − OH [75], − SO_3_H [75], and 3,5-dimethylbenzoic acid [76] on inorganic fillers, can also introduce improved interfacial interaction through hydrogen bonding between the functionalities and PIM-1. Moreover, stronger covalent bonding between inorganic fillers (e.g., graphene oxide (GO)) and PIM-1 was also achieved by *in-situ* synthesis of PIM-1 on GO [77].

Without interfacial defects, the permeability of the membrane typically decreases due to the rigidification of PIM-1 chains and the prolonged mass transfer path. However, some surface functionalities not only help with the improvement of interfacial compatibility between inorganic fillers but also promote preferential sorption of CO_2_, facilitating the selective mass transfer. As reported by Moshenpour et al. [75], the addition of sulfonic acid-functionalised silica nanosheets led to an increased CO_2_ permeability by 41%, while increase the CO_2_/N_2_ selectivity by 18% [76].

In order to leveraging the fillers’ CO_2_-affinity sites on their surface, a large interfacial area is preferred. Two-dimensional (2D) fillers such as GO [77–81], graphitic carbon nitride (g-C_3_N_4_) [82, 83] and nitrogen (N)-doped porous carbon [16], possess high surface area-to-volume ratios and thus allow high interfacial areas at very low loadings. Using these 2D fillers with CO_2_-affinity sites such as basic N-sites [16, 82–84] and functionalities including sulfonated groups [83], tris(4-aminophenyl)-amine (TAPA) [79], and polyhedral oligomeric silsesquioxane (POSS) [80], the CO_2_ permeability of the MMM significantly increased with relatively good selectivity (e.g., CO_2_ permeability of up to 40,544 barrer with CO_2_/N_2_ selectivity of 12.4 for the PIM-1 membrane containing N-doped porous carbons [16]). Additionally, MXene nanosheets have been utilised as a 2D filler in PIM-1. Compared to the other 2D materials, MXene offers the advantage of highly customisability, which provides significant potential for developing high-performance membranes. However, further research should focus on addressing some drawbacks, such as low cost-effectiveness, low scalability, and complex synthesis procedures [85].

In addition to improving membrane permeability/selectivity, the addition of impermeable fillers can also reduce the physical aging of PIM-1. This is due to the rigidification of the PIM-1 chains in the vicinity of the filler that does not allow them to pack together. Particularly, 2D fillers show the most prominent effect in anti-aging by maximising the interfacial areas. Furthermore, functionalities on the 2D filler can further enhance the anti-physical aging performance due to the improved filler/polymer interaction. Gorgojo et al. [77–81] reported PIM-1 MMMs with a series GO-based fillers including GO functionalised by octylamine (OA), octadecylamine (ODA), TAPA, and PIM-1 chains. It was found that they all showed improved anti-aging performance compared to bare GO in the PIM-1 MMMs. Among them, fillers with long functional chains showed a more prominent effect, likely due to the intertwining of long functional chains with PIM-1 further expend the rigidified region in PIM-1 matrix. In particular, PIM-1-functionalised GO showed the most effective anti-aging property, with the CO_2_ permeability only reduced by 15% after 150 days [81].

#### Organic Porous Fillers

**Fig. 6:**
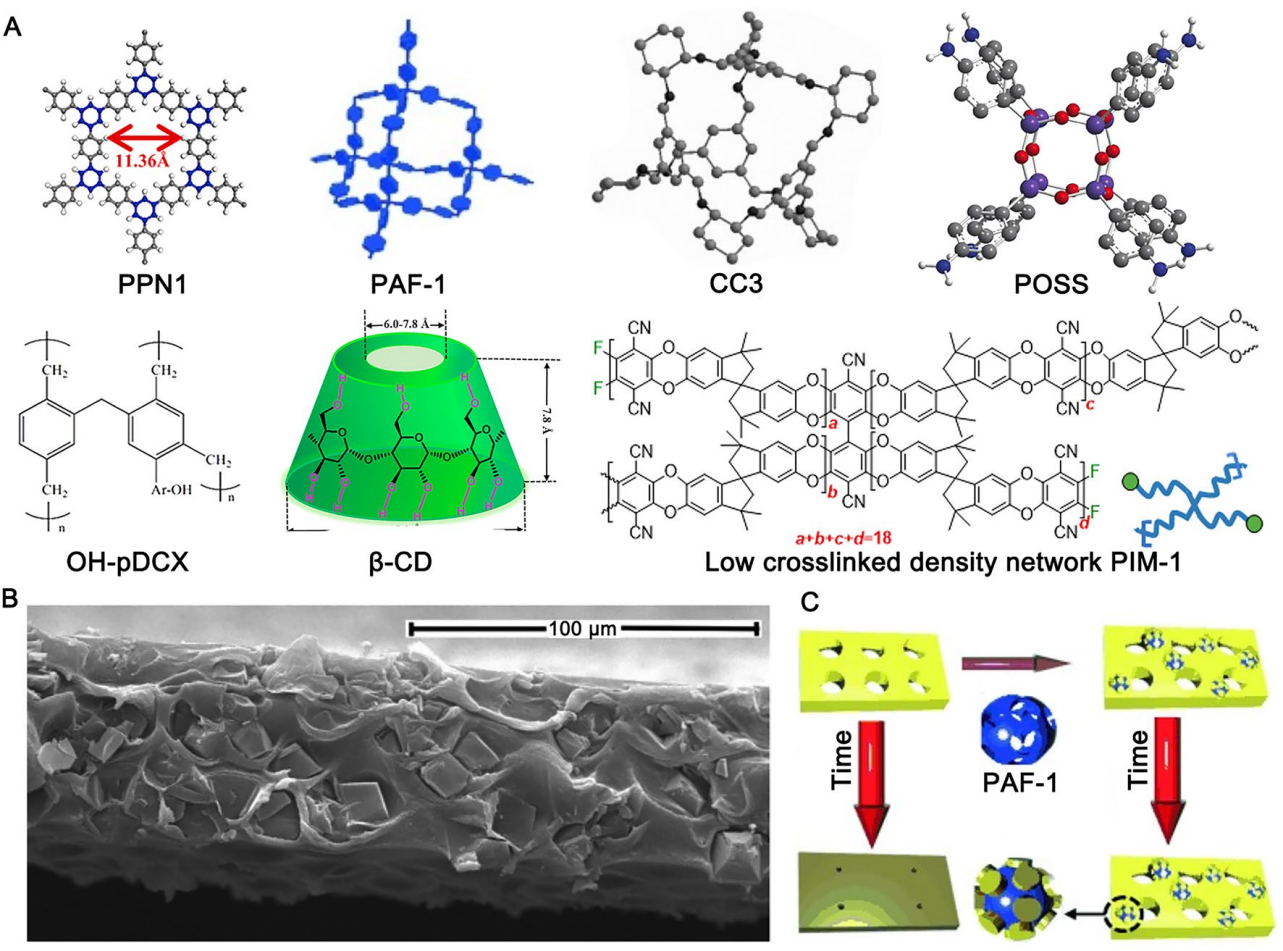
**A** Different organic porous fillers; **B** *in-situ* crystallisation of CC3 in PIM-1 matrix; **C** schematic for physical interaction between PIM-1 and PAF-1 for preventing membrane aging. Adapted from Refs [86, 90, 92, 93, 95, 100, 102]. with permission

Organic porous fillers are composed of pure organic components and are linked together via covalent bonds to form the porous structures. Their organic composition makes them highly compatible to PIM-1, and thus ideal candidate for acting as fillers. Additionally, organic porous fillers possess physicochemical properties such as diversity of chemical structures, high surface areas, and tuneable pore sizes. Various of organic porous fillers has been investigated, including PPN1 [86], TPFC [87], BILP-101 [88], MAPDA [89], porous aromatic frameworks (PAF) [90, 91], porous organic cages (CC3 [92]), β-CD [93], hyper crosslinked polystyrene (HCP [94], including pDCX [95]), boron icosahedron (K_2_B_12_H_12_) [96], and POSS and its derivatives [80, 97–100] (Fig. 6A). Additionally, PIM-1-based fillers, such as network PIM-1 nanosheets [101] and low crosslinking density (LCD) network PIM-1 [102] have also been developed for PIM-1 MMMs.

Compared with other fillers, organic porous fillers exhibit high dispersity and superior intrinsic high compatibility with PIM-1, thereby eliminating non-selective voids. Certain organic porous polymers, such as CC3 [92], can even crystallise *in-situ* from the PIM-1 casting solution, thereby further enhancing their dispersibility compared to traditional “blend and cast” strategies for fabricating MMMs (Fig. 6B).

Organic porous fillers with high porosity and large pore sizes can create mass transfer shortcuts in the continuous PIM-1 matrix, facilitating rapid diffusion and increasing membrane permeability. Among them, the addition of pDCX [95] and CC3 [92] fillers resulted in the highest CO_2_ permeability for MMMs, reaching 20,500 and 18,780 barrer, respectively, with minimal decrease in selectivity. In addition, introducing CO_2_-affinity functional groups into the organic porous polymers not only boost selective mass transfer at the filler/polymer interface by preferential sorption of CO_2_, but also in the porous structures inside the fillers. Therefore, the functionalisation of organic porous polymers exhibits a more prominent effect than that of impermeable fillers in enhancing the PIM-1 membrane selectivity. For example, MMMs containing CH_2_NH_2_ − TFPC [87], PEG − POSS [98], and OH − pDCX [95] showed improvements in CO_2_/N_2_ selectivity of 76%, 90%, and 54%, respectively, as compared to MMMs with bare TPFC [87], POSS [97], and pDCX [95], reaching values of 45.9, 30.4, and 28.0, respectively.

The high dispersity and compatibility of organic porous fillers with PIM-1 also endow them with superior resistance to the physical aging of PIM-1 compared to other fillers [86]. In addition, some organic porous fillers exhibit unique resistance to the physical aging of PIM-1. Lau et al. [90] reported a “physical crosslinking” phenomenon where PIM-1 chains could be partially inserted into the PAF-1 due to their large windows (Fig. 6C). This interaction holds the PIM-1 chains in their open position, thereby preventing aging. This strategy demonstrates the best anti-aging performance for PIM-1 thick membrane to date, with only a 7% decrease in CO_2_ permeability over 240 days (vs. 42% for the pure PIM-1).

#### Crystalline Microporous Fillers

Crystalline microporous fillers are the most promising category due to their rigid and ordered repeating network of identical structure. This structure enables strict size sieving of molecules, thereby offering potentially very high selectivity, with a wide range of permeability depending on their versatile pore sizes and apertures [103]. Additionally, the porous structure of crystalline microporous fillers, with confined spaces and large surface areas, intensifies the selective sorption of the affinity groups to certain molecules (e.g., CO_2_), thereby enhancing their role in facilitating preferential mass transfer. In particularly, among the large varieties of crystalline microporous fillers, some of them have pore sizes larger than most gases (such as silicalite-1, with pore size > 5 Å), therefore barely have size-sieving selectivity. However, they still have demonstrated improved selectivity for CO_2_/N_2_, which could be due to the facilitating effect due to the preferential sorption sites in the frameworks, and rigidification of the PIM-1 in the vicinity of the fillers [104].

Metal organic frameworks (MOFs) are the most prominent crystalline microporous fillers in PIM-1-based membranes. They offer advantages over zeolites and covalent organic frameworks (COFs) due to their ease of preparation, and chemical diversity, high compatibility with polymers (as they are composed of metal nodes and organic linkers), and ease of morphological manipulation. Various MOFs have been introduced into PIM-1 membranes, including ZIF-8 [105–109], ZIF-71 [110], UiO-66 and its derivatives [15, 111–123], MOF-74 [124], MIL-101 and its derivatives [125, 126], MFM-300 [127], NUS-8 and its derivatives [16, 128–130], DMOF-1 [131], ZIF-67 [132], MOF-801 [133], MUF-15 [134], ZIF-7 and its derivatives [135], ZIF-67 and its derivatives [136–138], MOF-33 [139], KAUST-7 [140], BcoC-ZIF [141], and ZIF-62 [142].

Due to their high chemical and structural versatility, MOFs can be optimised by adjusting synthetic techniques or be modified post synthesis to enhance the affinity for target molecules (such as CO_2_), thereby improving the separation performance [118, 143]. This includes incorporating CO_2_-affinity functional groups (e.g., amino [114] and azobenzene [115]) and functional compounds such as ions (e.g., Ag^+^ [119], Ti^+^ [111]), ionic liquids (e.g., TSIL [126], [HDBU][Im] [137], [NH_2_-Pmim][Tf_2_N] [138]), and metal complex (e.g., Ag_3_pz_3_ [144]).

**Fig. 7:**
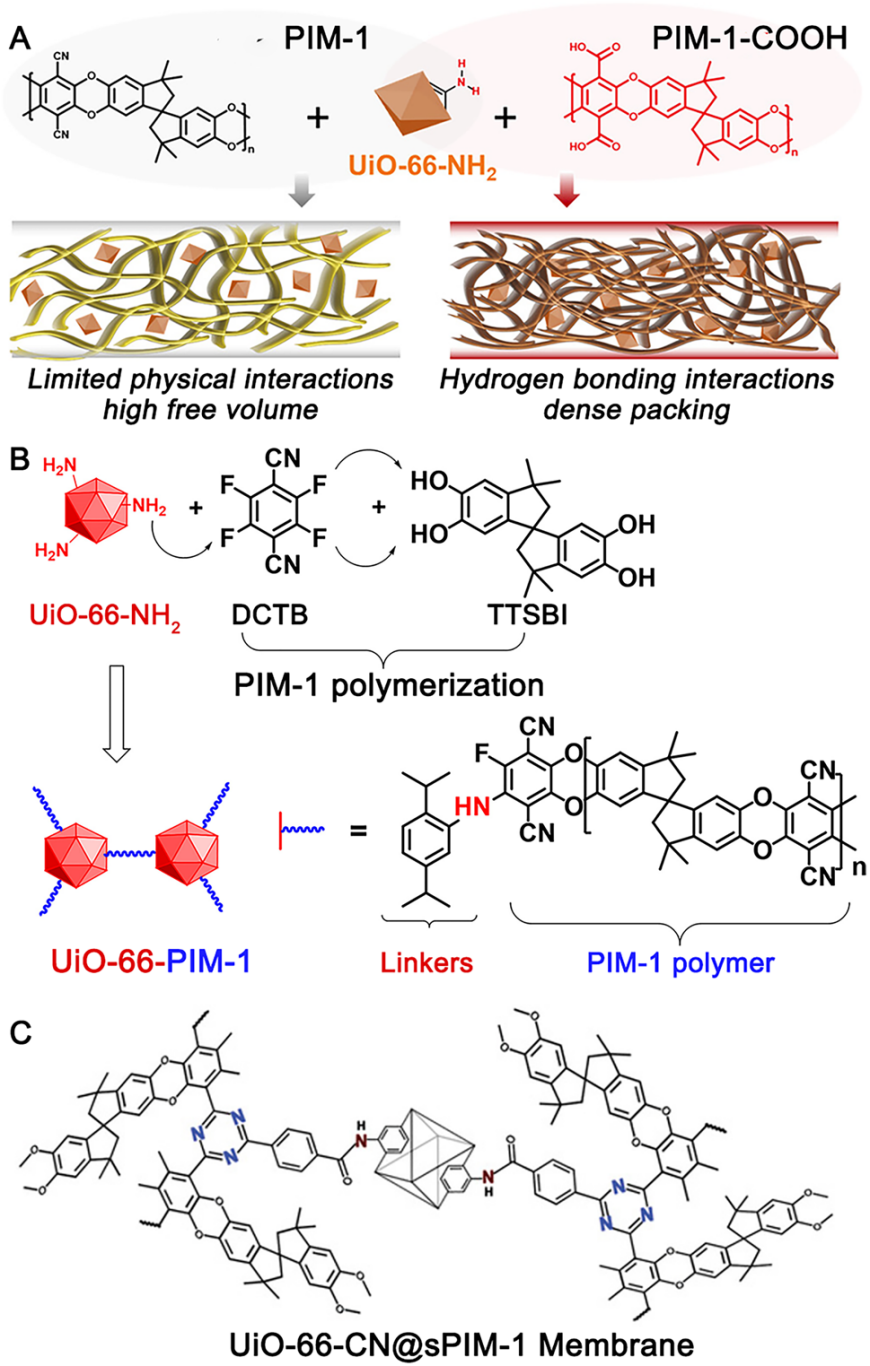
Improved MOF/polymer interaction by **A − B** hydrogen bonding and **C − D** covalent bonding. Adapted from Refs [15, 121, 146]. with permission

Besides, the chemical structure of MOFs can also be improved for a stronger interaction with PIM-1. Notably, construction of hydrogen bindings with relatively high bonding energy between (functionalised) MOFs and (modified) PIM-1 are demonstrated a feasible strategy to develop high-performance MMMs. Examples include the combination of ZIF-8 − OH with PIM-1 [108], UiO-66 with AO − PIM-1 [116], UiO-66 − NH_2_ with AO − PIM-1 [112], and UiO-66 − NH_2_ with PIM-1 − COOH [121, 145] (Fig. 7A). Beyond that, interweaving MOF nanocrystals and polymer through covalent bonding has been proven highly effective and has yielded the highest separation performance of PIM-1 MMM to date [15, 146]. Yu et al. [146] functionalised the surface of UiO-66 with − CN groups and then crosslinked the MOF with PIM-1 (Fig. 7B). This method created “connected paths for gas transport” with improved CO_2_ permeability and CO_2_/N_2_ selectivity. The covalent bonding was also created by *in-situ* chemical crosslinking between PIM-1 and UiO-66 − NH_2_ [15] (Fig. 7C). As a result, these studies developed MMMs with a CO_2_ permeability of > 12,000 barrer and a CO_2_/N_2_ selectivity of > 50.

Additionally, the introduction of an intermediate medium (such as polymer, ionic liquid, porous liquid) between the MOFs and PIM-1 polymers has also been proved effective for further enhancing the intimate bonding [123].

**Fig. 8:**
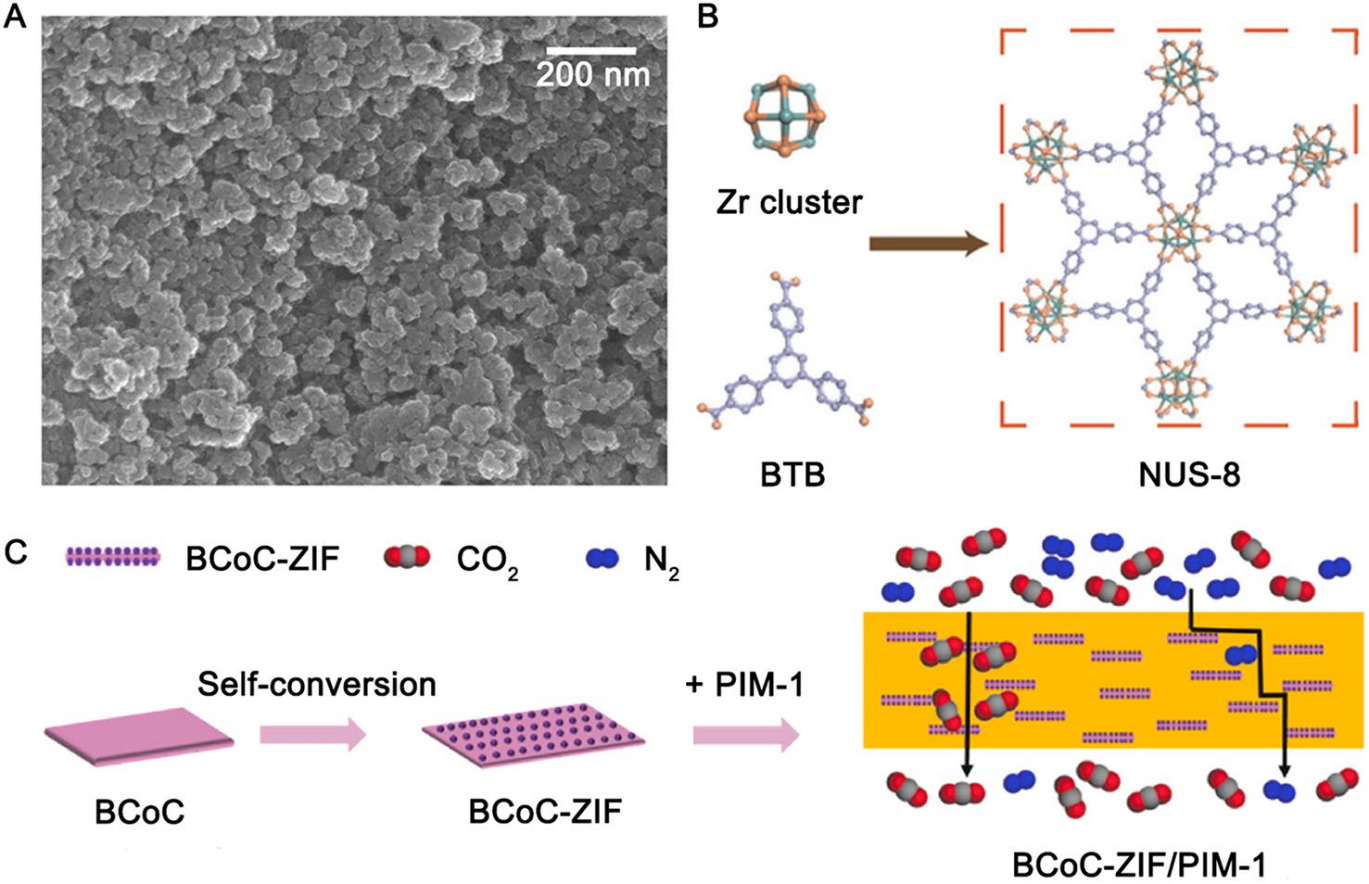
Regulating the morphology of MOFs (**A**: nanosized UiO-66 − NH_2_; **B**: 2D MOF: NUS-8; **C**: 2D MOF composites: BCoC-ZIF). Adapted from Refs [16, 113, 141]. with permission

The diversity of the MOFs morphology allows for optimisation of the MOF-polymer interaction. For the MOFs that have good compatibility with polymers, increasing the surface area of MOFs could maximise the MOF/polymer interfacial area, thus strengthen their interaction. Typical examples includes crystal engineering such as reducing crystallite size [109, 113, 135, 147] (Fig. 8A), defect engineering [117], and fabricating 2D MOFs (e.g., NUS-8 [16, 128–130] (Fig. 8B). It should be noted that the nanosizing of the MOFs has to be coupled with the good compatibility between PIM-1 polymer and MOFs to avoid the aggregation of the MOFs nanoparticles, as smaller fillers are more prone to aggregate due to their high surface energy [107].

Beyond the regular morphologies, some MOFs (e.g., ZIF-62 [142]) can be remoulded within a PIM-1 matrix using melt-quenching strategies, which involve the thermally induced glass transition at ~ 420 °C in argon. During this process, the MOFs retain the short-range inorganic–organic connectivity of their crystalline analogues, thereby retaining their capability for selective gas separation. Meanwhile, this strategy can diminish the non-selective voids between the MOF and the polymer. The reported MMMs exhibited a CO_2_ permeability of 5914 barrer and CO_2_/CH_4_ selectivity of 67, representing enhancements of 7.5% and 33% compared with the permeability and selectivity of the MMMs before the temperature treatment [142]. Additionally, Sun et al. [141] added 2D MOF composites (e.g., cobalt carbonate supported zeolitic imidazolate framework-67 (BCoC-ZIF) (Fig. 8C) into PIM-1 membranes. The synergistic effects of improved CO_2_ adsorption capacity, alignment of 2D morphology, and molecular sieving ability of the 2D MOF composites significantly enhanced the gas separation performance of PIM-1, which exceeded the 2019 upper bound for CO_2_/N_2_ [5].

Besides MOFs, the use of COFs (such as SNW-1 [148, 149], FCFT-1 [150], TpTta-COF [151]) can also enable MMMs to break the trade-off between the gas permeability and selectivity. Compared with MOFs, COFs nanosheets offer certain advantages, particularly in terms of compatibility with polymers due to their intrinsic 2D structure and fully organic nature.

### PIM-1 Blends

#### Blending with Other Polymers

Blending PIM-1 with other polymers has been recognised as one of the most cost- and time-effective routes as it combines the advantages of different materials into a new compound with unique and synergetic properties. Miscibility is one of the limitations for polymer blending, as less miscibility could lead to the separation of different phases with lower selectivity. For example, it was found that only small amounts of polyimide Matrimid® (< 10 wt%) is miscible with PIM-1 [152]. In comparison, cPIM-1 shows higher compatibility with various polymers (including of Matrimid® [153] and Torlon [154]) due to its capability of hydrogen bonding formation. Besides, post-crosslinking of the Matrimid®/PIM-1 membranes by diamines (e.g., ethylenediamine (EDA) and triethylenetetramine (TETA), et.al) were also proved to diminish the non-selective defect caused by the immiscibility between the polymers, while narrowing the fractional free volume and pore sizes. Consequently, these crosslinked membranes are more favour for separation of H_2_/CO_2_, with the selectivity increased from 0.8 to 9.6 [8].

Blending PIM-1 with polymers that have versatile CO_2_-affinity groups (ether oxygen groups in polyethylene glycol (PEG) [155, 156], sulfonated acid groups in sulfonated polyphenylenesulfone (sPPSU) [157], and polyether side groups in polyphosphazene (MEEP80 [158] and MEEP100 [159])), could enhance the membrane selectivity by promoting preferential sorption of CO_2_. Moreover, the interaction among guest polymers and between guest polymers/PIM-1 could also decrease the movability of PIM-1, thus narrow the pore-size distribution, and improve selectivity. However, due to the flexibility of the rubbery polymers, they can partially occupy the fractional free volume of the PIM-1, resulting in lower permeability, especially for those with bulky groups. Among the blended polymers, MEEP100 exhibited the less adverse effect on reducing CO_2_ permeability (~ 24%) after being blended with PIM-1, possibly due to the absence of bulk groups in its slim structure allowing effective intercalation into the pores of PIM-1 without decreasing the free volume significantly [159].

Additionally, a significant anti-plasticisation property was also shown by blending anti-plasticisation polymers into PIM-1, such as polyethyleneimine (PEI) [160] and sPPSU [157]. Due to the relatively firm interconnection among guest polymers and PIM-1 chains, the distortion of the packing of PIM-1 was prevented when facing highly condensable gases. It was reported that the blended polymer can withstand high pressure up to 30 bar without significant plasticisation. Furthermore, the increase in maximum tensile stress and Young’s modulus also indicated improved mechanical strength of the blended polymer [153, 158, 160].

#### Blends with Ionic Liquids

Ionic liquids (ILs), such as [APTMS][Ac] [161] and [C_2_mim][Tf_2_N] [162]), can also be blended into PIM-1. While ILs fill the free volume voids in the PIM-1 matrix, they increase the CO_2_ solubility coefficient due to the high solubility of CO_2_ in IL. Consequently, the selectivity of CO_2_/N_2_ and CO_2_/CH_4_ increased by 40% and 59%, respectively [161]. However, high loading of ILs led to agglomeration and caused phase separation in the copolymer matrix, thereby reducing membrane selectivity [162]. Furthermore, the loss of ILs at high pressure during membrane operation is hindering further development of this materials in membrane applications. One solution to this problem can be the immobilisation of ILs into other porous fillers such as MOFs, COFs, or polymeric hollow nanospheres [163].

## Comparison of the Various Modification Strategies

### Permeability and Selectivity for Gas Separation

The gas permeability and selectivity of 150 + PIM-1-based membranes that have been reported since 2005 can be found in Table S1**.** According to Table S1, the permeability and selectivity of PIM-1 (or modified PIM-1) in mixed gases are in general lower than those measured by single gases, due to the competition for sorption sites between the molecules in gas mixtures and the CO_2_-induced plasticisation. However, certain functionalities (e.g., TZ [30], MTZ [39], and amino-group [38]) can form strong interaction with CO_2_, thus facilitating the fast permeance of CO_2_, while hindering the transport of N_2_ or CH_4_ in gas mixtures. As a result, these membranes show a higher permeability and selectivity when separating gas mixtures (e.g., ~ 40 of TZ-PIM-1 and MTZ-PIM-1 when separating CO_2_/N_2_ mixtures).

**Fig. 9:**
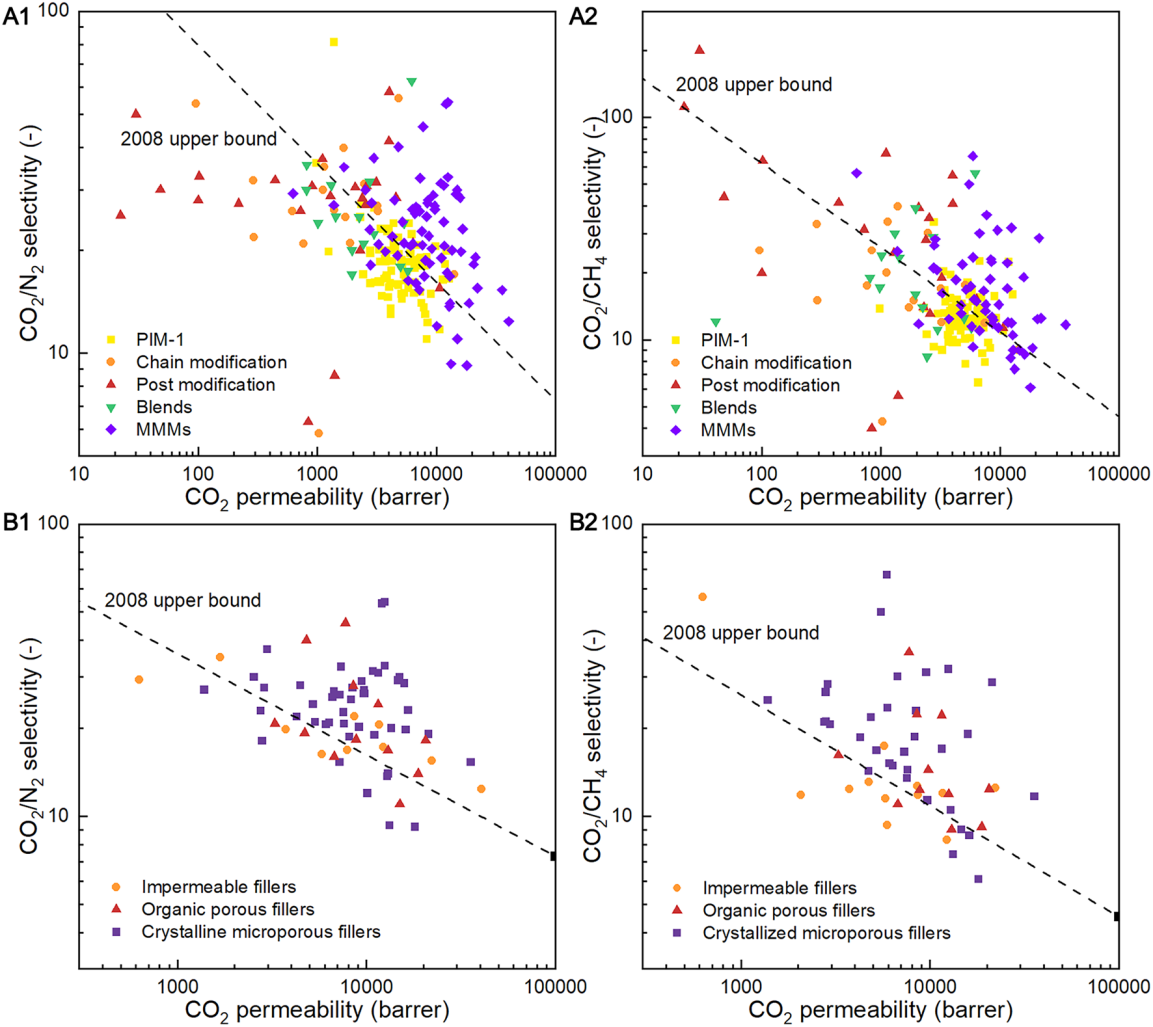
Upper bound plots for carbon capture from (**A1**, **B1**) CO_2_/N_2_ mixtures and (**A2, B2**) CO_2_/CH_4_ mixtures to show the effectiveness of the modified PIM-1 membranes by different methods

The selectivity vs permeability of relevant state-of-the-art PIM-1 membranes are plotted in Fig. 9 A1, A2. Here, single-gas permeability and ideal selectivity are used in most cases, while for the membranes lacking values of single-gas separation performance, mixed gas permeability and selectivity was used instead, as according to statistics in Table S1, the difference between the two is generally less than 20%. Additionally, for the membranes which have significantly higher (> 50%) separation performance in gas mixtures, we also used their mixed-gas permeability and selectivity for plotting and comparison. As shown in Fig. 9A1, A2, the permeability of pristine PIM-1 is approximately 5500 barrer in average, with the selectivity for CO_2_/N_2_ and CO_2_/CH_4_ at around 18 and 13 in average, respectively, laying on the 2008 Robeson’s upper bound or very close to it [3]. Therefore, the 2008 Robeson’s upper bound was utilised to evaluate the effect of the 4 types of modification strategies.

Notably in Fig. 9 A1, A2, a large portion of PIM-1 membranes modified by chains modification and post-modification of PIM-1 (89%) exhibits lower permeability compared with average permeability of pristine PIM-1 membranes. This is because most of these strategies involve a decrease in pore sizes due to: i) the stronger attraction between polymer chains caused by the introducing of secondary interaction (e.g., by introducing polar functional groups); ii) the shrinkage of the porous structure under certain trigger (such as thermal treatment, oxidation, UV irradiation, and crosslinking); iii) pore blocking (e.g., by heterogeneous materials deposition). It is worth noting that a few modified PIM-1 membranes prepared by post-modification strategy did not experience large permeability reduction as: i) they were solidified to membranes before modification (therefore the movement of polymer chains were more restricted), or ii) the modification was not carried out throughout the whole cross section of the membrane (e.g., surface treatment). However, a small reduction of permeability was still observed in most of these modified membranes, according to the meta-analysis (Fig. 9 A1, A2).

On the other hand, a great percentage of the membranes modified by chains modification and post-modification shows a higher membrane selectivity compared to the averaged value of pristine PIM-1 membranes (e.g., both 88% for CO_2_/N_2_ selectivity), which is attributed to highly rigidification of the polymer chains due to the strengthened polymer interaction, higher densification of chain packing (which reduce the movability of polymer chains), and rigidification caused by the interaction with heterogeneous materials. The high rigidification of polymer chains would inhibit the transient changes in pore shape/size caused by molecular thermal motion of polymer chains, thus provide a narrow pore size distribution and strict size-sieving. As a result, membranes modified by both strategies exhibit an improved selectivity for CO_2_/N_2_ and CO_2_/CH_4_, up to 58.1 [52] and 200 [65] respectively. Although only 42% and 41% of the membranes modified by these two strategies exceed the CO_2_/N_2_ upper bound, some of them are favourable for separating smaller gas molecules such as H_2_ due to the lower pore sizes.

PIM-1 membranes modified by blending with other polymers (mostly rubbery polymers) also exhibit in general lower permeability, due to the smaller pore sizes caused by the insertion of rubbery polymers into the fractional free-volume of super glassy PIM-1. However, the selectivity shows an increase after blending, owing to the enhanced CO_2_ selectivity transportation by the CO_2_-affinity sites on guest components, and reduced PIM-1 movability due to the contraction of inter-connected guest polymer chains and interaction between PIM-1 and rubbery polymer chains. Probability due to the broader pore-size distribution of the rubbery polymers, only 19% and 33% of PIM-1 blends are beyond the 2008 upper bound for CO_2_/N_2_ and CO_2_/CH_4_, respectively.

In contrast to the other modification strategies, the addition of fillers shows a significant increase in permeability in a great number of cases. At the same time, PIM-1 MMMs show an improved selectivity for CO_2_ preferential separation. This is attributed to the additional mass transfer path introduced by the intrinsic inner pore structure of porous fillers (including crystalline microporous fillers and organic porous fillers), and the CO_2_-affinity sites on the fillers that boost the selective transportation of CO_2_. Besides, the improved rigidity of the PIM-1 chains in the vicinity of the fillers also contributes to narrowing the pore-size distribution, which is favourable for improving the membrane selective. As a consequence, the strategy of filler incorporation resulted in the largest the proportion of membranes surpassing the 2008 upper bound for CO_2_/N_2_ and CO_2_/CH_4_ (at 76% and 75%, respectively) among the four modification strategies.

Furthermore, the effect of different types of fillers was further analysed, as shown in Fig. 9 B1, B2. Notably, they crystalline microporous fillers shows the most effectiveness among the heterogeneous fillers, with 80% and 85% of the MMMs surpass the 2008 upper bounds for CO_2_/N_2_ and CO_2_/CH_4_ gas pairs, respectively. Besides, 67% and 73% of the MMMs containing organic porous fillers surpass the upper bound for CO_2_/N_2_ and CO_2_/CH_4_, respectively.

This indicates that the porous structures are highly preferable for improving the separation performance as fillers. This could be due to the additional molecular sieving effect that can be provided by crystalline microporous fillers; besides, the CO_2_-affinity sites in the porous fillers can also boost preferential transportation of CO_2_. On the contrary, only 64% and 50% of membranes with impermeable fillers lie beyond the CO_2_/N_2_ and CO_2_/CH_4_ upper bounds, respectively.

### Anti-Physical Aging

**Fig. 10:**
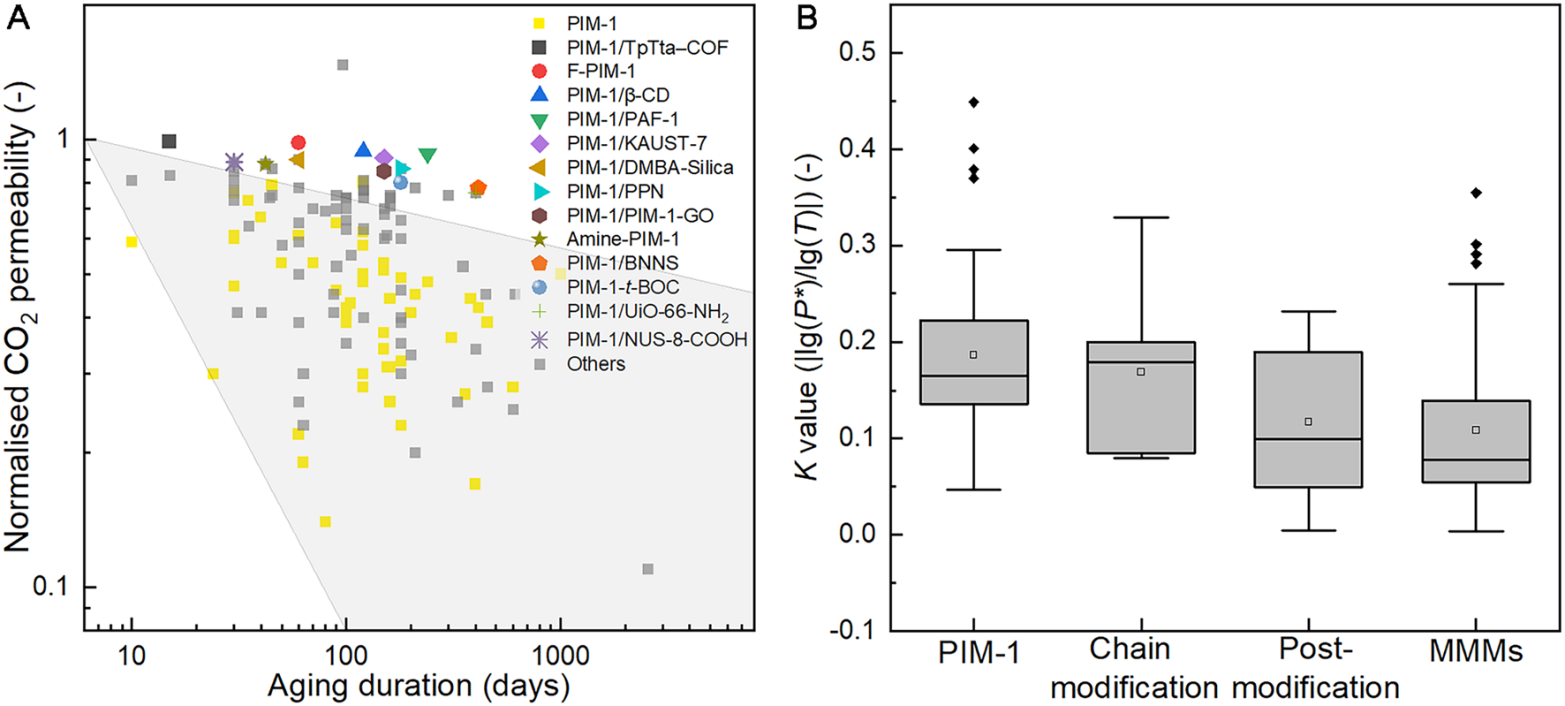
Physical aging of PIM-1 based membranes. (**A**: Statistics of the normalised CO_2_ permeability vs. aging days of the PIM-1 based membranes. The shaded area represents the performance of pristine PIM-1 (for eyes guidance only); **B**: Comparison between different modification strategies)

Physical aging in glassy polymers involves the densification of polymer chain packing and reduction in free volume due to the gradual transition to the equilibrium state. Hence, a fundamental approach to alleviate physical aging is to: (i) introduce porous structures in membranes which provide permanent mass transfer channels, and (ii) hinder the free movement of PIM-1 chains. The reported aging performance of self-standing PIM-1-based membranes (thickness > 20 µm) is summarised in Table S2 and Fig. 10A, and several examples with the highest anti-aging performance are highlighted in Fig. 10A.

Accordingly, only 40% and 20% of the initial CO_2_ permeability is maintained over a period of 100 and 400 days, respectively. Little work has reported the anti-aging performance of membranes made by PIM-1 blends. Notably, among current reports about PIM-1 membranes modified by chain modification and post-modification, only a few strategies, such as fluorination [7], amination [164], and tert-butoxycarbonyl (− *t*BOC) [47] functionalisation have shown to be effective for reducing the aging effect (Fig. 10A), however, this could be due to the already low initial free volume fraction after the modification, indicated by their consistently low membrane permeability (CO_2_ permeability < 100 barrer). On the contrary, in most cases, no significant improvement in anti-aging performance was observed, and in some cases it even worsened. For instance, the CO_2_ permeability of the thermally-crosslinked PIM-1 after 455 days was 28% compared with the value for the fresh membranes [32]. Similarly, the CO_2_ permeability of both carboxylated [33] and sulfonated PIM-1 [48] was 26% after 330 and 60 days, respectively. These findings suggest that the intermolecular bonding among PIM-1 chains (ligand bonding, hydrogen bonding and covalent bonding) might not impede physical aging of PIM-1. Conversely, the enhanced intermolecular bonding seems to induce polymer chain densification resulting in decreased permeability over time.

In comparison, the introduction of heterogeneous fillers exhibits high effectiveness in anti-aging. As shown in Fig. 10A, among the 15 of the PIM-1 membranes with the highest anti-aging performance 12 of them belongs to the MMMs.

To further compare the effectiveness of different modification strategies, we calculated the ratio between the absolute value of the logarithmic aging time and the logarithmic mormalised membrane permeability, defined as the *K* value (|lg(P*)/lg(*T*)|). A higher *K* value indicates a faster aging rate. As shown in Fig. 10B, the median *K* value of the MMMs is 0.08, which is significantly lower than that of pure PIM-1 (0.17), suggesting that MMMs have a slower aging rate. In comparison, the effectiveness of chain modification and post-modification for reducing physical aging is not statically significant, based on the Dunn’s Test Pairwise Comparisons.

The prominent anti-aging effect of heterogeneous fillers indicate the good effectiveness of the interfacial interaction between the fillers and the polymer. Due to the interaction between the fillers and PIM-1 chains in their vicinity, the fillers can rigidify the PIM-1 in its vicinity, i.e., reducing the mobility of the PIM-1 chains. Additionally, some fillers that have permanent porous structure also alleviated the physical aging.

Notably, most the fillers in MMMs that show high anti-aging performance exhibit a relatively strong interfacial interaction by covalent and hydrogen bonds, which have higher high bonding energies then van der Waals forces, ligand bonds. Notable examples include PIM-1 MMMs with covalently crosslinked β-CD [93], PPN1 [86], GO [77, 81], POSS [100], and UiO-66 − NH_2_ [15], as well as PIM-1/NUS-8 − COOH [128], PIM-1/DMBA-silica [76], and PIM-1/KAUST-7 [140] linked by hydrogen bonding (Fig. 10A). Additionally, “physical crosslinking” is known to be highly effective in retarding physical aging [90, 95]. For example, membranes containing PAF-1 exhibited only 7% lower CO_2_ permeability after 240 days, making it one of the most effective strategies for preventing physical aging [90].

Additionally, more than half of the MMMs showing high anti-aging performance are composed of nanofillers and 2D fillers, demonstrating the positive effect of high specific surface area, which could enable larger interaction areas at the same filler loading. Examples include PAF-1 [90], PIM-1-GO [81], NUS-8 − COOH [128], BNNS [165], and TpTta-COF [151] (Fig. 10A).

Moreover, porous fillers (including organic porous fillers and crystalline microporous fillers) are also present in a large number of MMMs with high anti-aging performance, indicating the effectiveness of additional permanent mass transfer path in reserving a high permeability of PIM-1 membranes after aging. Example including β-CD [93], PAF-1 [90], PPN1 [86], UiO-66 − NH_2_ [15], TpTta-COF [151], KAUST-7 [140] (Fig. 10A).

In conclusion, compared with enhancing the intermolecular interaction among PIM-1 chains, the introduction of heterogeneous fillers for creating interfacial interaction is more effectiveness in preventing the physical aging of PIM-1 membranes. In particular, the high interfacial forces and high interfacial areas is crucial in achieving a high anti-aging performance. Besides, the presence of the permanent porous structure in fillers are favourable for maintain a high permeability after aging.

### Anti-Plasticisation

At high pressures, condensable gases such as CO_2_ causes the polymer chain matrix to swell, resulting in a change in the shape or size of the cavities, known as plasticisation. Typical, increased CO_2_ permeability under increased pressure is an indication that plasticisation is occurring. However, as for PIM-1 with high CO_2_ adsorption, the CO_2_ permeability is not only related to the diffusion of molecules between the polymer chains, but also positively related to the solubility of CO_2_ in the membrane, which decreases with increasing pressure. Due to these two reversing effects, the permeability of PIM-1 generally exhibits a decrease with pressure at low pressures and an increase with pressure at high pressures. In this case, the pressure at which the trend changes, i.e. the plasticisation point, is used to describe the membrane’s resistance to plasticisation.

Currently, literature reports on the plasticisation points of PIM-1 membranes are limited. Based on the available literature, the plasticisation point of PIM-1 is estimated to be around 7–15 bar [29, 38, 69, 124, 140, 166]. Among the various strategies of PIM-1 modification, blending with suitable non-plasticising polymers can alleviate the plasticisation to a certain extent. For example, the plasticisation points of PIM-1 blended with Torlon [154] and PEI [160] are higher than 30 bar and 45 bar, respectively. It could be due to that the intermolecular interactions among the guest fillers inhibit the movement of PIM-1.

Regarding to the strategies of chain modification and post-modification, it was found that secondary interactions introduced by modification shows an inconsistent affect in anti-plasticisation. While PIM-1 functionalised with –NH_2_ [38] and –AO [29] inhibited an improvement in anti-plasticisation (plasticisation points > 26 bar and > 20 bar, respectively), –COOH functionalisation exacerbates plasticisation, reducing the plasticisation point to 5.2 bar [33]. This could be due to that, with increasing pressure and CO_2_ concentration, in some cases, the less stable intermolecular linkages in the polymer backbone (ligand/hydrogen bonds) may be disrupted, and more readily bound to CO_2_, thus inducing the plasticisation of PIM-1 membranes in CO_2_ atmosphere. Notably, crosslinked PIM-1 membranes were reported to exhibit better resistance to plasticisation than their counterparts due to their more stable covalent linkages. For instance, crosslinked PIM-1 via the assistance of azide [51], and induced by ester [54], decarboxylation (DC) [53] or bromoalkylated (–BM) groups [55] all have shown plasticisation resistance for operating pressures below 35 bar. The improved resistance may be attributed to the strong covalent bonds preventing PIM-1 chains from swelling.

Another indicator of the degree of plasticisation is the increased permeability of less condensable gases in the mixture (e.g., CH_4_). When polymers are plasticised, matrix expand/distort due to increased polymer fragment movement, which would greatly increase the diffusion of all gases, including less permeable gases like CH_4_. As a consequence, the PIM-1 membranes show a reduction in selectivity in mixed-gas separation.

**Fig. 11:**
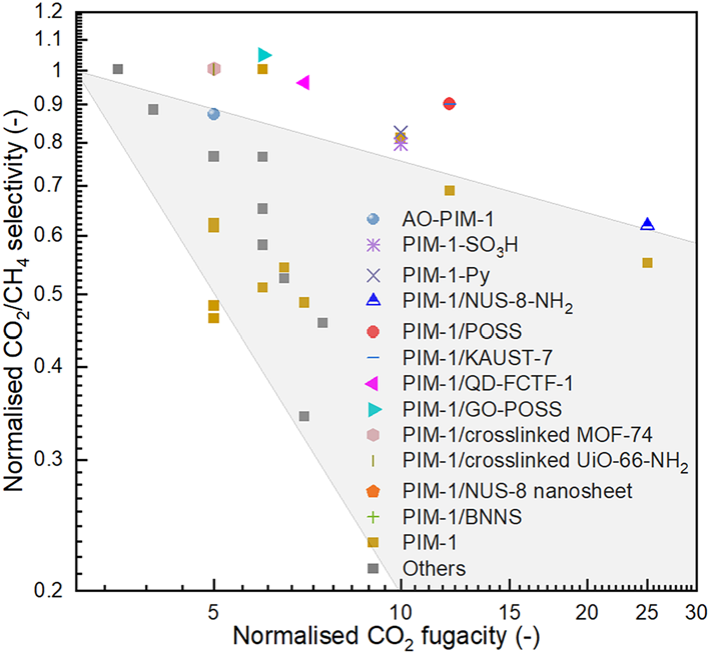
Normalised CO_2_/CH_4_ selectivity as a function of the normalised CO_2_ fugacity, the shaded area represents the performance of pristine PIM-1 (for guiding eyes only)

Based on this indicator, the normalised CO_2_/CH_4_ selectivity as a functional of normalised CO_2_ fugacity was plotted and compared, as shown in Fig. 11. It was found that AO-PIM-1 was proved to have good ant plasticisation. Although have a plasticisation point similar to PIM-1 (~ 10 bar), the CO_2_/CH_4_ selectivity dropped by 13% as the CO_2_ fugacity increased from 2 to 10 bar, while for the PIM-1, the selectivity only dropped by 39% [29]. Additionally, a variety of MMMs with fillers including NUS-8 [16], NUS-8 − NH_2_ [130], POSS [97], KAUST-7 [140], QD-FCTF-1 [167], GO-POSS [80], BNNS [165], crosslinked MOF-74 [124], and UiO-66 − NH_2_ [15] shows superiority in the anti-plasticisation (Fig. 11). This could be due to the effect of the interfacial interaction induced PIM-1 rigidification, and in some cases, the addition of non-plasticised porous fillers.

## PIM-1 Thin Film Composite Membranes

Membranes are commonly studied as free-standing films with thicknesses exceeding 20 μm (typically 40–100 μm) to evaluate their intrinsic properties. However, for practical applications, thin selective layers (< 5 μm supported onto mechanically stable and highly porous substrates) should be used. The gas properties observed in thicker free-standing membranes often cannot be extrapolated to thinner TFC membranes. Due to the broad cavity size distribution of PIM-1, the membranes exhibit a trade-off between selectivity and permeance as thickness decreases. Additionally, thinner membranes are more sensitive to the presence of structural defects. Consequently, PIM-1 TFCs often display lower selectivity compared to their thicker counterparts. Besides, thinner PIM-1 membranes would also suffer from higher densification, which could also contribute to the decrease of permeability [168]. The reduced thickness of the active layer also limits the size and morphology of fillers that can be incorporated. Additionally, the fast movability of PIM-1 chains in the thinner membranes also accelerates physical aging, leading to a more rapid decrease of permeance over time.

Herein, we comprehensively reviewed the strategies for fabricating PIM-1 thin film composite membranes and their corresponding separation performance. Besides, several key challenges in thin film membranes of PIM-1 and strategies for coping with them was critically reviewed, including PIM-1 penetration (into the substrates), filler applicability, and more rapid physical aging. This section is for providing insights into the fabrication of high-performance PIM-1 TFCs that are better suited for industrial applications.

### Fabrication of PIM-1 Thin Film Composite Membranes

Differ from free-standing PIM-1 membranes which are produced by casting in petri dishes, the TFC membranes can be fabricated using scalable methods such as spray-coating, dip-coating, or roller coating. Among them, spin-coating and dip-coating are the two most commonly used strategies for fabricating PIM-1 thin film composite membranes. Dating back to 2012, Harms et al. [169] first prepared PIM-1 with a thickness of 200–400 nm by spin coating on a Si substrate; similarly, Ogieglo et al. [170] also prepared 12 nm PIM-1 film on SiO_2_/Si wafers. However, the PIM-1 on a dense substrate prepared by spin coating is generally for *in-situ* analysis for the physical property including aging, swelling, and change of refractive index.

Prepared by spin-coating, Gorgojo et al. [171] transferred the spin-coated PIM-1 (35–660 nm) onto polyacrylonitrile (PAN) and alumina (AAO) support to fabricate PIM-1 TFCs. It was demonstrated that a 35 nm thick PIM-1 membrane possesses a Young's modulus of 222 MPa, and the TFC shows excellent stability under hydraulic pressures of up to 15 bar in organic solvent nanofiltration (OSN). After that, spin-coating was also used for directly coating PIM-1 onto porous substrates (e.g., AAO) [168].

Another strategy to fabricate PIM-1 TFCs is dip-coating. Between 2012 and 2015, Fritsch et al. [172] and Scholes et al. [173, 174] successively prepared PIM-1-based TFC membranes on PAN and PP substrates by dip-coating for applications such as nanofiltration of organic solvents and membrane contactors. The PIM-1 TFC (with PEI additive and thermal crosslinked) exhibiting 30 times higher *n*-heptane permeance than industrial Starmem™ 240 membranes. Dip-coated derivatives for the manufacture of PIM-1 TFCs, including roller-coating and kiss-coating, have also been developed due to their continuity and higher potential for industrial manufacturing [175].

Several other strategies have been developed for PIM-1 TFC fabrication, including rod casting and electrospray, which offer the advantage of continuous production. However, due to the low viscosity of the PIM-1 coating solution, there is a higher likelihood of it penetrating into the pores of the substrate during the PIM-1 coating solution squeezing by the coating rod, thus increasing mass transfer resistance [176]. Additional, during electrospray, the rapid evaporation of the solvent from the spray may lead to a dense build-up of polymer chains [177]. Therefore, both of these methods require further optimisation.

Apart from the configuration of thin film composite membranes, PIM-1 has also been spun to produce integrally skinned, asymmetric hollow fibre membranes [178], where some of the PIM-1 forms the separator layer and the rest forms the porous support layer. However, this structure appears to be inferior to TFC because it seems unnecessary to use as much PIM-1 material to make the porous support layer, and the porosity of these PIM-1-based porous substrates is lower than that of most commercially available porous substrates.

The selectivity and permeance of PIM-1 TFC membranes to date is summarised in Table 1. The average permeance of PIM-1 TFC membranes (with an active layer thickness of < 5 μm) is approximately 4000 GPU (GPU = barrer/μm). In comparison, the average permeability of PIM-1 is 5500 barrer; thus, a free-standing membrane with a thickness of 40 μm would exhibit a permeance of 100–200 GPU. This indicates that the permeance of the TFC membranes is one order of magnitude higher than that of the free-standing membranes.

**Table 1:** Gas separation performance of PIM-1 TFC/TFN membranes (at 20 − 40 °C and 1 − 4 bar)

Membranes	Fabrication	Substrate	Thickness (µm)	Gas permeance (GPU)			Selectivity		Refs
				CO_2_	N_2_	CH_4_	CO_2_/N_2_	CO_2_/ CH_4_	
PIM-1	Dip-coating	PAN	0.75	7147	253^*^	464^*^	28.2^*^	15.4^*^	[179]
MWCNT/PIM-1	Dip-coating	PAN	0.75	8173	500	1008	16.3	8.1	[179]
f-MWCNTs/PIM-1	Dip-coating	PAN	0.75	9453	287^*^	909^*^	33.0^*^	10.4^*^	[179]
PIM-1/OAPS	Spin-coating	AAO	2.5	2138	110	201	19.4	10.6	[100]
PIM-1/OAPS	Spin-coating	AAO	0.2	13,585	707	1406	19.2	9.7	[100]
PIM-1	Spin-coating	AAO	2.5	2403	154	275	15.6	8.7	[100]
PIM-1	Spin-coating	AAO	0.2	16,917	1125	2058	15.0	8.2	[100]
PIM-1 HFM		–	2.8	540		22		25	[178]
PIM-1	Spin-coating	AAO	0.61	1765				4.6	[180]
PIM-1 PIM-1/CuBDC NS	Spin-coating	AAO	0.66	407.3				15.6	[180]
PIM-1	Spin-coating	AAO		3600	390		9.2		[113]
PIM-1/UiO-66–NH_2_	Spin-coating	AAO		1740	72		24.1		[113]
PIM-1	Dip-coating	UV-DHPA		1874	156		12.0		[181]
Metallic ion-crosslinked PIM-1 TFC	Dip-coating	UV-DHPA		1058	45		23.5		[181]
PIM-1	Dip-coating	PAN	2.08	3331	234		14.2		[182]
PIM-1/HCP	Dip-coating	PAN	6.32	21,627	4199		5.2		[182]
PIM-1/C-HCP	Dip-coating	PAN	8.05	27,530	4197		6.6		[182]
PIM-1/PTMSP	Kiss-coating	MFFK-1	0.29	8010	224			35.8	[183]
PIM-1	Spin-coating	PDMS@aMOF/PAN	0.85	4320			19.0		[184]
PIM-1/Ni-MOF-74	Spin-coating	PDMS@aMOF/PAN	0.87	5018			31.0		[184]
PIM-1/UiO-66–NH_2_	Spin-coating	PDMS@aMOF/PAN	0.86	7460			26.0		[184]
PIM-1/nanoporous graphene	Spin-coating	–	0.4	870			25.4	20.4	[185]
PIM-1	Electrospray	PAN	2.75	156			12		[177]
PIM-1/HKUST-1	Electrospray	PAN	2.75	696			6.4		[177]
PIM-1/MOF-74	Dip-coating	PAN	0.2	1939	112	156	17.1	12.2	[186]
PIM-1, #3b, network content 7.8 wt%	Roller-coating	PAN	~ 2	5985	303		19.9		[45]
PIM-1. #1, network content 0.8 wt%	Roller-coating	PAN	1.92	4678	419		11.2		[46]
PIM-1. #3, network content 85.3 wt%	Roller-coating	PAN	2	1514	804		1.9		[46]
Blended 20%PIM-1#3 in PIM-1#1	Roller-coating	PAN	2	5910	357		16.6		[46]
PIM-1	Roller-coating	PAN	2–4	2778		441		6.3	[75]
PIM-1/SN	Roller-coating	PAN	2–4	2864		461		6.2	[75]
PIM-1/S-SN	Roller-coating	PAN	2–4	2755		423		6.5	[75]
PIM-1	Roller-coating	PAN	2.895	1583		190		9.2	[79]
PIM-1/rHGO–TAPA	Roller-coating	PAN	1.572	1050		79		13.3	[79]
PIM-1	Spin-coating	PDMS/PAN	0.89	988		225		4.4	[68]
PIM-1/PAF	Spin-coating	PDMS/PAN	0.89	1699		419		4.1	[68]
PIM-1	Roller-coating	PAN	0.78	4107					[108]
PIM-1/ZIF-8	Roller-coating	PAN	0.78	7052					[108]
PIM-1/HZIF-8-TA	Roller-coating	PAN	0.78	5788					[108]
B-cPIM-1	Roller-coating	PAN	1.39	3200	58	83	64.0	45.0	[187]
B-PIM-1	Roller-coating	PAN	3.61	3100	170	300	19.0	11.0	[187]
D-PIM-1	Roller-coating	PAN	3.72	6200	430	830	14.0	7.5	[187]
D-cPIM-1	Roller-coating	PAN	0.63	7700	140	210	56.0	37.0	[187]
PIM-1	Roller-coating	PAN	2.12	4599	219	424	21.0	13.0	[145]
PIM-1/UiO-66–NH_2_/cPIM-1	Roller-coating	PAN	2.05	2664	87	143	29.2	19.0	[145]
PIM-1	Rod-coating	PDMS/PSF	2.5	755	32		23.6		[122]
PIM-1/UiO-66–(CF_3_)_2_	Rod-coating	PDMS/PSF	2.5	1255	33		38.0		[122]

Asterisks indicate values extracted from figures in literature

### Challenges in PIM-1 Thin Film Membranes

#### PIM-1 Penetration

To maximise the permeance of the PIM-1 TFC, it is preferred to have a thinner active layer coated with a diluted PIM-1 coating solution to reduce mass transfer resistance. However, the PIM-1 solution has relatively low viscosity compared with other polymers such as polydimethylsiloxane (PDMS) and poly(1-trimethylsilyl-1-propyne) (PTMSP). Therefore, inevitably, the coating solution is prone to penetrate into the pores of the substrate. For example, TEM characterisation of the 4% w/v B-cPIM-1–73% TFC revealed that the active layer is 1.39 μm, with approximately 0.8 μm of the polymer penetrated into the underlying PAN support [187], leading to two issues:

(i) The penetrated PIM-1 in the substrate pores would lead to additional mass transfer resistance, thus decreasing the membrane permeance. In particular, thinner TFCs need to be coated with diluted PIM-1 solutions, which have lower viscosity, resulting in more significant PIM-1 penetration. Consequently, TFC permeability is not inversely related to the thickness of the active layer.
(ii) Penetration of the PIM-1 solution into the porous base membrane leads to inhomogeneity of the active layer, which promotes defects formation and reduces membrane selectivity.

It was found that the thin film membranes fabricated on a porous substrate pre-coated with a permeable intermediate layer, which was often referred to as a gutter layer, can prevent the issues above. To fabricate TFCs with ultra-thin PIM-1 active layers, gutter layers including PTMSP [183] and PDMS [68, 122, 184] were explored. The gutter layer provides a smooth and dense surface, which serves as an excellent platform for supporting PIM-1. For example, using the highly permeable PTMSP, the PIM-1/PTMSP TFC with a thickness of 290 nm has was fabricated, showing the CO_2_ permeability of 8010 GPU with a CO_2_/N_2_ selectivity of 35.8 [183]. However, both PTMSP and PIM-1 ages rapidly, and hence the PIM-1/PTMSP TFC experienced a significant drop in CO_2_ permeance by 97.4% after 98 days.

PDMS, on the other hand, is a rubbery polymer being less prone to physical aging and is therefore being more commonly employed as the gutter layer. However, PDMS has lower permeability, resulting in relatively low TFC permeance (< 1700 GPU). Consequently, efforts have been made to increase TFC permeability to 4000–7000 GPU by introducing MOFs into PDMS and reducing the mass transfer resistance of the PDMS gutter layer.

In addition to the methods using the gutter layer, the pre-treatment of the porous substrate using solvents such as chloroform, methanol, and ethanol (to fill the pores in the substrate) was also effective to prevent the penetration of PIM-1 during the coating process. For example, the PIM-1 TFCs on polyvinylidene fluoride (PVDF) impregnated by methanol, ethanol, and water exhibited the improved selectivity/selectivity in pervaporation [188, 189]. However, this method is seldom applied in fabricating PIM-1 TFCs for gas separation, as compared with liquid separation, gas separation is more sensitive to the structural changes of PIM-1 caused by the penetration/residue of the solvent into PIM-1 matrix.

#### Filler Applicability

Many studies of MMMs utilised fillers consisting of relatively large particles (> 1 µm), which are not appropriate for preparing defect-free PIM-1 thin film nanocomposite membranes (TFNs) because the use of relatively large fillers could bridge the entire thickness of the very thin films. Accordingly, in fabrication of TFN membranes, the fillers need be in the nanometre range.

Depending on length scale of the sizes of filler particles, the fillers for preparing TFN membranes can be categorised as below:

(i) Zero-dimension (0D) fillers: all three dimensions have sizes of the nanoscale (less than 100 nm). Examples include nanosized MOF crystals such as UiO-66-NH_2_ (10 − 30 nm) [145, 184], HKUST-1 (30 − 50 nm) [177] and Ni-MOF-74 (20 − 30 nm) [184], and HCP (55 nm) [182].
(ii) One-dimension (1D) fillers: two dimensions on the nanoscale such as carbon nanotubes [179].
(iii) Two-dimension (2D) fillers: one dimension on the nanoscale. Examples include GO [185], CuBDC MOF nanosheets (NS) [180], and silica nanosheets (SN) [75].

**Fig. 12:**
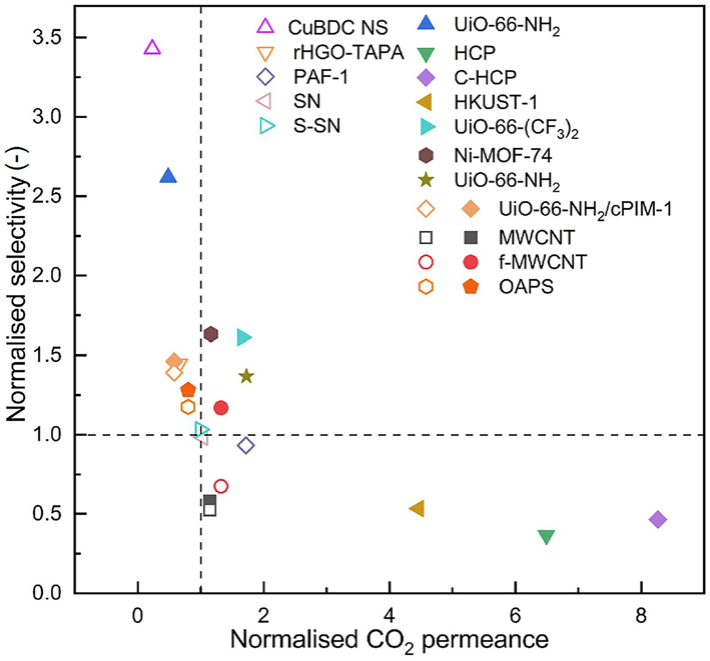
Effect of fillers on the separation performance for CO_2_/N_2_ (closed symbols) and CO_2_/CH_4_ (open symbols) of the PIM-1 TFN membranes

The effect of different fillers on the separation performance of the PIM-1 TFN membranes is shown in Fig. 12. Similar to the thick MMMs membranes, organic porous fillers were effective in improving membrane permeability by up to 8.26-fold over PIM-1 TFC membranes, as the highly porous fillers provide additional mass transfer paths with low resistance [182]. MOFs were more effective in improving membrane selectivity and could break the trade-off between membrane permeability and selectivity due to their rigid crystallised framework structure, which endows relatively precise sieving effect and abundant CO_2_-affinity sorption sites. For example, Ni-MOF-74 [184], UiO-66 − NH_2_ [184], and UiO-66 − (CF_3_)_2_ [122]) are able to break the trade-off between membrane permeability and selectivity as illustrated in Fig. 12.

#### Physical Aging

**Fig. 13:**
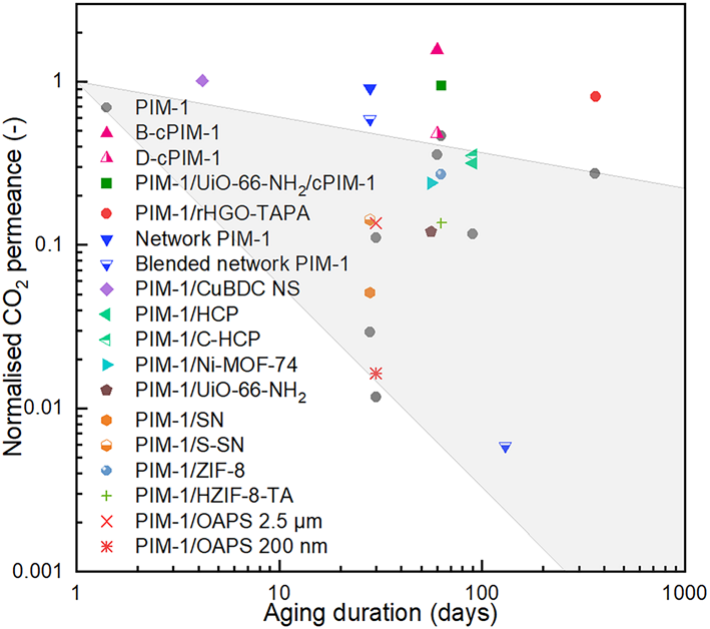
Performance of physical aging of the PIM-1 TFC/TFN membranes (normalised CO_2_ permeance vs. aging duration; shaded area shows the physical aging of pristine PIM-1 for eyes guidance)

Physical aging was found to be more significant in thinner selective layers. For example, Kinoshita et al. [100], reported that, PIM-1 TFC membranes with an active layer thickness of 0.2 μm exhibited a normalised CO_2_ permeance of only 0.01 after 30 days. This is an order of magnitude lower than those of ~ 70 μm-thick membranes which showed a normalised CO_2_ permeance of 0.8 for the same aging period. Figure 13 and Table S3 show the physical aging of PIM-1-based thin film membranes (with active layer thicknesses ranging from 0.2 to 8 μm). After 100 days of aging, the normalised CO_2_ permeability of the thin films decreased to 0.01 − 0.5, significantly lower than the normalised CO_2_ permeability of the thick membranes after the same period (0.22 − 0.9) (Fig. 10A).

It should be noted that the anti-aging mechanisms by enhancing the interactions between the polymer chains may be different in thin and thick films. For example, the carboxylation of PIM-1 cannot improve the anti-aging performance of the thick films (> 30 μm in estimation) [33], however, in thin films with an active layer less than 2 μm, carboxylated PIM-1 shows great anti-aging performance, with no aging was observed within 60 days [187]. Hence, this aspect can be further studied, additionally, the modification strategies of PIM-1 should also be investigated in thin film membranes to validate its effectiveness.

By modifying the topology of PIM-1, the PIM-1 with a high content of network and colloids structure also shows high anti-aging ability of TFC, with a normalised CO_2_ permeance of 0.91 in 28 days, although being less selective than pristine PIM-1 [46]. Mixing the network PIM-1 with pristine PIM-1 lead to a more balanced separation performance and anti-aging properties, resulting in a normalised CO_2_ permeance of 0.59 in 28 days. However, the normalised permeance of the membrane showed a rapid decrease after 130 days to 0.01, probably due to the lack of structural content (network and colloids) [46]. This also indicates the challenge of preventing the physical of PIM-1 in the longer term.

Filler addition also favours the rigidification of PIM-1 chains around fillers in TFN, which could retard physical aging of the membranes [75, 79, 108, 182, 184]. Similar to MMMs, high fraction of rigidified PIM-1 and good interfacial interaction is necessary for obtaining a good anti-aging property. Therefore, the 2D filler rHGO-TAPA which have both large filler/polymer interfacial area and good interfacial interaction showed the highest normalised CO_2_ permeance of 0.81 over 365 days [79]. Additionally, by widening the rigidified PIM-1 through the introduction of network fillers composed of nanosized UiO-66 − NH_2_ and carboxylated PIM-1, the PIM-1 TFN also shows improved anti-aging performance, with a normalised CO_2_ permeance of 0.94 over 63 days [145].

## Conclusions and Perspectives

Recent literature on the modification strategies of PIM-1 membranes is summarised and critically reviewed to show the progress for enhancing key properties in gas separation including permeability/selectivity, anti-physical aging, and anti-plasticisation abilities. Based on the meta-analysis of the results reported by the relevant state-of-the-arts, pros and cons of the developed modification strategies are analysed and discussed.

Among the four strategies, incorporating heterogeneous fillers (fabricating MMMs) provides the most prominent effect in improve the overall separation performance for CO_2_/N_2_ and CO_2_/CH_4_, with 80% and 85% of modified PIM-1 membranes lying beyond the 2008 upper bound, respectively. The improvement is mainly due to (i) rigidification of PIM-1 chains by interaction at the heterogeneous interface, and (ii) CO_2_-selective transportation facilitated by the CO_2_-affinity sites on fillers. Based on the separation performance, the beneficial effect of fillers was identified with the order of crystallised microporous fillers > organic porous fillers > impermeable fillers. The positive effects of fillers on separation are twofold: (i) porous structures of fillers could facilitate the accessibility of the CO_2_-affinity sites in them boosting CO_2_-selective mass transfer in MMMs, and (ii) the introduction of highly rigid microstructures narrowing down pore-size distribution make it more favourable for breaking the permeability/selectivity trade-off.

Regarding anti-aging/plasticisation, the introduction of fillers in PIM-1 aids in retarding PIM-1 chains’ mobility due to the interactions between the fillers and the polymer at their interface. Especially, fillers having larger surface areas (e.g., 1D or 2D fillers), as well as stronger interaction with PIM-1 (e.g., by covalent bonding or hydrogen bonding), are more favourable for improving such interfacial interactions. Also, fillers with permanent porous structures are favourable as well for slowing down overall tendency of aging and plasticisation.

Polymer chains modification and post-modification are two effective strategies for improving the membranes’ selectivity, as the enhanced molecular interaction leads to a higher rigidity of the polymer chains. On the other hand, both strategies decrease the free volume, and thus reduce membrane permeability. As a consequence, only about 39% and 40% of the membranes modified by these two strategies exceed the CO_2_/N_2_ upper bound, respectively, although some of them are more effective for separation of smaller gas pairs. It is worth noting that the enhanced molecular interaction in relevant PIM-1 exhibits insignificant effect on anti-aging, and in some cases exarate the densification of the membranes over time. There was no clear trend in the effects of the two strategies on anti-plasticisation, which might be due to the difference in the stability of the intermolecular chains between the polymer backbones constructed by the various modification strategies.

PIM-1 blends show the least effectiveness in enhancing the overall separation performance (selectivity vs. permeability), and over 81% and 67% of PIM-1 blends are below the 2008 upper bound for CO_2_/N_2_ and CO_2_/CH_4_, respectively. This could be due to the inclusion of flexible guest components (e.g., rubbery polymers and ILs) widening pore-size distribution. In most cases of the blends, the selectivity was enhanced thanks to features of guest components such as the presence of sites with CO_2_-affinity (e.g., in some guest rubbery polymers) and/or high CO_2_ selective sorption capacity (e.g., in ILs). Additionally, due to the reduced movability of PIM-1 chains caused by the interaction between guest polymer chains and PIM-1, some PIM-1 blends exhibited enhanced anti-plasticisation against condensable CO_2_.

Compared to thick films, thin film membranes of PIM-1 are more suitable for practical applications due to the combination of the high permeance and high mechanical strength. Accordingly, here we comment on the fabrication techniques and the separation performance of PIM-1 thin film membranes (with the thickness of < 5 μm) as well. Key aspects impairing the performance of PIM-1 thin film composites/nanocomposite (TFCs/TFNs) were identified including penetration of PIM-1 into the substrate, fillers applicability, and accelerated physical aging. Certain treatments of the substrate, such as solvent pre-impregnation and application of a gutter layer, were found effective to alleviate PIM-1 penetration. Due to the low thickness of the PIM-1 layer, low-dimensional fillers are preferred in preparing PIM-1 TFNs to avoid defects. Besides, compared to thick films, thin film membranes typically show accelerated physical aging, with the normalised CO_2_ permeability dropped to 0.01 − 0.5 after 100 days. Similar to the MMMs, adding fillers could partially alleviate the physical aging in thin film membranes.

In summary, PIM-1 membranes hold great potential in selective CO_2_ separation. Current research has shown that PIM-1 MMMs, combining heterogeneous fillers, are rather effective to address issues such as low selectivity, physical aging, and plasticisation compared to other modification strategies. Therefore, further research should focus on exploring the combination of fillers and polymer matrix with the optimised synergy, developing fillers with higher size-sieving/selective adsorption capabilities, constructing stronger and wider range of filler-induced polymer rigidification, and developing MMMs with high loading of porous fillers without aggregations/defects. Additionally, considering the advantages and characteristics of thin film membranes, the modification strategies may be revised and translated to be applied to improving thin film membranes, which can be assessed under conditions close to practical settings. Additionally, membrane fabrication techniques are equally important to the design/modification of membrane materials, and hence this aspect deserve more attention for techniques development to prepare ultrathin and defect-free PIM-1 membranes at scales. Finally, it is crucial to evaluate the membranes under realistic conditions close to relevant industrial applications to assess their viability for commercial gas separation processes in the real world.

## Supplementary Information

Below is the link to the electronic supplementary material.

Supplementary file1 (DOCX 287 KB)
