# Supplementary material for: Membranes of Polymer of Intrinsic Microporosity PIM-1 for Gas Separation: Modification Strategies and Meta-Analysis
Source: Nanomicro Lett. 2025 Jan 23;17:114. doi: 10.1007/s40820-024-01610-2 (PMC11757663; doi:10.1007/s40820-024-01610-2)
Supplement: Supplementary file 1 — Supplementary file1 (DOCX 287 KB) [file 40820_2024_1610_MOESM1_ESM.docx]

Supporting Information for

**Membranes of Polymer of Intrinsic Microporosity PIM-1 for Gas Separation: Modification Strategies and Meta-Analysis**

Boya Qiu^1^, Yong Gao^2^, Patricia Gorgojo^1, 3, 4,^*, Xiaolei Fan^1, 2, 5,^*

^1^ Department of Chemical Engineering, Faculty of Science and Engineering, The University of Manchester, Manchester M13 9PL, United Kingdom

^2^ Institute of Wenzhou, Zhejiang University, Wenzhou 325006, P. R. China

^3^ Instituto de Nanociencia y Materiales de Aragón (INMA) CSIC-Universidad de Zaragoza, Mariano Esquillor, 50018 Zaragoza, Spain

^4^ Departamento de Ingeniería Química y Tecnologías del Medio Ambiente, Universidad de Zaragoza, Pedro Cerbuna 12, 50009 Zaragoza, Spain

^5^ Ningbo China Beacons of Excellence Research and Innovation Institute, University of Nottingham Ningbo China, 211 Xingguang Road, Ningbo 315048, P. R. China

*Corresponding authors. E-mails: [pgorgojo@unizar.es](mailto:pgorgojo@unizar.es) (Patricia Gorgojo); [xiaolei.fan@manchester.ac.uk](mailto:xiaolei.fan@manchester.ac.uk) (Xiaolei Fan)

**Supplementary Figures**

**Table S1** Gas separation performance of thick PIM-1 membranes (at 20−40 ℃ and 1−4 bar)

|  | Membrane | Gas permeability (barrer) | | | | | | Selectivity | | | Refs. |
| --- | --- | --- | --- | --- | --- | --- | --- | --- | --- | --- | --- |
|  |  | He  2.59 Å | H_2_  2.90 Å | O_2_  3.44 Å | N_2_  3.66 | CH_4_  3.81 Å | CO_2_  3.63 Å | O_2_/N_2_ | CO_2_/N_2_ | CO_2_/ CH_4_ |  |
| Unmodified PIM-1 | PIM-1 | 660 | 1300 | 370 | 92 | 125 | 2300 | 4.0 | 25 | - | [S1] |
|  | PIM-1 | 760 | 1630 | 580 | 180 | 310 | 4390 | 3.2 | 24 | - | [S2] |
|  | PIM-1 | - | - | 1133 | 353 | - | 5366 | 3.2 | 15 | - | [S3] |
|  | PIM-1 | 1061 | 2332 | 786 | 238 | 360 | 3496 | 3.3 | 14.7 | 9.7 | [S4] |
|  | PIM-1 | 1500 | 3600 | 1300 | 340 | 430 | 6500 | 3.3 | 15 |  | [S5] |
|  | PIM-1 |  |  | 1017 | 342 | 555 | 5093 | 3.0 | 14.9 | 9.2 | [S6] |
|  | PIM-1 |  | 3731 | 1172 | 309 | 431 | 6601 |  |  | 15.2 | [S7] |
|  | PIM-1 |  | 2785 | 1012 | 303 | 417 | 5606 | 3.3 | 18.5 | 13.4 | [S8] |
|  | PIM-1 |  | 3195 | 1089 | 325 | 418 | 5622 | 3.4 | 17.3 | 13.5 | [S9] |
|  | PIM-1 |  |  | 1521 | 687 |  | 8461 | 2.2 | 12.3 |  | [S10] |
|  | PIM-1 | 1048 |  | 928 | 279 | 401 | 6211 | 3.3 | 22.3 | 15.5 | [S11] |
|  | PIM-1 | 800 |  | 510 | 120 | 170 | 2790 | 4.4 | 23 | 16.4 | [S12] |
|  | PIM-1 |  |  |  |  |  | 4100^*^ |  | 15^*^ |  | [S13] |
|  | PIM-1 | 1577 | 3365 | 985 | 248 | 362 (423^*b^) | 5919 (5600^*b^) | 4.0 | 24 | 16 (13^*b^) | [S14] |
|  | PIM-1 |  |  |  | 228 | 310 | 3799 |  | 16.6 | 12.2 | [S15] |
|  | PIM-1 (ethanol treated) | 1760 |  | 1950 | 660 | 1000 | 11400 | 3.0 | 17 | 11.4 | [S12] |
|  | PIM-1 |  |  | 562.2 | 163.6 | 322.2 | 3294.7 | 3.4 | 20.1 | 10.2 | [S16] |
|  | PIM-1 |  | 3537 | 1072 | 351 | 536 | 6576 | 3.0 | 18.7 | 12.3 | [S17] |
|  | PIM-1 |  |  |  |  |  | 3815 |  | 20 | 14 | [S18] |
|  | PIM-1 |  |  | 2179 | 912 | 1374 | 10683 | 2.4 | 11.7 | 7.8 | [S19] |
|  | PIM-1 |  | 1936 |  | 148 | 229 | 3425 |  | 23.1 | 15.0 | [S20] |
|  | PIM-1 |  | 4000 | 1073 | 338 | 502 | 5506 | 3.2 | 16.3 | 11.0 | [S21] |
|  | PIM-1 | 1660 | 4270 | 1620 | 540 | 1000 | 8570 | 3 | 15.9 | 8.6 | [S22] |
|  | PIM-1 |  |  |  |  |  | 2430 |  | 16.99 |  | [S23] |
|  | PIM-1 |  |  |  | 240 |  | 4700 |  | 19.3 |  | [S24] |
|  | PIM-1 |  | 3511 | 1049 | 269 | 366 | 3934 | 3.9 | 14.6 | 11.0 | [S25] |
|  | PIM-1 |  |  |  |  |  | 3672 |  | 16.5 (15.5^d^) | 10.6 (9.6^e^) | [S26] |
|  | PIM-1 | 1170 | 2710 | 875 | 219 | 286 | 4770 | 4.0 | 21.8 | 16.7 | [S27] |
|  | PIM-1 |  |  |  | 198 | 315 | 3795 |  | 19 | 12 | [S28] |
|  | PIM-1 |  |  | 1130 | 397 | 652 | 7440 | 2.8 | 19 | 11 | [S29] |
|  | PIM-1 |  | 2504 | 965.5 | 255.9 | 335.0 | 4087 | 3.77 | 16.0 | 12.2 | [S30] |
|  | PIM-1 |  | 1820 | 802 | 190 | 210 | 3054 | 4.2 | 16.1 | 14.5 | [S31] |
|  | PIM-1 |  | 1784 | 590 | 182 | 218 (239^*b^) | 3364 (3422^*b^) | 3.2 | 18.5 | 15.4 (14.3^*b^) | [S32] |
|  | PIM-1 |  | 7072 | 2326 | 693 | 1080 | 11371 | 3.4 | 16.4 | 10.5 | [S33] |
|  | PIM-1 | 1369 | 3580 | 1790 | 727 | - | 8310 | 2.5 | 11 |  | [S34] |
|  | PIM-1 |  |  |  | 211 |  | 3402 |  | 16.1 |  | [S35] |
|  | PIM-1 |  |  |  | (231^a^) | (288^b^) | (4020^a^) (3895^b^) |  | (15.6^a^) | (13.9^b^) | [S35] |
|  | PIM-1 |  |  |  | 297 (321^a^) |  | 8095 (3882^a^) |  | 13 (14.7^a^) |  | [S36] |
|  | PIM-1 |  |  |  |  | (330^b^) | (6400^b^) |  |  | (20.3^b^) | [S37] |
|  | PIM-1 |  |  |  | 230 |  | 3277 |  | 16.2 |  | [S38] |
|  | PIM-1 | 2000 | 4980 | 2140 | 607 | 914 | 12100 |  | 19.9 | 13.2 | [S39] |
|  | PIM-1 |  | 2219 |  | 224 | 363 | 4521 (4336^e^) |  | 20.2 | 12.5 (11.5^e^) | [S40] |
|  | PIM-1 |  |  | 1072 | 351 | 536 (408^b^) | 6576 (5772^b^) | 3.0 | 18.7 | 12.3 (12.4^b^) | [S41] |
|  | PIM-1 |  | 3274 | 1396 | 483 | 789 | 9896 | 2.9 | 20.5 | 12.5 | [S42] |
|  | PIM-1 |  | 2696 | 712 | 166 | 204 | 3375 | 4.3 | 20.4 | 16.6 | [S43] |
|  | PIM-1 |  | 2753 | 1028 | 375 | 644 | 5811 | 2.7 | 15.5 | 9.0 | [S44] |
|  | PIM-1 |  |  |  | 195.4 |  | 3694.5 |  | 18.9 |  | [S45] |
|  | PIM-1 |  |  |  |  |  | (8994^f^) (8263^b^) |  | (14.9^f^) | (13.6^b^) | [S46] |
|  | PIM-1 |  |  |  |  |  | 1250 |  | 19.8 |  | [S47] |
|  | PIM-1 |  |  |  | 306.2 |  | 4131 |  | 13 |  | [S48] |
|  | PIM-1 |  |  |  | 113.9 |  | 3027.7 |  | 26.6 |  | [S49] |
|  | PIM-1 | 1830 | 4710 | 2130 | 773 | 1300 | 12800 | 2.76 | 16.5 | 9.8 | [S50] |
|  | PIM-1 |  |  |  |  |  | (7500^g^) |  | (14^g^) |  | [S51] |
|  | PIM-1 |  |  | 844 | 211 |  | 4200 | 4.0 | 20 |  | [S52] |
|  | PIM-1 |  |  | 1545 | 486 |  | 7340 | 3.2 | 15.1 |  | [S53] |
|  | PIM-1 |  |  |  |  | (450^b^) | (5920^b^) |  |  | (13.1^b^) | [S54] |
|  | PIM-1 |  |  |  | 650 | 1490 | 11840 |  | 18.23 | 7.94 | [S55] |
|  | PIM-1 |  |  |  | 204 | 273 | 4375 |  | 21.4 | 16.0 | [S56] |
|  | PIM-1 |  | 1400 | 460 | 140 | 220 | 2800 | 3.2 | 20 | 13 | [S57] |
|  | PIM-1 |  |  |  |  | 363 | 4533 (4391^e^) |  |  | 12.5 (11.6^e^) | [S58] |
|  | PIM-1 |  |  |  |  |  | 4521 (4336^e^) |  |  | 12.45 (11.50^e^) | [S59] |
|  | PIM-1 |  |  |  | 85.9 | 104.7 | 2345.4 |  | 27.3 | 22.4 | [S60] |
|  | PIM-1 |  | 575.2 | 130.1 | 27.2 | 29.0 | 980.7 |  | 36.0 | 33.8 | [S61] |
|  | PIM-1 |  |  |  |  |  | (5917^b^) |  |  | (13.1^b^) | [S62] |
|  | PIM-1 |  |  | 802 | 229 | 258 | 4259 | 3.5 | 18.6 | 16.5 | [S63] |
|  | PIM-1 |  | 3380 | 1250 | 400 | 590 | 7200 | 3.1 | 18.0 | 12.0 | [S64] |
|  | PIM-1 |  |  |  |  |  | 7210 |  | 18 |  | [S65] |
|  | PIM-1 (sub-ambient temperature) | 1000 | 1557 | 159 | 17 | 21 | 1380 | 8 | 81.2 |  | [S66] |
|  | PIM-1 |  |  |  |  | (543^b^) | (6190^b^) |  |  | (11.7^b^) | [S67] |
|  | PIM-1 (methanol treated) |  | 3949 | 1257 | 337 | 472 | 6957 | 3.7 | 20.7 | 14.8 | [S43] |
|  | PIM-1 |  |  |  |  | 516 | 7135 (6007^*e^) |  |  | 13.8 (14.7^*e^) | [S68] |
|  | PIM-1 |  |  |  |  | (543^b^) | (6190^b^) |  |  | (11.7^b^) | [S69] |
|  | PIM-1 |  |  |  |  |  | 5800 |  |  | 11.5 | [S70] |
|  | PIM-1 |  |  |  |  | 660 (810^b^) | 9000 (8250^b^) |  |  | 13.5 (10.2^b^) | [S71] |
|  | PIM-1 |  |  |  | 205 |  | 4110 |  | 20 |  | [S72] |
|  | PIM-1 |  | 1530 | 880 | 273 | 380 | 5105 |  | 18.7 | 13.4 | [S73] |
|  | PIM-1 |  |  |  |  | (758^b^) | (6578^b^) |  |  | (8.7^b^) | [S74] |
|  | PIM-1 |  | 4330 | 1353 | 373 | 442 | 6595 | 3.63 | 17.7 | 14.9 | [S75] |
|  | PIM-1 |  | 4381 | 1686 | 434 | 527 | 8287 | 3.9 | 19.1 | 15.7 (14.3^b^) | [S76] |
|  | PIM-1 | 1336 | 2772 | 768 | 203 | 299 | 3760 | 3.8 | 18.5 | 12.6 | [S77] |
|  | PIM-1 |  |  |  |  |  | (4216^e^) |  |  | (10.1^e^) | [S78] |
|  | PIM-1 |  |  |  | 288.0 | 371.7 | 5181.6 |  | 18.0 | 13.9 | [S79] |
|  | PIM-1 |  |  |  |  | 1453 | 9336 |  |  | 6.43 | [S80] |
|  | PIM-1 |  |  |  | 227.5 (251.4^h^) |  | 3842.3 (3253.1^h^) |  | 16.9 (12.9^h^) |  | [S81] |
|  | PIM-1 |  |  |  |  |  | 6605.9 (4902^g^) |  | 16.1 (18.2^g^) | 12.0 | [S82] |
|  | PIM-1 |  |  |  |  |  | 5167 |  |  |  | [S83] |
|  | PIM-1 |  |  |  | (363^a^) | (563^b^) | (6780^a^) (6411^b^) |  | (18.6^a^) | (11.5^b^) | [S84] |
|  | PIM-1 |  |  |  |  |  | (3945^e^) |  |  | (12.5^e^) | [S85] |
|  | PIM-1 |  | 3250 |  | 249.9 | 318 | 4868 |  | 19.5 | 15.3 | [S86] |
|  | PIM-1 |  |  |  |  | (611^b^) | (6887^b^) |  |  | (11.5^b^) | [S87] |
|  | PIM-1 |  | 3255^*^ |  | 364^*^ (387^a^) | 632^*^ (590^b^) | 7311^*^ (7323^a^) (7195 ^b^) |  | (18.9^a^) | (12.3^b^) | [S88] |
|  | PIM-1 |  |  |  |  |  | 4310 |  |  | 22.6 | [S89] |
|  | PIM-1 |  |  |  |  |  | (8000^b^) |  | (18^d^) | (10^e^) | [S90] |
|  | PIM-1 |  |  |  |  |  | (3980^a^) |  | (23.1^a^) |  | [S91] |
|  | PIM-1 |  |  |  | 170.9 (170.4^a^) |  | 3872.8 (3673.7^a^) |  | 22.7 (21.6^a^) |  | [S92] |
|  | PIM-1 |  | 2378 |  |  | 305.3 | 4822 |  | 15.8 | 11.3 | [S93] |
|  | PIM-1 |  |  |  | 309 (284^f^) | 416 (385^b^) | 6065 (4786^f^) (4635^b^) |  | 19.6 (16.1^f^) | 14.6 (12.3^b^) | [S94] |
|  | PIM-1 |  |  |  | 313.1 |  | 5360.6 |  | 17.0 |  | [S95] |
|  | PIM-1 |  |  |  | 210 (207^a^) | 224 (238^b^) | 4387 (4210^a^) (4270^b^) |  | 20.9 (17.94^a^) | 19.6 (20.3^b^) | [S96] |
|  | PIM-1 |  | 2058.2 | 589.2 | 142.2 | 199.0 | 2789.5 |  | 19.6 | 14.0 | [S97] |
|  | PIM-1 |  |  |  |  |  | (6800^f^) |  | (18^f^) |  | [S98] |
|  | PIM-1 |  |  |  |  |  | 4187 |  | 17.4 |  | [S99] |
|  | PIM-1 |  |  |  |  | 259 | 4654 |  |  | 18.1 | [S100] |
|  | PIM-1 |  |  | 787 | 299 | 511 | 4772 |  | 15.9 | 9.3 | [S101] |
|  | PIM-1 |  | 2631 |  | 578 | 832 | 7842 |  | 13.6 | 9.7 | [S102] |
|  | PIM-1 |  |  |  |  |  | 4132 |  | 13.5 |  | [S103] |
|  | PIM-1 |  |  |  |  |  | 2795 |  | 16 |  | [S104] |
| Chain modification | Carboxylated PIM-1 | 153 | 408 | 110 | 24 | - | 620 | 4.6 | 26 | - | [S34] |
|  | TZ−PIM-1 | - | - | - | - | - | - | - | 30^*^ (40^a*^) | 22^*^ (22^b*^) | [S105] |
|  | Thio−PIM-1 | 270 | 610 | 140 | 37 | 56 | 1120 | 3.8 | 30 | 20 | [S106] |
|  | MTZ−PIM |  |  | 269.6 | 62.7 (42.0^c^) |  | 1391 (1674^c^) | 4.3 | 22.2 (39.9^c^) |  | [S10] |
|  | AO−PIM-1 | 412 | 912 | 147 | 33 | 34 (35^*b^) | 1153 (830^*b^) | 4.5 | 35 | 34 (24^*b^) | [S14] |
|  | PIM-C1 | 2880 | 9870 | 3410 | 980 | 1300 | 18900 | 3.5 | 19.3 | 14.4 | [S22] |
|  | HCPIM(PIM-1−COOH) |  | 90.53 | 9.80 | 1.80 | 3.82 | 96.43 | 5.4 | 53.6 | 25.2 | [S25] |
|  | PIM-1−M |  | 3710 | 2180 | 850 | 1525 | 14180 | 2.6 | 17 | 9 | [S107] |
|  | PIM-1−MBr |  | 1300 | 480 | 115 | 185 | 3200 |  | 27 | 17 | [S107] |
|  | Brominated vinylated PIM-1 |  | 675 | 305 | 90 | 130 | 1890 |  | 21 | 15 | [S107] |
|  | Thiophenated vinylated PIM-1 |  | 775 | 234 | 70 | 125 | 1735 |  | 25 | 14 | [S107] |
|  | Vinylated PIM-1 |  | 1080 | 410 | 130 | 260 | 3240 |  | 26 | 12 | [S107] |
|  | PIM-1 160℃, 33 min |  |  |  |  | 694 | 7478 |  |  | 11.9 | [S108] |
|  | PIM-1 120℃, 81 min |  |  |  |  | 293.5 | 5151 |  |  | 17.65 | [S108] |
|  | Adamantane-AO−PIM-1 |  | 1360.3 | 366.5 | 79.5 | 82.5 | 2483.6 |  | 31.2 | 30.1 | [S61] |
|  | PIM-1−COOH |  | 270 | 44 | 9.0 | 9.0 | 290 | 5.0 | 32 | 33 | [S57] |
|  | PIM-1 160℃, 40 min, N_2_ flow |  |  |  |  |  | 4835 |  | 55.5 |  | [S109] |
|  | PIM-1−SO_3_H |  | 2607 | 324 | 53 | 35 | 1388 |  | 26.2 | 39.7 | [S75] |
|  | PIM-1−Py |  | 556.7 | 127.4 | 36.7 |  | 768.5 |  | 20.9 | 17.5 | [S97] |
|  | PIM-1−MePy |  | 272.5 | 53.4 | 13.6 |  | 295.1 |  | 21.8 | 15.0 | [S97] |
| Post-modification | Crosslinked PIM-1/azide |  |  | 38 | 8 |  | 219 | 4.8 | 27.4 |  | [S110] |
|  | DC-induced thermal crosslinked cPIM-1 |  |  | 231 | 49.9 | 52.6 | 1291 | 4.6 | 28.8 | 24.6 | [S6] |
|  | PIM-UV 30 min |  | 2274 | 189 | 27.7 | 23.1 (20.4^b^) | 724 (745^b^) | 6.8 | 26.1 | 31.3 (29.3^b^) | [S7] |
|  | Thermally crosslinked PIM-1 |  | 3872 | 582 | 96 | 73 | 4000 |  | 41.7 | 54.8 | [S43] |
|  | UV/oxidize-modified PIM-1 |  | 2104 | 513 | 84 | 85 | 2394 | 6.1 | 28.4 | 28.1 | [S9] |
|  | TOX-PIM-1 |  | 1820 | 245 | 30 | 16 | 1104 | 8.1 | 37 (45^*a^) | 69 (60^*b^) | [S111] |
|  | PIM-1(Na)-Al |  |  |  |  |  | 907 |  | 30.8 |  | [S19] |
|  | Amine-PIM-1 | 838 | 2180 | 664 | 176 | 240 | 1030 |  | 5.83 | 4.28 | [S39] |
|  | PIM-1−NH_2_ |  | 1450 | 430 | 134 | 210 | 840 | 3.2 | 6.3 | 4 | [S64] |
|  | PIM-1−*t*BOC |  | 130 | 19 | 3.6 | 5.0 | 100 | 5.3 | 28 | 20 | [S64] |
|  | PIM-1−deBOC(acid) |  | 1700 | 500 | 160 | 260 | 1400 | 3.0 | 8.6 | 5.6 | [S64] |
|  | PIM-1−deBOC(thermal) |  | 2000 | 500 | 120 | 170 | 2300 | 4.6 | 20 | 14 | [S64] |
|  | PIM-1-450 |  | 200^*^ |  | 0.6^*^ | 0.15^*^ | 30^*^ |  | 50^*^ | 200^*^ | [S112] |
|  | SCPIMs |  |  |  | 69 | 98 | 4008 |  | 58.1 | 40.9 | [S56] |
|  | Crosslinked PIM-1-BM-70 |  |  | 11.1 | 1.7 | 1.1 | 48.3 | 6.5 | 30.1 | 43.9 | [S63] |
|  | F-PIM-1 | 754 | 326 | 7.54 | 0.88 | 0.2 | 22.3 | 8.6 | 25.3 | 111.5 | [S77] |
|  | PIM-1/Al_2_O_3_ |  | 2492 | 188 | 21.3 | 11.1 (13.9^b^) | 624 (698^b^) | 8.8 | 29.3 | 56.2 (50.2^b^) | [S76] |
|  | PIM-1−NH_2_ |  |  |  |  | 300 (33.5^b^) | 1070 (845^b^) |  |  | 3.6 (25.2^b^) | [S71] |
|  | PIM-1/tannic acid |  |  |  |  |  | (3253^e^) |  |  | (19.0^e^) | [S78] |
|  | PIM-1/TiO_2_ |  |  |  |  |  | 10659.6 (7036^g^) |  | 15.5 (18.0^g^) | 11.3 | [S82] |
|  | PIM-1-O_3_ |  | 1294 |  | 13.81 | 10.7 | 443 |  | 32.08 | 41.4 | [S86] |
|  | Ester-crosslinked PIM-1−COOH |  |  | 984 | 302 | 416 | 6347 |  | 21.0 | 15.2 | [S101] |
|  | NA-PIM-1 |  |  |  | 161.4 (178.4^a^) |  | 4593.2 (4121.7^a^) |  | 28.5 (23.1^a^) |  | [S92] |
|  | NA-PIM-1-Zn-PEI |  |  |  | 98.9 (104.4^a^) |  | 3125.9 (2848.1^a^) |  | 31.6 (27.3^a^) |  | [S92] |
|  | Aminated PIM-1 |  | 1135 |  | 85 | 229 | 2590 |  | 30.8 | 13.1 | [S102] |
|  | f-PIM-1 | 710 | 741 | 27 | 3.1 | 1.6 | 102 | 8.7 | 32.9 | 63.8 | [S113] |
| Blends | PIM-1/Matrimid® (10%) |  |  | 400 |  |  | 1953 | 4.0 | 20 | 16 | [S18] |
|  | PIM-1/Matrimid® (10%)−TETA |  | 395 | 32 | 4.3 | 3.4 | 41 | 7.4 | 9.5 | 12.1 | [S114] |
|  | cPIM-1/Torlon (10%) |  |  | 185 | 42 | 42 | 1013 | 4.4 | 24.0 | 23.9 | [S115] |
|  | cPIM-1/Matrimid® |  |  |  |  | 57 (53.5^b^) | 982 (905^b^) |  |  | 17.2 (16.9^b^) | [S116] |
|  | PIM-1/PEG |  |  |  | 115 | 50 | 1952 |  | 17.0 | 39.0 | [S15] |
|  | PIM-1/sPPSU |  | 1257 | 257 | 57 | 61 (60^*b^) | 1429 (1800^*b^) | 4.5 | 25.1 | 23.4 (30^*b^) | [S21] |
|  | PIM-b-PEG |  |  | 370 | 140 | 270 | 3000 | 2.8 | 22.3 | 11.1 | [S117] |
|  | PIM-1-PEG copolymer |  |  | 101 | 27 | 43 | 812 | 3.7 | 30 | 19 | [S29] |
|  | PIM-1/[SC_6_mim][STf_2_N] |  |  | 310 | 90 | 165 | 2240 | 3.4 | 25 | 14 | [S29] |
|  | PIM/PEI (8:2) |  | 3489 | 1126 | 332 | 511 | 5765 | 3.4 | 17.4 | 11.3 | [S33] |
|  | PIM-1/MEEP80 |  | 597 | 274 | 117 | 292 | 2446 | 2.3 | 20.9 | 8.4 | [S44] |
|  | PIM-1-IL |  |  | 102 | 23 |  | 817 | 4.5 | 35.5 |  | [S53] |
|  | PIM-1/MEEP100 |  |  |  |  |  | 5340 |  | 24 |  | [S65] |
|  | PIM-1/IL |  |  |  |  |  | 2090 |  |  |  | [S83] |
|  | PIM-1/[SAPTMS][SAc] |  |  |  | 87 (90^a^) | 95 (94^b^) | 2754 (2565^a^) (2543^b^) |  | 31.7 (27.05^a^) | 29.0 (28.5^b^) | [S96] |
| MMMs | PIM-1/silica | 2000 | 5060 | 2330 | 880 | - | 10100 | 2.7 | 12.0 | - | [S118] |
|  | PIM-1/CC3 (10:1) | 740 |  | 560 | 150 | 230 | 3250 | 3.8 | 22 | 14.1 | [S12] |
|  | PIM-1/CC3 (10:3) | 820 |  | 820 | 270 | 480 | 5430 | 3.0 | 20 | 11.3 | [S12] |
|  | PIM-1/CC3 (10:1) (ethanol treated) | 2310 |  | 3150 | 1350 | 2050 | 18780 |  | 14 | 9.2 | [S12] |
|  | PIM-1/CC3 (10:3) (ethanol treated) | 4380 |  | 6810 | 3270 | 7220 | 37400 | 2.1 | 11 | 5.2 | [S12] |
|  | AO−PIM-1/UiO-66(Zr) |  | 1069 | 206 | 51 | 55 | 1380 | 4 | 27.1 | 25 | [S8] |
|  | f-SWCNTs/PIM-1 | 1355 |  | 1680 | 713 | 1483 | 12274 | 2.4 | 17.2 | 8.3 | [S11] |
|  | PIM-1/silicalite-1(MFI) |  | 894 | 351 | 83 | 183 | 2530 | 4.2 | 30 |  | [S119] |
|  | PIM-1/ZIF-8 | 1430 | 2980 | 870 | 195 | 230 | 4270 | 4.5 | 21.9* | 18.6 | [S120] |
|  | PIM-1/PAF-1 |  |  |  |  |  | 15000^*^ |  | 11^*^ |  | [S13] |
|  | PIM-1/POSS |  |  |  |  |  | 6730 (5650^*b^) |  | 16^*^ | 11^*^ (11^*b^) | [S121] |
|  | UV-PIM-1/ZIF-71 |  |  | 599.4 | 93.6 | 72.1 (67.8^b^) | 2545.7 (2224.3^b^) | 6.4 | 27.2 | 35.3 (32.8^b^) | [S16] |
|  | PIM-1/UiO-66 |  |  |  |  |  | 5340 |  | 21^*^ |  | [S122] |
|  | PIM-1/Ti-UiO-66 |  |  |  |  |  | 13540 |  | 20^*^ |  | [S122] |
|  | PIM-1/crosslinked MOF-74 |  | 11469 | 2251 | 742 | 1114 | 21269 | 3.0 | 19.1 | 28.7 | [S17] |
|  | PIM-1/g-C_3_N_4_ |  | 3830 |  | 354 | 503 | 5785 |  | 16.3 | 11.5 | [S20] |
|  | PIM-1/HCP |  |  |  | 243 |  | 4700 |  | 19.3 |  | [S123] |
|  | PIM-1/TSIL@NH_2_−MIL-101(Cr) |  |  |  |  |  | 2979 |  | 37.24 |  | [S23] |
|  | PIM-1/BILP-101 |  |  |  | 470 |  | 7200 |  | 15.3 |  | [S24] |
|  | PIM-1/UiO-66 | 2120 | 4560 | 1360 | 368 | 527 | 7610 | 3.7 | 20.7 | 14.4 | [S27] |
|  | PIM-1/UiO-66−NH_2_ | 1340 | 3130 | 1090 | 303 | 425 | 6340 | 3.6 | 20.9 | 14.9 | [S27] |
|  | PIM-1/UiO-66−(COOH)_2_ | 1820 | 3740 | 1220 | 296 | 401 | 6090 | 4.1 | 20.6 | 15.2 | [S27] |
|  | PIM-1/PEG-POSS |  |  |  | 43 | 44 | 1309 |  | 31 | 30 | [S28] |
|  | PIM-1/PhE-POSS |  |  | 900* | 280* | 400* | 5000* | 3.2* | 17.9* | 12.5* | [S124] |
|  | PIM-1/SNW-1 |  |  |  |  |  | 7553 |  | 22.7 (22.1^d^) | 13.5 (12.9^e^) | [S26] |
|  | PAO−PIM-1/UiO-66−NH_2_ |  |  |  | 306 | 366 | 8425 |  | 27.5 | 23.0 | [S125] |
|  | PIM-1/OPAS |  | 2476 | 789.5 | 157.1 | 201.2 | 3266 (3600^*b^) | 5.02 | 20.8 (14.9^*a^) | 16.2 (9.0^*b^) | [S30] |
|  | PIM-1/MFM-300(V^III^) |  |  |  |  |  | 4450 |  | 28.04 |  | [S126] |
|  | PIM-1/UiO-66−NH_2_ |  | 2641 | 658 | 104 | 102 | 2869 (2573^*b^) |  | 27.5 (25.4^*a^) | 28.3 (31.4^*b)^ | [S31] |
|  | PIM-1/β-CD |  | 3889 | 1647 | 481 | 716 (770^*b^) | 8812 (8907^*b^) | 3.5 | 18.3 | 12.3 (11.6^*b^) | [S32] |
|  | PIM-1/DNPC |  |  |  | 3266 |  | 40544 |  | 12.4 |  | [S35] |
|  | PIM-1/NUS-8 nanosheet |  |  |  | (389^a^) | (463^b^) | (6725^a^) (6462^b^) |  | (26.8^a^) | (30.1^b^) | [S35] |
|  | PIM-1/TPFC−CH_2_NH_2_ |  |  |  |  |  | 7730 |  | 45.9 | 36.4 | [S127] |
|  | PIM-1/Azo-DMOF-1 |  |  |  | 433 (371^a^) |  | 8095 (7500^a^) |  | 18.7 (20.2^a^) |  | [S36] |
|  | PIM-1/rGO−OA |  |  |  |  | (360^b^) | (5700^b^) |  |  | (17.4^b^) | [S37] |
|  | PIM-1/GO |  |  |  | 50 |  | 6169 |  | 123.5 |  | [S38] |
|  | PIM-1/ZIF-67 |  | 3542 |  | 215 | 310 | 5206 (5206^e^) |  | 24.2 | 16.8 (14.9^e^) | [S40] |
|  | PIM-1/crosslinked UiO-66−NH_2_12h_ |  |  | 1942 | 555 | 828 (830^b^) | 15815 (15813^b^) | 3.5 | 28.5 | 19.1 (19.2^b^) | [S41] |
|  | PIM-1/crosslinked UiO-66−NH_2_72h_ |  |  | 945 | 230 | 392 | 12498 | 4.1 | 54.2 | 31.9 | [S41] |
|  | PIM-1/ K_2_B_12_H_12_ |  | 4025 | 1831 | 772 | 1436 | 12954 | 2.4 | 16.8 | 9.0 | [S42] |
|  | PIM-1/MAPDA |  |  |  | 329.0 (117.7^*a^) |  | 7861.9 (4807.5^*a^) |  | 23.9 (40.1^*a^) |  | [S45] |
|  | PIM-1/6FDA-DAM/ZIF-8 |  |  |  |  |  | (2802^f^) (2891^b^) |  | (18.1^f^) | (26.6^b^) | [S46] |
|  | PIM-1/DMBA-Silica |  |  |  |  |  | 7930 |  | 16.8 |  | [S47] |
|  | PIM-1/PDASS |  |  |  | 47.3 |  | 1678 |  | 35 |  | [S48] |
|  | UiO-66−CN@sPIM-1 |  |  |  | 596 (225.5^a^) |  | 16121.3 (12063.3^a^) |  | 27.0 (53.5^a^) |  | [S49] |
|  | PIM-1/MIL-101 | 6900 |  | 6540 | 2320 | 3040 | 35600 | 2.82 | 15.3 | 11.7 | [S50] |
|  | PIM-1/pDCX |  | 9710 | 3800 | 1130 | 1650 | 20550 | 3.4 | 18.2 | 12.4 | [S128] |
|  | PIM-1/OH−pDCX |  | 5230 | 1470 | 300 | 380 | 8510 | 4.8 | 28.0 | 22.4 | [S128] |
|  | PIM-1/UiO-66 |  |  |  |  |  | (13000^*g^) |  | (14^*g^) |  | [S51] |
|  | PIM-1/azo−UiO-66 |  |  |  |  |  | (11000^g^) |  | (19^g^) |  | [S51] |
|  | PIM-1/MOF-801 |  |  | 1752 | 362 |  | 9686 | 4.8 | 27 |  | [S52] |
|  | PIM-1/LCD |  |  |  |  | (1050^b^) | (12510^b^) |  |  | (11.9^b^) | [S54] |
|  | PIM-1/MUF-15 |  |  |  | 810 (670^a^) | 1870 | 16120 (13180^a^) |  | 19.80 (19.57^a^) | 8.60 | [S55] |
|  | PIM-1-ZIF-7−NH_2_ |  |  |  |  | 143 | 2953 (2832^e^) |  |  | 20.6 (19.8^e^) | [S58] |
|  | PIM-1/nanosized ZIF-67 |  |  |  |  |  | 2805 (2567^e^) |  |  | 21.1 (19.89^e^) | [S59] |
|  | PIM-1/FCTF-1 |  |  |  |  |  | 7300 |  |  | 16.6 | [S70] |
|  | PIM-1/PIP-Py-X PIPs |  |  |  | 99.3 (124.2^a^) | 110.5 (142.8^b^) | 6204.8 (5300^b^) |  | 62.5 (41^a^) | 56.1 (37^b^) | [S60] |
|  | PIM-1/network PIM-1 nanosheet |  |  |  |  |  | (9778^b^) |  |  | (14.4^b^) | [S62] |
|  | PIM-1/QD-FCTF-1 |  |  |  |  | 222 | 4848 (4323^*e^) |  |  | 21.8 (22.0^*e^) | [S68] |
|  | PIM-1/(PIM-1-holey GO) |  |  |  |  | (360^b^) | (4727^b^) |  |  | (13.1^b^) | [S69] |
|  | PIM-1/(PIM-1-GO) |  |  |  |  | (176^b^) | (2073^b^) |  |  | (11.8^b^) | [S67] |
|  | PIM-1/IL@MOF-1 |  |  |  | 235 |  | 9420 |  | 29 |  | [S72] |
|  | cPIM-1/PPN |  | 5503 | 2008 | 474 | 519 | 11511 | 4.2 | 24.2 | 22.2 | [S73] |
|  | PIM-1/BNNS |  |  |  |  | (644^b^) | (5940^b^) |  |  | (9.3^b^) | [S74] |
|  | PIM-1/ZIF67 |  |  |  | 1426 | 1798 | 13256 |  | 9.3 | 7.4 | [S79] |
|  | PIM-1/TSIL-ZIF-67 |  |  |  | 936 | 1219 | 12849 (8545^b^) |  | 13.7 | 10.5 (9.3^b^) | [S79] |
|  | PIM-1/ZIF-8 |  |  |  |  | 852 | 9667 |  |  | 11.35 | [S80] |
|  | PIM-1/MOF-303 |  |  |  | 257.4 (280.9^h^) |  | 6602.8 (6199.2^d^) |  | 25.6 (22.1^d^) |  | [S81] |
|  | PIM-1/BcoC-ZIF |  |  |  |  |  | (7325^a^) |  | (32.5^a^) |  | [S129] |
|  | PIM-1/S-SN |  |  |  | (392^a^) | (771^b^) | (8631^a^) (9014^b^) |  | (22^a^) | (11.8^b^) | [S84] |
|  | PIM-1/KAUST-7 |  |  |  |  |  | (5928^e^) |  |  | (23.5^e^) | [S85] |
|  | PIM-1/rHGO−TAPA |  |  |  |  | (687^b^) | (8540^b^) |  |  | (12.7^b^) | [S87] |
|  | PIM-1/GO-POSS |  | 5004^*^ |  | 559^*^ (567^a^) | 1263^*^ (1017^b^) | 11660^*^ (11682^a^) (12185^b^) |  | (20.6^a^) | (12.0^b^) | [S88] |
|  | PIM-1/sulfonated-g-C_3_N_4_ |  | 2018 | 660 | 189 | 303 | 3740 |  | 19.8 | 12.4 | [S130] |
|  | PIM-1/[SHDBU][SIm]@ZIF-67 |  |  |  |  |  | 4721 |  |  | 14.3 | [S131] |
|  | PIM-1/[SNH_2_−Pmim][STf_2_N]@ZIF-67 |  |  |  |  |  | 9536 |  |  | 31.1 | [S89] |
|  | PIM-1/NUS-8−COOH |  |  |  |  |  | (11512^b^) |  | (30.95^a^) | (17^b^) | [S132] |
|  | PIM-1/NUS-8−NH_2_ |  |  |  |  |  | (14638^b^) |  | (29.2^d^) | (9^e^) | [S90] |
|  | PIM-1/UiO-66-FA |  |  |  |  |  | (16591^a^) |  | (23.1^a^) |  | [S91] |
|  | PIM-1/PAF-1+UV |  | 4769 |  | 68 | 53 | 2081 |  | 30.6 | 39.3 | [S133] |
|  | PIM-1/UiO-66−(OH)_2_ |  | 9168 |  |  | 1958.9 | 18017 |  | 9.2 | 6.1 | [S93] |
|  | PIM-1/HZIF-8-TA |  |  |  | 201 (292^f^) | 441 (398^b^) | 8268 (6495^f^) (6352^b^) |  | 25.1 (21.9^f^) | 18.7 (16.2^b^) | [S94] |
|  | PIM-1/PDA@TD-COF |  |  |  | 369.6 |  | 9750.6 |  | 26.3 |  | [S95] |
|  | PIM-1/Ag^+^@UiO-66 |  |  |  |  |  | (14943^f^) |  | (30^f^) |  | [S98] |
|  | PIM-1/CILs@PAR |  |  | 144.7 |  |  |  | 5.0 |  |  | [S134] |
|  | PIM-1/C_2_N_x_O_1−x_ |  |  |  |  |  | 22110 (20391^f^) |  | 15.5 (17.1^f^) | 12.5 | [S135] |
|  | PIM-1/TpTta–COF |  |  |  |  |  | 9672 |  | 26.3 |  | [S99] |
|  | PIM-1/ZIF-62 |  |  |  |  | 110.8 | 5500 |  |  | 50 | [S100] |
|  | PIM-1/ZIF-62 glass |  |  |  |  | 88.7 | 5914 |  |  | 67 | [S100] |
|  | PIM-1/NH_2_−IL-UiO-66 |  |  |  |  |  | 10839 |  | 31.4 |  | [S136] |
|  | PIM-1-COOH/UiO-66−NH_2_ | 955 | 2045 | 500 | 119 | 136 | 2750 | 4.2 | 23 | 21 | [S137] |
|  | PIM-1/ (Zn-SiF_6_-py) |  |  |  |  |  | 6268 |  | 26.3 |  | [S103] |
|  | PIM-1/IL@COF |  |  |  |  |  | 9137.5 (8477.6^g^) |  | 20.2 (19.6^g^) |  | [S104] |
|  | PIM-1/MXene |  |  |  |  |  | 12475 (10800^f^) |  | 32.7 (30.5^f^) |  | [S138] |
|  | PIM-1/ZIF-8 gel |  | 6800 |  |  | 1002 |  |  |  |  | [S139] |
|  | PIM-1/HO−UiO-66-PLs |  |  |  |  |  | 7211 |  | 26 |  | [S140] |

^*^ Data based on extracted values from figures in literature. Data in parentheses indicate mixture separation data, feed gases are listed as follows: a: CO_2_/N_2_ (1:1); b: CO_2_/CH_4_ (1:1); c: CO_2_/N_2_ (4:6); d: CO_2_/N_2_ (2:8); e: CO_2_/CH_4_ (3:7); f: CO_2_/N_2_ (1:9); g: CO_2_/N_2_ (15:85).

**Table S2** Gas separation performance of thick PIM-1 membranes after aging (at 20−40 ℃ and 1−4 bar)

|  | Membranes | Time  (days) | Normalised CO_2_ permeability^*^ | Normalised CO_2_/N_2_ selectivity | Normalised CO_2_/CH_4_ selectivity | Refs. |
| --- | --- | --- | --- | --- | --- | --- |
| Unmodified PIM-1 | PIM-1 | 10 | 0.59 (CH_4_) | - | - | [S43] |
|  | PIM-1 | 24 | 0.3 |  |  | [S122] |
|  | PIM-1 | 30 | 0.6 | 1.57 |  | [S51] |
|  | PIM-1 | 30 | 0.47 (CH_4_) | - | - | [S7] |
|  | PIM-1 | 30 | 0.61 |  |  | [S28] |
|  | PIM-1 | 30 | 0.76 |  |  | [S132] |
|  | PIM-1 | 35 | 0.73 | 0.83 | 0.87 | [S55] |
|  | PIM-1 | 40 | 0.67 |  | 1.10 | [S80] |
|  | PIM-1 | 45 | 0.79 |  | 1.08 | [S79] |
|  | PIM-1 | 50 | 0.53 |  |  | [S70] |
|  | PIM-1 | 60 | 0.22 |  |  | [S68] |
|  | PIM-1 | 60 | 0.61 |  |  | [S47] |
|  | PIM-1 | 63 | 0.19 |  |  | [S94] |
|  | PIM-1 | 70 | 0.53 | 0.53 |  | [S129] |
|  | PIM-1 | 80 | 0.14 |  |  | [S125] |
|  | PIM-1 | 90 | 0.65 |  |  | [S128] |
|  | PIM-1 | 90 | 0.46 |  |  | [S30] |
|  | PIM-1 | 100 | 0.4 |  |  | [S52] |
|  | PIM-1 | 100 | 0.42 |  |  | [S72] |
|  | PIM-1 | 100 | 0.39 | 1.19 |  | [S104] |
|  | PIM-1 | 105 | 0.43 | 1.28 | 1.32 | [S82] |
|  | PIM-1 | 120 | 0.3 |  |  | [S121] |
|  | PIM-1 | 120 | 0.62 |  | 1.58 | [S35] |
|  | PIM-1 | 120 | 0.50 |  |  | [S58] |
|  | PIM-1 | 120 | 0.28 |  |  | [S136] |
|  | PIM-1 | 120 | 0.48 |  |  | [S59] |
|  | PIM-1 | 120 | 0.80 | 1.75 | 1.7 | [S32] |
|  | PIM-1 | 120 | 0.58 |  |  | [S48] |
|  | PIM-1 | 150 | 0.51 |  |  | [S67] |
|  | PIM-1 | 150 | 0.53 |  | 1.33 | [S69] |
|  | PIM-1 | 150 | 0.37 |  |  | [S84] |
|  | PIM-1 | 150 | 0.34 |  |  | [S85] |
|  | PIM-1 | 150 | 0.34 |  |  | [S123] |
|  | PIM-1 | 155 | 0.31 |  | 1.47 | [S37] |
|  | PIM-1 | 160 | 0.44 |  |  | [S88] |
|  | PIM-1 | 160 | 0.31 |  | 1.54 | [S54] |
|  | PIM-1 | 160 | 0.26 |  |  | [S91] |
|  | PIM-1 | 180 | 0.49 |  |  | [S60] |
|  | PIM-1 | 180 | 0.32 | 1.22 | 1.53 | [S73] |
|  | PIM-1 | 180 | 0.23 | 1.07 | 1.11 | [S141] |
|  | PIM-1 | 200 | 0.41 |  |  | [S20] |
|  | PIM-1 | 210 | 0.45 |  |  | [S127] |
|  | PIM-1 | 240 | 0.48 | 1.27 | - | [S13] |
|  | PIM-1 | 310 | 0.36 | - | - | [S12] |
|  | PIM-1 | 360 | 0.27 |  | 1.34 | [S87] |
|  | PIM-1 | 381 | 0.44 |  |  | [S71] |
|  | PIM-1 | 400 | 0.17 |  | 1.2 | [S41] |
|  | PIM-1 | 414 | 0.42 |  | 1.2 | [S74] |
|  | PIM-1 | 414 | 0.42 |  | 1.2 | [S74] |
|  | PIM-1 | 455 | 0.39 | 1.80 | 1.52 | [S111] |
|  | PIM-1 | 600 | 0.28 |  |  | [S130] |
|  | PIM-1 | 1000 | 0.5 |  |  | [S50] |
| Chain modification | PIM-1 120℃, 81 min | 44 | 0.74 |  |  | [S108] |
|  | PIM-1−SO_3_H | 60 | 0.26 |  | 1.64 | [S75] |
|  | PIM-1/Py | 87 | 0.41 |  |  | [S97] |
|  | PIM-1/MePy | 87 | 0.45 |  |  | [S97] |
|  | PIM-1 160℃, 33 min | 96 | 1.47 |  |  | [S108] |
|  | PIM-C1 | 180 | 0.39 | 0.84 | 0.91 | [S141] |
|  | PIM-1−NH_2_ | 448 | 0.45 |  |  | [S71] |
| Post-modification | Thermally crosslinked PIM-1 | 10 | 0.81 (CH_4_) | - | - | [S43] |
|  | PIM-UV 30 min | 30 | 0.85 (CH_4_) | - | - | [S7] |
|  | PIM-1-O_3_ | 30 | 0.81 | 0.95 | 0.85 | [S86] |
|  | Amine-PIM-1 | 42 | 0.88 |  |  | [S39] |
|  | f-PIM-1 | 60 | 0.98 | 1.21 | 1.31 | [S77] |
|  | NA-PIM-1 | 60 | 0.5 | 0.99 |  | [S92] |
|  | NA-PIM-1-Zn-PEI | 60 | 0.59 | 1.08 |  | [S92] |
|  | Aminated PIM-1 | 100 | 0.63 | 0.91 |  | [S102] |
|  | SCPIMs | 100 | 0.35 | 1.38 | 1.71 | [S56] |
|  | PIM-1−deBOC(acid) | 180 | 0.3 | 1.85 | 3.25 | [S64] |
|  | PIM-1−deBOC(thermal) | 180 | 0.35 | 1.05 | 1.62 | [S64] |
|  | PIM-1−deBOC | 180 | 0.4 | 1.25 | 1.6 | [S64] |
|  | PIM-1−NH_2_ | 180 | 0.6 | 1.7 | 2.7 | [S64] |
|  | PIM-1−*t*BOC | 180 | 0.8 | 0.9 | 0.99 | [S64] |
|  | f-PIM-1 | 300 | 0.75 | 0.87 | 0.46 | [S113] |
|  | TOX-PIM-1 | 455 | 0.28 | 1.03 | 1.1 | [S111] |
| Blends | PIM-1/MEEP80 | 60 | 0.65 | 0.9 |  | [S44] |
| MMMs | PIM-1/TpTta–COF | 15 | 0.99 | 0.95 |  | [S99] |
|  | PIM-1/PDA@TD-COF | 15 | 0.83 | 1.02 |  | [S95] |
|  | PIM-1/azo-UiO-66 | 30 | 0.73 | 1.36 |  | [S51] |
|  | PIM-1/NUS-8−COOH | 30 | 0.89 | 0.85 |  | [S132] |
|  | PIM-1/PEG-POSS | 30 | 0.77 |  |  | [S28] |
|  | PIM-1-COOH/UiO-66−NH_2_ | 31 | 0.41 | 1.14 | 1.37 | [S137] |
|  | PIM-1/MUF-15 | 35 | 0.64 | 1.28 | 1.45 | [S55] |
|  | PIM-1/ZIF-8 | 40 | 0.41 |  | 1.22 | [S80] |
|  | PIM-1/TSIL-ZIF-67 | 45 | 0.86 |  | 1.09 | [S79] |
|  | PIM-1/ZIF67 | 45 | 0.75 |  | 1.03 | [S79] |
|  | PIM-1/FCTF-1 | 50 | 0.58 |  |  | [S70] |
|  | PIM-1/QD-FCTF-1 | 60 | 0.39 |  |  | [S68] |
|  | PIM-1/PAF-1+UV | 60 | 0.78 |  |  | [S133] |
|  | PIM-1/DMBA-Silica | 60 | 0.9 | 1.03 |  | [S47] |
|  | PIM-1/HZIF-8-TA | 63 | 0.23 |  |  | [S94] |
|  | PIM-1/Ti-UiO-66 | 63 | 0.3 |  |  | [S122] |
|  | PIM-1/BcoC-ZIF | 70 | 0.7 | 1.12 |  | [S129] |
|  | PAO-PIM-1/UiO-66−NH_2_ | 80 | 0.69 |  |  | [S125] |
|  | PIM-1/OH-pDCX | 90 | 0.75 |  |  | [S128] |
|  | PIM-1/UiO-66 | 90 | 0.52 |  | 1.52 | [S27] |
|  | PIM-1/OAPS | 90 | 0.70 |  |  | [S30] |
|  | PIM-1/HO-UiO-66-PLs | 100 | 0.74 | 1.26 |  | [S140] |
|  | PIM-1/IL@MOF-1 | 100 | 0.74 | 0.97 |  | [S72] |
|  | PIM-1/IL@COF | 100 | 0.66 |  |  | [S104] |
|  | PIM-1/MOF-801 | 100 | 0.70 | 1.07 |  | [S52] |
|  | PIM-1/TiO_2_ | 105 | 0.55 | 1.24 | 1.26 | [S82] |
|  | PIM-1/PDASS | 120 | 0.77 |  |  | [S48] |
|  | PIM-1/POSS | 120 | 0.4 |  |  | [S121] |
|  | PIM-1/nanosized ZIF-67 | 120 | 0.63 |  |  | [S59] |
|  | PIM-1/NUS-8 nanosheet | 120 | 0.77 |  | 1.19 | [S35] |
|  | PIM-1/ β-CD | 120 | 0.94 | 1.05 | 1.2 | [S32] |
|  | PIM-1/NH_2_-IL-UiO-66 | 120 | 0.81 | 1.25 |  | [S136] |
|  | PIM-1-ZIF-7−NH_2_ | 120 | 0.74 |  |  | [S58] |
|  | PIM-1/S-SN | 150 | 0.68 |  | 1.35 | [S84] |
|  | PIM-1/KAUST-7 | 150 | 0.91 |  | 1.17 | [S85] |
|  | PIM-1/(PIM-1-holey GO) | 150 | 0.7 |  | 1.08 | [S69] |
|  | PIM-1/(PIM-1-GO) | 150 | 0.85 |  |  | [S67] |
|  | PIM-1/HCP | 150 | 0.60 |  |  | [S123] |
|  | PIM-1/rGO-OA^a^ | 155 | 0.61 |  | 1.32 | [S37] |
|  | PIM-1/UiO-66-FA | 160 | 0.75 | 1.27 |  | [S91] |
|  | PIM-1/GO-POSS | 160 | 0.74 |  |  | [S88] |
|  | PIM-1/LCD | 160 | 0.71 |  | 1.50 | [S54] |
|  | PIM-1/Al_2_O_3_ | 180 | 0.46 | 0.68 | 1.08 | [S76] |
|  | cPIM-1/PPN | 180 | 0.86 | 1.12 | 0.97 | [S73] |
|  | PIM-1/PIP-Py-AC PIPs | 180 | 0.66 |  |  | [S60] |
|  | PIM-1-g-C_3_N_4_ | 200 | 0.33 |  |  | [S20] |
|  | PIM-1/TPFC−CH_2_NH_2_ | 210 | 0.78 |  |  | [S127] |
|  | PIM-1/network PIM-1 nanosheet | 210 | 0.20 |  | 1.84* | [S62] |
|  | PIM-1/PAF-1 | 240 | 0.93 | 1.62 | - | [S13] |
|  | PIM-1-COOH | 330 | 0.26 |  | 1.21 | [S57] |
|  | PIM-1/UiO-66−NH_2_ | 350 | 0.52 | 1.05 |  | [S27] |
|  | PIM-1/*in-situ* crosslinked UiO-66-NH_2_ | 400 | 0.76 |  | 1.03 | [S41] |
|  | PIM-1/CC3 | 400 | 0.34 | 1.36 | - | [S12] |
|  | PIM-1/BNNS | 414 | 0.78 |  | 1.3 | [S74] |
|  | PIM-1/sulfinated-g-C_3_N_4_ | 600 | 0.25 | 1.04 | 1.21 | [S130] |
|  | PIM-1/rHGO-TAPA | 611 | 0.45 |  | 1.09 | [S87] |
|  | PIM-1/MIL-101 | 2555 | 0.11 | 1.76 |  | [S50] |

^*^Normalisation was conducted using the values from the same references for fresh membranes at day 0.

**Table S3** Gas separation performance of PIM-1 TFC/TFN membranes after aging (at 20−40 ℃ and 1−4 bar)

| Membranes | Aging time  (days) | Normalised CO_2_ permeance | Normalised CO_2_/N_2_ selectivity | Normalised CO_2_/CH_4_ selectivity | Refs. |
| --- | --- | --- | --- | --- | --- |
| PIM-1/CuBDC NS | 4 | 1.01 |  |  | [S142] |
| PIM-1 | 28 | 0.03 |  | 0.97 | [S84] |
| PIM-1/SN | 28 | 0.05 |  | 0.90 | [S84] |
| PIM-1/S-SN | 28 | 0.14 |  | 1.18 | [S84] |
| PIM-1, #3b, network content 7.8 wt.% | 28 | 0.08 | 0.71 |  | [S108] |
| PIM-1. #1, network content 0.8 wt.% | 28 | 0.03 | 0.32 |  | [S109] |
| PIM-1. #3, network content 85.3 wt.% | 28 | 0.91 | 0.43 |  | [S109] |
| Blended 20%PIM-1#3 in PIM-1#1 | 28 | 0.59 | 0.90 |  | [S109] |
| PIM-1 | 28 | 0.29 | 0.73 | 0.93 | [S143] |
| PIM-1/OAPS | 30 | 0.14 |  |  | [S30] |
| PIM-1/OAPS | 30 | 0.02 |  |  | [S30] |
| PIM-1 | 30 | 0.11 |  |  | [S30] |
| PIM-1 | 30 | 0.01 |  |  | [S30] |
| PIM-1 | 56 | 0.11 | 1.63 |  | [S144] |
| PIM-1/Ni-MOF-74 | 56 | 0.24 | 0.97 |  | [S144] |
| PIM-1/UiO-66−NH_2_ | 56 | 0.12 | 1 |  | [S144] |
| B-cPIM-1 | 60 | 1.56 | 1.72 | 1.47 | [S145] |
| B-PIM-1 | 60 | 0.35 | 1.05 | 1.09 | [S145] |
| D-PIM-1 | 60 | 0.15 | 1.64 | 2.53 | [S145] |
| D-cPIM-1 | 60 | 0.48 | 0.71 | 0.76 | [S145] |
| PIM-1 | 63 | 0.46 |  |  | [S94] |
| PIM-1/ZIF-8 | 63 | 0.27 |  |  | [S94] |
| PIM-1/HZIF-8-TA | 63 | 0.14 |  |  | [S94] |
| PIM-1/C-UiO-66−NH_2_/cPIM-1 | 63 | 0.94 | 1.16 | 1.08 | [S143] |
| PIM-1 | 90 | 0.12 | 1.73 |  | [S146] |
| PIM-1/HCP | 90 | 0.32 | 1.52 |  | [S146] |
| PIM-1/C-HCP | 90 | 0.35 | 1.71 |  | [S146] |
| PIM-1/PTMSP | 98 | 0.03 | 0.97 |  | [S147] |
| PIM-1 | 360 | 0.27 |  |  | [S87] |
| PIM-1/rHGO-TAPA | 365 | 0.81 |  |  | [S87] |
| PIM-1 | 360 | 0.27 |  |  | [S87] |

**Supplementary References**

[S1] P. M. Budd, K. J. Msayib, C. E. Tattershall, B. S. Ghanem, K. J. Reynolds et al., Gas separation membranes from polymers of intrinsic microporosity. J. Membr. Sci. **251**, 263–269 (2005). <https://doi.org/10.1016/j.memsci.2005.01.009>

[S2] P. M. Budd, N. B. McKeown, B. S. Ghanem, K. J. Msayib, D. Fritsch et al., Gas permeation parameters and other physicochemical properties of a polymer of intrinsic microporosity: Polybenzodioxane PIM-1. J. Membr. Sci. **325**, 851–860 (2008). <https://doi.org/10.1016/j.memsci.2008.09.010>

[S3] N. Du, G. P. Robertson, J. Song, I. Pinnau, S. Thomas et al., Polymers of intrinsic microporosity containing trifluoromethyl and phenylsulfone groups as materials for membrane gas separation. Macromolecules **41**, 9656–9662 (2008). <https://doi.org/10.1021/ma801858d>

[S4] C. L. Staiger, S. J. Pas, A. J. Hill, C. J. Cornelius, Gas separation, free volume distribution, and physical aging of a highly microporous spirobisindane polymer. Chem. Mater. **20**, 2606–2608 (2008). <https://doi.org/10.1021/cm071722t>

[S5] S. Thomas, I. Pinnau, N. Du, M. D. Guiver, Pure- and mixed-gas permeation properties of a microporous spirobisindane-based ladder polymer (PIM-1). J. Membr. Sci. **333**, 125–131 (2009). <https://doi.org/10.1016/j.memsci.2009.02.003>

[S6] N. Du, M. M. Dal-Cin, G. P. Robertson, M. D. Guiver, Decarboxylation-induced cross-linking of polymers of intrinsic microporosity (PIMs) for membrane gas separation. Macromolecules **45**, 5134–5139 (2012). <https://doi.org/10.1021/ma300751s>

[S7] F. Y. Li, Y. Xiao, Y. K. Ong, T.-S. Chung, UV-rearranged PIM-1 polymeric membranes for advanced hydrogen purification and production. Adv. Energy Mater. **2**, 1456–1466 (2012). <https://doi.org/10.1002/aenm.201200296>

[S8] I.-D. Carja, S. R. Tavares, O. Shekhah, A. Ozcan, R. Semino et al., Insights into the enhancement of MOF/polymer adhesion in mixed-matrix membranes via polymer functionalization. ACS Appl. Mater. Interfaces **13**, 29041–29047 (2021). <https://doi.org/10.1021/acsami.1c03859>

[S9] Q. Song, S. Cao, P. Zavala-Rivera, L. Ping Lu, W. Li et al., Photo-oxidative enhancement of polymeric molecular sieve membranes. Nat. Commun. **4**, 1918 (2013). <https://doi.org/10.1038/ncomms2942>

[S10] N. Du, G. P. Robertson, M. M. Dal-Cin, L. Scoles, M. D. Guiver, Polymers of intrinsic microporosity (PIMs) substituted with methyl tetrazole. Polymer **53**, 4367–4372 (2012). <https://doi.org/10.1016/j.polymer.2012.07.055>

[S11] M. M. Khan, V. Filiz, G. Bengtson, S. Shishatskiy, M. M. Rahman et al., Enhanced gas permeability by fabricating mixed matrix membranes of functionalized multiwalled carbon nanotubes and polymers of intrinsic microporosity (PIM). J. Membr. Sci. **436**, 109–120 (2013). <https://doi.org/10.1016/j.memsci.2013.02.032>

[S12] A. F. Bushell, P. M. Budd, M. P. Attfield, J. T. A. Jones, T. Hasell et al., Nanoporous organic polymer/cage composite membranes. Angew. Chem. Int. Ed. **52**, 1253–1256 (2013). <https://doi.org/10.1002/anie.201206339>

[S13] C. H. Lau, P. T. Nguyen, M. R. Hill, A. W. Thornton, K. Konstas et al., Ending aging in super glassy polymer membranes. Angew. Chem. Int. Ed. **53**, 5322–5326 (2014). <https://doi.org/10.1002/anie.201402234>

[S14] R. Swaidan, B. S. Ghanem, E. Litwiller, I. Pinnau, Pure- and mixed-gas CO₂/CH₄ separation properties of PIM-1 and an amidoxime-functionalized PIM-1. J. Membr. Sci. **457**, 95–102 (2014). <https://doi.org/10.1016/j.memsci.2014.01.055>

[S15] X. Mei Wu, Q. Gen Zhang, P. Ju Lin, Y. Qu, A. Mei Zhu et al., Towards enhanced CO₂ selectivity of the PIM-1 membrane by blending with polyethylene glycol. J. Membr. Sci. **493**, 147–155 (2015). <https://doi.org/10.1016/j.memsci.2015.05.077>

[S16] L. Hao, K.-S. Liao, T.-S. Chung, Photo-oxidative PIM-1 based mixed matrix membranes with superior gas separation performance. J. Mater. Chem. A **3**, 17273–17281 (2015). <https://doi.org/10.1039/C5TA03776J>

[S17] N. Tien-Binh, H. Vinh-Thang, X. Y. Chen, D. Rodrigue, S. Kaliaguine, Crosslinked MOF-polymer to enhance gas separation of mixed matrix membranes. J. Membr. Sci. **520**, 941–950 (2016). <https://doi.org/10.1016/j.memsci.2016.08.045>

[S18] W. F. Yong, F. Y. Li, Y. C. Xiao, P. Li, K. P. Pramoda et al., Molecular engineering of PIM-1/Matrimid blend membranes for gas separation. J. Membr. Sci. **407**, 47–57 (2012). <https://doi.org/10.1016/j.memsci.2012.03.038>

[S19] H. Zhao, Q. Xie, X. Ding, J. Chen, M. Hua et al., High performance post-modified polymers of intrinsic microporosity (PIM-1) membranes based on multivalent metal ions for gas separation. J. Membr. Sci. **514**, 305–312 (2016). <https://doi.org/10.1016/j.memsci.2016.05.013>

[S20] Z. Tian, S. Wang, Y. Wang, X. Ma, K. Cao et al., Enhanced gas separation performance of mixed matrix membranes from graphitic carbon nitride nanosheets and polymers of intrinsic microporosity. J. Membr. Sci. **514**, 15–24 (2016). <https://doi.org/10.1016/j.memsci.2016.04.019>

[S21] W. F. Yong, Z. K. Lee, T.-S. Chung, M. Weber, C. Staudt et al., Blends of a polymer of intrinsic microporosity and partially sulfonated polyphenylenesulfone for gas separation. ChemSusChem **9**, 1953–1962 (2016). <https://doi.org/10.1002/cssc.201600354>

[S22] J. Zhang, H. Kang, J. Martin, S. Zhang, S. Thomas et al., The enhancement of chain rigidity and gas transport performance of polymers of intrinsic microporosity via intramolecular locking of the spiro-carbon. Chem. Commun. **52**, 6553–6556 (2016). <https://doi.org/10.1039/C6CC02308H>

[S23] J. Ma, Y. Ying, X. Guo, H. Huang, D. Liu et al., Fabrication of mixed-matrix membrane containing metal–organic framework composite with task-specific ionic liquid for efficient CO₂ separation. J. Mater. Chem. A **4**, 7281–7288 (2016). <https://doi.org/10.1039/C6TA02611G>

[S24] A. K. Sekizkardes, V. A. Kusuma, G. Dahe, E. A. Roth, L. J. Hill et al., Separation of carbon dioxide from flue gas by mixed matrix membranes using dual phase microporous polymeric constituents. Chem. Commun. **52**, 11768–11771 (2016). <https://doi.org/10.1039/C6CC04811K>

[S25] J. W. Jeon, D.-G. Kim, E.-h. Sohn, Y. Yoo, Y. S. Kim et al., Highly carboxylate-functionalized polymers of intrinsic microporosity for CO₂-selective polymer membranes. Macromolecules **50**, 8019–8027 (2017). <https://doi.org/10.1021/acs.macromol.7b01332>

[S26] X. Wu, Z. Tian, S. Wang, D. Peng, L. Yang et al., Mixed matrix membranes comprising polymers of intrinsic microporosity and covalent organic framework for gas separation. J. Membr. Sci. **528**, 273–283 (2017). <https://doi.org/10.1016/j.memsci.2017.01.042>

[S27] M. R. Khdhayyer, E. Esposito, A. Fuoco, M. Monteleone, L. Giorno et al., Mixed matrix membranes based on UiO-66 MOFs in the polymer of intrinsic microporosity PIM-1. Sep. Purif. Technol. **173**, 304–313 (2017). <https://doi.org/10.1016/j.seppur.2016.09.036>

[S28] L. Yang, Z. Tian, X. Zhang, X. Wu, Y. Wu et al., Enhanced CO₂ selectivities by incorporating CO₂-philic PEG-POSS into polymers of intrinsic microporosity membrane. J. Membr. Sci. **543**, 69–78 (2017). <https://doi.org/10.1016/j.memsci.2017.08.050>

[S29] K. Halder, M. M. Khan, J. Grünauer, S. Shishatskiy, C. Abetz et al., Blend membranes of ionic liquid and polymers of intrinsic microporosity with improved gas separation characteristics. J. Membr. Sci. **539**, 368–382 (2017). <https://doi.org/10.1016/j.memsci.2017.06.022>

[S30] Y. Kinoshita, K. Wakimoto, A. H. Gibbons, A. P. Isfahani, H. Kusuda et al., Enhanced PIM-1 membrane gas separation selectivity through efficient dispersion of functionalized POSS fillers. J. Membr. Sci. **539**, 178–186 (2017). <https://doi.org/10.1016/j.memsci.2017.05.072>

[S31] B. Ghalei, K. Sakurai, Y. Kinoshita, K. Wakimoto, Ali P. Isfahani et al., Enhanced selectivity in mixed matrix membranes for CO₂ capture through efficient dispersion of amine-functionalized MOF nanoparticles. Nat. Energy **2**, 17086 (2017). <https://doi.org/10.1038/nenergy.2017.86>

[S32] J. Liu, Y. Xiao, K.-S. Liao, T.-S. Chung, Highly permeable and aging resistant 3D architecture from polymers of intrinsic microporosity incorporated with beta-cyclodextrin. J. Membr. Sci. **523**, 92–102 (2017). <https://doi.org/10.1016/j.memsci.2016.10.001>

[S33] S. Zhao, J. Liao, D. Li, X. Wang, N. Li, Blending of compatible polymer of intrinsic microporosity (PIM-1) with Tröger's base polymer for gas separation membranes. J. Membr. Sci. **566**, 77–86 (2018). <https://doi.org/10.1016/j.memsci.2018.08.010>

[S34] N. Du, G. P. Robertson, J. Song, I. Pinnau, M. D. Guiver, High-performance carboxylated polymers of intrinsic microporosity (PIMs) with tunable gas transport properties. Macromolecules **42**, 6038–6043 (2009). <https://doi.org/10.1021/ma9009017>

[S35] Y. Cheng, S. R. Tavares, C. M. Doherty, Y. Ying, E. Sarnello et al., Enhanced polymer crystallinity in mixed-matrix membranes induced by metal–organic framework nanosheets for efficient CO₂ capture. ACS Appl. Mater. Interfaces **10**, 43095–43103 (2018). <https://doi.org/10.1021/acsami.8b16386>

[S36] N. Prasetya, B. P. Ladewig, New azo-DMOF-1 MOF as a photoresponsive low-energy CO₂ adsorbent and its exceptional CO₂/N₂ separation performance in mixed matrix membranes. ACS Appl. Mater. Interfaces **10**, 34291–34301 (2018). <https://doi.org/10.1021/acsami.8b12261>

[S37] M. Alberto, R. Bhavsar, J. M. Luque-Alled, A. Vijayaraghavan, P. M. Budd et al., Impeded physical aging in PIM-1 membranes containing graphene-like fillers. J. Membr. Sci. **563**, 513–520 (2018). <https://doi.org/10.1016/j.memsci.2018.06.026>

[S38] M. Chen, F. Soyekwo, Q. Zhang, C. Hu, A. Zhu et al., Graphene oxide nanosheets to improve permeability and selectivity of PIM-1 membrane for carbon dioxide separation. J. Ind. Eng. Chem. **63**, 296–302 (2018). <https://doi.org/10.1016/j.jiec.2018.02.030>

[S39] B. Satilmis, M. Lanč, A. Fuoco, C. Rizzuto, E. Tocci et al., Temperature and pressure dependence of gas permeation in amine-modified PIM-1. J. Membr. Sci. **555**, 483–496 (2018). <https://doi.org/10.1016/j.memsci.2018.03.039>

[S40] X. Wu, W. Liu, H. Wu, X. Zong, L. Yang et al., Nanoporous ZIF-67 embedded polymers of intrinsic microporosity membranes with enhanced gas separation performance. J. Membr. Sci. **548**, 309–318 (2018). <https://doi.org/10.1016/j.memsci.2017.11.038>

[S41] N. Tien-Binh, D. Rodrigue, S. Kaliaguine, In-situ cross-interface linking of PIM-1 polymer and UiO-66-NH₂ for outstanding gas separation and physical aging control. J. Membr. Sci. **548**, 429–438 (2018). <https://doi.org/10.1016/j.memsci.2017.11.054>

[S42] M. M. Khan, S. Shishatskiy, V. Filiz, Mixed matrix membranes of boron icosahedron and polymers of intrinsic microporosity (PIM-1) for gas separation. Membranes **8**, (2018). <https://doi.org/10.3390/membranes8010001>

[S43] F. Y. Li, Y. Xiao, T.-S. Chung, S. Kawi, High-performance thermally self-cross-linked polymer of intrinsic microporosity (PIM-1) membranes for energy development. Macromolecules **45**, 1427–1437 (2012). <https://doi.org/10.1021/ma202667y>

[S44] A. K. Sekizkardes, V. A. Kusuma, J. S. McNally, D. W. Gidley, K. Resnik et al., Microporous polymeric composite membranes with advanced film properties: Pore intercalation yields excellent CO₂ separation performance. J. Mater. Chem. A **6**, 22472–22477 (2018). <https://doi.org/10.1039/C8TA07424K>

[S45] G. Yu, Y. Li, Z. Wang, T. X. Liu, G. Zhu et al., Mixed matrix membranes derived from nanoscale porous organic frameworks for permeable and selective CO₂ separation. J. Membr. Sci. **591**, 117343 (2019). <https://doi.org/10.1016/j.memsci.2019.117343>

[S46] J. Sánchez-Laínez, A. Pardillos-Ruiz, M. Carta, R. Malpass-Evans, N. B. McKeown et al., Polymer engineering by blending PIM-1 and 6FDA-DAM for ZIF-8 containing mixed matrix membranes applied to CO₂ separations. Sep. Purif. Technol. **224**, 456–462 (2019). <https://doi.org/10.1016/j.seppur.2019.05.035>

[S47] N. Sakaguchi, M. Tanaka, M. Yamato, H. Kawakami, Superhigh CO₂-permeable mixed matrix membranes composed of a polymer of intrinsic microporosity (PIM-1) and surface-modified silica nanoparticles. ACS Appl. Polym. Mater. **1**, 2516–2524 (2019). <https://doi.org/10.1021/acsapm.9b00624>

[S48] G. Dong, J. Zhang, Z. Wang, J. Wang, P. Zhao et al., Interfacial property modulation of PIM-1 through polydopamine-derived submicrospheres for enhanced CO₂/N₂ separation performance. ACS Appl. Mater. Interfaces **11**, 19613–19622 (2019). <https://doi.org/10.1021/acsami.9b02281>

[S49] G. Yu, X. Zou, L. Sun, B. Liu, Z. Wang et al., Constructing connected paths between UiO-66 and PIM-1 to improve membrane CO₂ separation with crystal-like gas selectivity. Adv. Mater. **31**, 1806853 (2019). <https://doi.org/10.1002/adma.201806853>

[S50] M. Khdhayyer, A. F. Bushell, P. M. Budd, M. P. Attfield, D. Jiang et al., Mixed matrix membranes based on MIL-101 metal–organic frameworks in polymer of intrinsic microporosity PIM-1. Sep. Purif. Technol. **212**, 545–554 (2019). <https://doi.org/10.1016/j.seppur.2018.11.055>

[S51] N. Prasetya, B. P. Ladewig, An insight into the effect of azobenzene functionalities studied in UiO-66 frameworks for low-energy CO₂ capture and CO₂/N₂ membrane separation. J. Mater. Chem. A **7**, 15164–15172 (2019). <https://doi.org/10.1039/C9TA02096A>

[S52] W. Chen, Z. Zhang, L. Hou, C. Yang, H. Shen et al., Metal-organic framework MOF-801/PIM-1 mixed-matrix membranes for enhanced CO₂/N₂ separation performance. Sep. Purif. Technol. **250**, 117198 (2020). <https://doi.org/10.1016/j.seppur.2020.117198>

[S53] M. D. Guiver, M. Yahia, M. M. Dal-Cin, G. P. Robertson, S. Saeedi Garakani et al., Gas transport in a polymer of intrinsic microporosity (PIM-1) substituted with pseudo-ionic liquid tetrazole-type structures. Macromolecules **53**, 8951–8959 (2020). <https://doi.org/10.1021/acs.macromol.0c01321>

[S54] M. Tamaddondar, A. B. Foster, M. Carta, P. Gorgojo, N. B. McKeown et al., Mitigation of physical aging with mixed matrix membranes based on cross-linked PIM-1 fillers and PIM-1. ACS Appl. Mater. Interfaces **12**, 46756–46766 (2020). <https://doi.org/10.1021/acsami.0c13838>

[S55] H. Yin, A. Alkaş, Y. Zhang, Y. Zhang, S. G. Telfer, Mixed matrix membranes (MMMs) using an emerging metal-organic framework (MUF-15) for CO₂ separation. J. Membr. Sci. **609**, 118245 (2020). <https://doi.org/10.1016/j.memsci.2020.118245>

[S56] S. Zhou, Y. Sun, B. Xue, S. Li, J. Zheng et al., Controlled superacid-catalyzed self-cross-linked polymer of intrinsic microporosity for high-performance CO₂ separation. Macromolecules **53**, 7988–7996 (2020). <https://doi.org/10.1021/acs.macromol.0c01590>

[S57] K. Mizrahi Rodriguez, A. X. Wu, Q. Qian, G. Han, S. Lin et al., Facile and time-efficient carboxylic acid functionalization of PIM-1: Effect on molecular packing and gas separation performance. Macromolecules **53**, 6220–6234 (2020). <https://doi.org/10.1021/acs.macromol.0c00933>

[S58] Y. Wang, Y. Ren, H. Wu, X. Wu, H. Yang et al., Amino-functionalized ZIF-7 embedded polymers of intrinsic microporosity membrane with enhanced selectivity for biogas upgrading. J. Membr. Sci. **602**, 117970 (2020). <https://doi.org/10.1016/j.memsci.2020.117970>

[S59] C. Ye, X. Wu, H. Wu, L. Yang, Y. Ren et al., Incorporating nano-sized ZIF-67 to enhance selectivity of polymers of intrinsic microporosity membranes for biogas upgrading. Chem. Eng. Sci. **216**, 115497 (2020). <https://doi.org/10.1016/j.ces.2020.115497>

[S60] C. Wang, F. Guo, H. Li, J. Xu, J. Hu et al., A porous ionic polymer bionic carrier in a mixed matrix membrane for facilitating selective CO₂ permeability. J. Membr. Sci. **598**, 117677 (2020). <https://doi.org/10.1016/j.memsci.2019.117677>

[S61] Z. Wang, Q. Shen, J. Liang, Y. Zhang, J. Jin, Adamantane-grafted polymer of intrinsic microporosity with finely tuned interchain spacing for improved CO₂ separation performance. Sep. Purif. Technol. **233**, 116008 (2020). <https://doi.org/10.1016/j.seppur.2019.116008>

[S62] M. Tamaddondar, A. B. Foster, J. M. Luque-Alled, K. J. Msayib, M. Carta et al., Intrinsically microporous polymer nanosheets for high-performance gas separation membranes. Macromol. Rapid Commun. **41**, 1900572 (2020). <https://doi.org/10.1002/marc.201900572>

[S63] X. Chen, Z. Zhang, L. Wu, X. Liu, S. Xu et al., Polymers of intrinsic microporosity having bulky substitutes and cross-linking for gas separation membranes. ACS Appl. Polym. Mater. **2**, 987–995 (2020). <https://doi.org/10.1021/acsapm.9b01193>

[S64] K. Mizrahi Rodriguez, S. Lin, A. X. Wu, G. Han, J. J. Teesdale et al., Leveraging free volume manipulation to improve the membrane separation performance of amine-functionalized PIM-1. Angew. Chem. Int. Ed. **60**, 6593–6599 (2021). <https://doi.org/10.1002/anie.202012441>

[S65] A. K. Sekizkardes, S. Budhathoki, L. Zhu, V. Kusuma, Z. Tong et al., Molecular design and fabrication of PIM-1/polyphosphazene blend membranes with high performance for CO₂/N₂ separation. J. Membr. Sci. **640**, 119764 (2021). <https://doi.org/10.1016/j.memsci.2021.119764>

[S66] W. Ji, K. Li, Y.-G. Min, W. Shi, J. Li et al., Remarkably enhanced gas separation properties of PIM-1 at sub-ambient temperatures. J. Membr. Sci. **623**, 119091 (2021). <https://doi.org/10.1016/j.memsci.2021.119091>

[S67] J. M. Luque-Alled, A. W. Ameen, M. Alberto, M. Tamaddondar, A. B. Foster et al., Gas separation performance of MMMs containing (PIM-1)-functionalized GO derivatives. J. Membr. Sci. **623**, 118902 (2021). <https://doi.org/10.1016/j.memsci.2020.118902>

[S68] H. Jiang, Z. Guo, H. Wang, X. Liu, Y. Ren et al., Solvent-processable 0D covalent organic framework quantum dot engineered composite membranes for biogas upgrading. J. Membr. Sci. **640**, 119803 (2021). <https://doi.org/10.1016/j.memsci.2021.119803>

[S69] J. M. Luque-Alled, M. Tamaddondar, A. B. Foster, P. M. Budd, P. Gorgojo, PIM-1/holey graphene oxide mixed matrix membranes for gas separation: Unveiling the role of holes. ACS Appl. Mater. Interfaces **13**, 55517–55533 (2021). <https://doi.org/10.1021/acsami.1c15640>

[S70] H. Jiang, J. Zhang, T. Huang, J. Xue, Y. Ren et al., Mixed-matrix membranes with covalent triazine framework fillers in polymers of intrinsic microporosity for CO₂ separations. Ind. Eng. Chem. Res. **59**, 5296–5306 (2020). <https://doi.org/10.1021/acs.iecr.9b04632>

[S71] K. Mizrahi Rodriguez, F. M. Benedetti, N. Roy, A. X. Wu, Z. P. Smith, Sorption-enhanced mixed-gas transport in amine functionalized polymers of intrinsic microporosity (PIMs). J. Mater. Chem. A **9**, 23631–23642 (2021). <https://doi.org/10.1039/D1TA06530K>

[S72] W. Chen, Z. Zhang, C. Yang, J. Liu, H. Shen et al., PIM-based mixed-matrix membranes containing MOF-801/ionic liquid nanocomposites for enhanced CO₂ separation performance. J. Membr. Sci. **636**, 119581 (2021). <https://doi.org/10.1016/j.memsci.2021.119581>

[S73] W. Han, C. Zhang, M. Zhao, F. Yang, Y. Yang et al., Post-modification of PIM-1 and simultaneously in situ synthesis of porous polymer networks into PIM-1 matrix to enhance CO₂ separation performance. J. Membr. Sci. **636**, 119544 (2021). <https://doi.org/10.1016/j.memsci.2021.119544>

[S74] A. W. Ameen, J. Ji, M. Tamaddondar, S. Moshenpour, A. B. Foster et al., 2D boron nitride nanosheets in PIM-1 membranes for CO₂/CH₄ separation. J. Membr. Sci. **636**, 119527 (2021). <https://doi.org/10.1016/j.memsci.2021.119527>

[S75] H. Dong, Z. Zhu, K. Li, Q. Li, W. Ji et al., Significantly improved gas separation properties of sulfonated PIM-1 by direct sulfonation using SO₃ solution. J. Membr. Sci. **635**, 119440 (2021). <https://doi.org/10.1016/j.memsci.2021.119440>

[S76] X. Chen, L. Wu, H. Yang, Y. Qin, X. Ma et al., Tailoring the microporosity of polymers of intrinsic microporosity for advanced gas separation by atomic layer deposition. Angew. Chem. Int. Ed. **60**, 17875–17880 (2021). <https://doi.org/10.1002/anie.202016901>

[S77] X. Ma, K. Li, Z. Zhu, H. Dong, J. Lv et al., High-performance polymer molecular sieve membranes prepared by direct fluorination for efficient helium enrichment. J. Mater. Chem. A **9**, 18313–18322 (2021). <https://doi.org/10.1039/D1TA04099E>

[S78] Z. Gao, Y. Wang, H. Wu, Y. Ren, Z. Guo et al., Surface functionalization of polymers of intrinsic microporosity (PIMs) membrane by polyphenol for efficient CO₂ separation. Green Chem. Eng. **2**, 70–76 (2021). <https://doi.org/10.1016/j.gce.2020.12.003>

[S79] J. Han, L. Bai, H. Jiang, S. Zeng, B. Yang et al., Task-specific ionic liquids tuning ZIF-67/PIM-1 mixed matrix membranes for efficient CO₂ separation. Ind. Eng. Chem. Res. **60**, 593–603 (2021). <https://doi.org/10.1021/acs.iecr.0c04830>

[S80] M. Yahia, Q. N. Phan Le, N. Ismail, M. Essalhi, O. Sundman et al., Effect of incorporating different ZIF-8 crystal sizes in the polymer of intrinsic microporosity, PIM-1, for CO₂/CH₄ separation. Micropor. Mesopor. Mater. **312**, 110761 (2021). <https://doi.org/10.1016/j.micromeso.2020.110761>

[S81] Q. Shen, S. Cong, J. Zhu, Y. Zhang, R. He et al., Novel pyrazole-based MOF synergistic polymer of intrinsic microporosity membranes for high-efficient CO₂ capture. J. Membr. Sci. **664**, 121107 (2022). <https://doi.org/10.1016/j.memsci.2022.121107>

[S82] X. Niu, G. Dong, D. Li, Y. Zhang, Y. Zhang, Atomic layer deposition modified PIM-1 membranes for improved CO₂ separation: A comparative study on the microstructure-performance relationships. J. Membr. Sci. **664**, 121103 (2022). <https://doi.org/10.1016/j.memsci.2022.121103>

[S83] G. Ferraro, C. Astorino, M. Bartoli, A. Martis, S. Lettieri et al., Ionic liquids-polymer of intrinsic microporosity (PIMs) blend membranes for CO₂ separation. Membranes **12**, (2022). <https://doi.org/10.3390/membranes12121262>

[S84] S. Mohsenpour, Z. Guo, F. Almansour, S. M. Holmes, P. M. Budd et al., Porous silica nanosheets in PIM-1 membranes for CO₂ separation. J. Membr. Sci. **661**, 120889 (2022). <https://doi.org/10.1016/j.memsci.2022.120889>

[S85] K. Chen, L. Ni, H. Zhang, C. Xiao, L. Li et al., Incorporating KAUST-7 into PIM-1 towards mixed matrix membranes with long-term stable CO₂/CH₄ separation performance. J. Membr. Sci. **661**, 120848 (2022). <https://doi.org/10.1016/j.memsci.2022.120848>

[S86] W. Ji, H. Geng, Z. Chen, H. Dong, H. Matsuyama et al., Facile tailoring molecular sieving effect of PIM-1 by in-situ O₃ treatment for high performance hydrogen separation. J. Membr. Sci. **662**, 120971 (2022). <https://doi.org/10.1016/j.memsci.2022.120971>

[S87] F. Almansour, M. Alberto, A. B. Foster, S. Mohsenpour, P. M. Budd et al., Thin film nanocomposite membranes of superglassy PIM-1 and amine-functionalised 2D fillers for gas separation. J. Mater. Chem. A **10**, 23341–23351 (2022). <https://doi.org/10.1039/D2TA06339E>

[S88] S. Mohsenpour, A. W. Ameen, S. Leaper, C. Skuse, F. Almansour et al., PIM-1 membranes containing POSS - graphene oxide for CO₂ separation. Sep. Purif. Technol. **121447**, (2022). <https://doi.org/10.1016/j.seppur.2022.121447>

[S89] Z. Zhang, X. Cao, C. Geng, Y. Sun, Y. He et al., Machine learning aided high-throughput prediction of ionic liquid@MOF composites for membrane-based CO₂ capture. J. Membr. Sci. **650**, 120399 (2022). <https://doi.org/10.1016/j.memsci.2022.120399>

[S90] Y. Pu, Z. Yang, V. Wee, Z. Wu, Z. Jiang et al., Amino-functionalized NUS-8 nanosheets as fillers in PIM-1 mixed matrix membranes for CO₂ separations. J. Membr. Sci. **641**, 119912 (2022). <https://doi.org/10.1016/j.memsci.2021.119912>

[S91] C. Geng, Y. Sun, Z. Zhang, Z. Qiao, C. Zhong, Mitigated aging in a defective metal–organic framework pillared polymer of an intrinsic porosity hybrid membrane for efficient gas separation. ACS Sustainable Chem. Eng. **10**, 3643–3650 (2022). <https://doi.org/10.1021/acssuschemeng.1c08485>

[S92] J. Guan, X. Wang, J. Du, Q. Liang, W. He et al., Surface-engineered PIM-1 membranes for facile CO₂ capture. Chem. Eng. J. **477**, 147017 (2023). <https://doi.org/10.1016/j.cej.2023.147017>

[S93] Y. Sun, F. Fan, L. Bai, T. Li, J. Guan et al., Hydrogen-bonded hybrid membranes based on hydroxylated metal-organic frameworks and PIM-1 for ultrafast hydrogen separation. Results Eng. **20**, 101398 (2023). <https://doi.org/10.1016/j.rineng.2023.101398>

[S94] B. Zhu, S. He, Y. Yang, S. Li, C. H. Lau et al., Boosting membrane carbon capture via multifaceted polyphenol-mediated soldering. Nat. Commun. **14**, 1697 (2023). <https://doi.org/10.1038/s41467-023-37479-9>

[S95] X. Chang, H. Guo, Q. Chang, Z. Tian, Y. Zhang et al., Mixed-matrix membranes composed of dopamine modified covalent organic framework and PIM-1 for efficient CO₂/N₂ separation. J. Membr. Sci. **686**, 122017 (2023). <https://doi.org/10.1016/j.memsci.2023.122017>

[S96] H. Amir, R. Tamime, Z. Shamair, A. L. Khan, H. AlMohamadi et al., Enhanced gas separation performance of PIM-1 blend membranes incorporating ionic liquid (3-(trimethoxysilyl) propan-1-aminium acetate ([SAPTMS][SAc])) as filler: Investigation of morphology, compatibility and transport properties. Fuel **349**, 128669 (2023). <https://doi.org/10.1016/j.fuel.2023.128669>

[S97] S. Kang, G. Huo, Z. Zhang, T. Guo, Z. Dai et al., Polymers of intrinsic porosity with bulky side groups on the spirobisindane moieties for gas separation. ACS Appl. Polym. Mater. **5**, 8660–8669 (2023). <https://doi.org/10.1021/acsapm.3c01762>

[S98] Z. Lin, Z. Yuan, K. Wang, X. He, Synergistic tuning mixed matrix membranes by Ag⁺-doping in UIO-66-NH₂/polymers of intrinsic microporosity for remarkable CO₂/N₂ separation. J. Membr. Sci. **681**, 121775 (2023). <https://doi.org/10.1016/j.memsci.2023.121775>

[S99] G. Dai, Q. Zhang, S. Xiong, L. Deng, Z. Gao et al., Building interfacial compatible PIM-1-based mixed-matrix membranes with β-ketoenamine-linked COF fillers for effective CO₂/N₂ separation. J. Membr. Sci. **676**, 121561 (2023). <https://doi.org/10.1016/j.memsci.2023.121561>

[S100] Y. Feng, W. Yan, Z. Kang, X. Zou, W. Fan et al., Thermal treatment optimization of porous MOF glass and polymer for improving gas permeability and selectivity of mixed matrix membranes. Chem. Eng. J. **465**, 142873 (2023). <https://doi.org/10.1016/j.cej.2023.142873>

[S101] Y. Sun, J. Zhang, H. Li, F. Fan, Q. Zhao et al., Ester-crosslinked polymers of intrinsic microporosity membranes with enhanced plasticization resistance for CO₂ separation. Sep. Purif. Technol. **314**, 123623 (2023). <https://doi.org/10.1016/j.seppur.2023.123623>

[S102] L. Chen, P. Su, J. Liu, S. Chen, J. Huang et al., Post-synthesis amination of polymer of intrinsic microporosity membranes for CO₂ separation. AIChE J. **69**, e18050 (2023). <https://doi.org/10.1002/aic.18050>

[S103] Z. Liu, S. Cong, J. Zhang, G. Dong, Y. Zhang, Mixed matrix membrane with amorphous metal-based complexes displays high CO₂ separation performance. Sep. Purif. Technol. **330**, 125349 (2024). <https://doi.org/10.1016/j.seppur.2023.125349>

[S104] Q. Chang, H. Guo, Z. Shang, C. Zhang, Y. Zhang et al., PIM-based mixed matrix membranes containing covalent organic frameworks/ionic liquid composite materials for effective CO₂/N₂ separation. Sep. Purif. Technol. **330**, 125518 (2024). <https://doi.org/10.1016/j.seppur.2023.125518>

[S105] N. Du, H. B. Park, G. P. Robertson, M. M. Dal-Cin, T. Visser et al., Polymer nanosieve membranes for CO₂-capture applications. Nat. Mater. **10**, 372–375 (2011). <https://doi.org/10.1038/nmat2989>

[S106] C. R. Mason, L. Maynard-Atem, N. M. Al-Harbi, P. M. Budd, P. Bernardo et al., Polymer of intrinsic microporosity incorporating thioamide functionality: Preparation and gas transport properties. Macromolecules **44**, 6471–6479 (2011). <https://doi.org/10.1021/ma200918h>

[S107] K. Halder, S. Neumann, G. Bengtson, M. M. Khan, V. Filiz et al., Polymers of intrinsic microporosity postmodified by vinyl groups for membrane applications. Macromolecules **51**, 7309–7319 (2018). <https://doi.org/10.1021/acs.macromol.8b01252>

[S108] A. B. Foster, M. Tamaddondar, J. M. Luque-Alled, W. J. Harrison, Z. Li et al., Understanding the topology of the polymer of intrinsic microporosity PIM-1: Cyclics, tadpoles, and network structures and their impact on membrane performance. Macromolecules **53**, 569–583 (2020). <https://doi.org/10.1021/acs.macromol.9b02185>

[S109] A. B. Foster, J. L. Beal, M. Tamaddondar, J. M. Luque-Alled, B. Robertson et al., Importance of small loops within PIM-1 topology on gas separation selectivity in thin film composite membranes. J. Mater. Chem. A **9**, 21807–21823 (2021). <https://doi.org/10.1039/D1TA03712A>

[S110] N. Du, M. M. Dal-Cin, I. Pinnau, A. Nicalek, G. P. Robertson et al., Azide-based cross-linking of polymers of intrinsic microporosity (PIMs) for condensable gas separation. Macromol. Rapid Commun. **32**, 631–636 (2011). <https://doi.org/10.1002/marc.201000775>

[S111] Q. Song, S. Cao, R. H. Pritchard, B. Ghalei, S. A. Al-Muhtaseb et al., Controlled thermal oxidative crosslinking of polymers of intrinsic microporosity towards tunable molecular sieve membranes. Nat. Commun. **5**, 4813 (2014). <https://doi.org/10.1038/ncomms5813>

[S112] S. He, X. Jiang, S. Li, F. Ran, J. Long et al., Intermediate thermal manipulation of polymers of intrinsic microporous (PIMs) membranes for gas separations. AIChE J. **66**, e16543 (2020). <https://doi.org/10.1002/aic.16543>

[S113] N. A. Belov, A. Y. Alentiev, R. Y. Nikiforov, S. V. Chirkov, D. A. Bezgin et al., Gas separation properties of PIM-1 films treated by elemental fluorine in liquid perfluorodecalin. Polymer **280**, 126033 (2023). <https://doi.org/10.1016/j.polymer.2023.126033>

[S114] W. F. Yong, F. Y. Li, T.-S. Chung, Y. W. Tong, Highly permeable chemically modified PIM-1/Matrimid membranes for green hydrogen purification. J. Mater. Chem. A **1**, 13914–13925 (2013). <https://doi.org/10.1039/C3TA13308G>

[S115] W. F. Yong, F. Y. Li, T. S. Chung, Y. W. Tong, Molecular interaction, gas transport properties and plasticization behavior of cPIM-1/Torlon blend membranes. J. Membr. Sci. **462**, 119–130 (2014). <https://doi.org/10.1016/j.memsci.2014.03.046>

[S116] W. F. Yong, T.-S. Chung, Miscible blends of carboxylated polymers of intrinsic microporosity (cPIM-1) and Matrimid. Polymer **59**, 290–297 (2015). <https://doi.org/10.1016/j.polymer.2015.01.013>

[S117] G. Bengtson, S. Neumann, V. Filiz, Membranes of polymers of intrinsic microporosity (PIM-1) modified by poly(ethylene glycol). Membranes **7**, (2017). <https://doi.org/10.3390/membranes7020028>

[S118] J. Ahn, W.-J. Chung, I. Pinnau, J. Song, N. Du et al., Gas transport behavior of mixed-matrix membranes composed of silica nanoparticles in a polymer of intrinsic microporosity (PIM-1). J. Membr. Sci. **346**, 280–287 (2010). <https://doi.org/10.1016/j.memsci.2009.09.047>

[S119] C. R. Mason, M. G. Buonomenna, G. Golemme, P. M. Budd, F. Galiano et al., New organophilic mixed matrix membranes derived from a polymer of intrinsic microporosity and silicalite-1. Polymer **54**, 2222–2230 (2013). <https://doi.org/10.1016/j.polymer.2013.02.032>

[S120] A. F. Bushell, M. P. Attfield, C. R. Mason, P. M. Budd, Y. Yampolski et al., Gas permeation parameters of mixed matrix membranes based on the polymer of intrinsic microporosity PIM-1 and the zeolitic imidazolate framework ZIF-8. J. Membr. Sci. **427**, 48–62 (2013). <https://doi.org/10.1016/j.memsci.2012.09.035>

[S121] W. F. Yong, K. H. A. Kwek, K.-S. Liao, T.-S. Chung, Suppression of aging and plasticization in highly permeable polymers. Polymer **77**, 377–386 (2015). <https://doi.org/10.1016/j.polymer.2015.09.075>

[S122] S. J. D. Smith, B. P. Ladewig, A. J. Hill, C. H. Lau, M. R. Hill, Post-synthetic Ti-exchanged UiO-66 metal-organic frameworks that deliver exceptional gas permeability in mixed matrix membranes. Sci. Rep. **5**, 7823 (2015). <https://doi.org/10.1038/srep07823>

[S123] T. Mitra, R. S. Bhavsar, D. J. Adams, P. M. Budd, A. I. Cooper, PIM-1 mixed matrix membranes for gas separations using cost-effective hypercrosslinked nanoparticle fillers. Chem. Commun. **52**, 5581–5584 (2016). <https://doi.org/10.1039/C6CC00261G>

[S124] N. Konnertz, Y. Ding, W. J. Harrison, P. M. Budd, A. Schönhals et al., Molecular mobility and gas transport properties of nanocomposites based on PIM-1 and polyhedral oligomeric phenethyl-silsesquioxanes (POSS). J. Membr. Sci. **529**, 274–285 (2017). <https://doi.org/10.1016/j.memsci.2017.02.007>

[S125] Z. Wang, H. Ren, S. Zhang, F. Zhang, J. Jin, Polymers of intrinsic microporosity/metal–organic framework hybrid membranes with improved interfacial interaction for high-performance CO₂ separation. J. Mater. Chem. A **5**, 10968–10977 (2017). <https://doi.org/10.1039/C7TA01773A>

[S126] C. G. Morris, N. M. Jacques, H. G. W. Godfrey, T. Mitra, D. Fritsch et al., Stepwise observation and quantification and mixed matrix membrane separation of CO₂ within a hydroxy-decorated porous host. Chem. Sci. **8**, 3239–3248 (2017). <https://doi.org/10.1039/C6SC04343G>

[S127] C. Wang, F. Guo, H. Li, J. Xu, J. Hu et al., Porous organic polymer as fillers for fabrication of defect-free PIM-1-based mixed matrix membranes with facilitating CO₂-transfer chain. J. Membr. Sci. **564**, 115–122 (2018). <https://doi.org/10.1016/j.memsci.2018.07.018>

[S128] R. Hou, S. J. D. Smith, C. D. Wood, R. J. Mulder, C. H. Lau et al., Solvation effects on the permeation and aging performance of PIM-1-based MMMs for gas separation. ACS Appl. Mater. Interfaces **11**, 6502–6511 (2019). <https://doi.org/10.1021/acsami.8b19207>

[S129] Y. Sun, C. Geng, Z. Zhang, Z. Qiao, C. Zhong, Two-dimensional basic cobalt carbonate supported ZIF-67 composites towards mixed matrix membranes for efficient CO₂/N₂ separation. J. Membr. Sci. **661**, 120928 (2022). <https://doi.org/10.1016/j.memsci.2022.120928>

[S130] B. K. Voon, H. Shen Lau, C. Z. Liang, W. F. Yong, Functionalized two-dimensional g-C₃N₄ nanosheets in PIM-1 mixed matrix membranes for gas separation. Sep. Purif. Technol. **296**, 121354 (2022). <https://doi.org/10.1016/j.seppur.2022.121354>

[S131] J. Han, H. Jiang, S. Zeng, Y. Bai, X. Zhang et al., CO₂ separation performance for PIM-based mixed matrix membranes embedded by superbase ionic liquids. J. Mol. Liq. **359**, 119375 (2022). <https://doi.org/10.1016/j.molliq.2022.119375>

[S132] D. Wang, Y. Ying, Y. Zheng, Y. Pu, Z. Yang et al., Induced polymer crystallinity in mixed matrix membranes by metal-organic framework nanosheets for gas separation. J. Membr. Sci. Lett. **2**, 100017 (2022). <https://doi.org/10.1016/j.memlet.2022.100017>

[S133] R. Hou, S. J. D. Smith, K. Konstas, C. M. Doherty, C. D. Easton et al., Synergistically improved PIM-1 membrane gas separation performance by PAF-1 incorporation and UV irradiation. J. Mater. Chem. A **10**, 10107–10119 (2022). <https://doi.org/10.1039/D2TA00138A>

[S134] H. Zhao, T. Song, X. Ding, R. Cai, X. Tan et al., PIM-1 mixed matrix membranes incorporated with magnetic responsive cobalt-based ionic liquid for O₂/N₂ separation. J. Membr. Sci. **679**, 121713 (2023). <https://doi.org/10.1016/j.memsci.2023.121713>

[S135] Z. Tian, D. Li, W. Zheng, Q. Chang, Y. Sang et al., Heteroatom-doped noble carbon-tailored mixed matrix membranes with ultrapermeability for efficient CO₂ separation. Mater. Horiz. **10**, 3660–3667 (2023). <https://doi.org/10.1039/D3MH00463E>

[S136] C. Geng, Y. Sun, Z. Zhang, Z. Qiao, C. Zhong, Mixed matrix metal-organic framework membranes for efficient CO₂/N₂ separation under humid conditions. AIChE J. **69**, e18025 (2023). <https://doi.org/10.1002/aic.18025>

[S137] W.-N. Wu, K. Mizrahi Rodriguez, N. Roy, J. J. Teesdale, G. Han et al., Engineering the polymer–MOF interface in microporous composites to address complex mixture separations. ACS Appl. Mater. Interfaces **15**, 52893–52907 (2023). <https://doi.org/10.1021/acsami.3c11300>

[S138] K. Wang, D. Chen, J. Tang, Z. Hong, Z. Zhu et al., PIM-1-based membranes mediated with CO₂-philic MXene nanosheets for superior CO₂/N₂ separation. Chem. Eng. J. **483**, 149305 (2024). <https://doi.org/10.1016/j.cej.2024.149305>

[S139] K. Zhang, X. Luo, S. Li, X. Tian, Q. Wang et al., ZIF-8 gel/PIM-1 mixed matrix membranes for enhanced H₂/CH₄ separations. Chem. Eng. J. **484**, 149489 (2024). <https://doi.org/10.1016/j.cej.2024.149489>

[S140] F. Wang, Z. Wang, J. Yu, S. Han, X. Li et al., Mixed matrix membranes with intrinsic microporous/UiO-66 post-synthesis modifications with no defects for efficient CO₂/N₂ separation. Sep. Purif. Technol. **333**, 125892 (2024). <https://doi.org/10.1016/j.seppur.2023.125892>

[S141] M. Liu, X. Lu, M. D. Nothling, C. M. Doherty, L. Zu et al., Physical aging investigations of a spirobisindane-locked polymer of intrinsic microporosity. ACS Materials Lett. **2**, 993–998 (2020). <https://doi.org/10.1021/acsmaterialslett.0c00184>

[S142] Y. Cheng, X. Wang, C. Jia, Y. Wang, L. Zhai et al., Ultrathin mixed matrix membranes containing two-dimensional metal-organic framework nanosheets for efficient CO₂/CH₄ separation. J. Membr. Sci. **539**, 213–223 (2017). <https://doi.org/10.1016/j.memsci.2017.06.011>

[S143] B. Qiu, M. Yu, J. M. Luque-Alled, S. Ding, A. B. Foster et al., High gas permeability in aged superglassy membranes with nanosized UiO-66-NH₂/CPIM-1 network fillers. Angew. Chem. Int. Ed. **63**, e202316356 (2024). <https://doi.org/10.1002/anie.202316356>

[S144] M. Liu, M. D. Nothling, P. A. Webley, J. Jin, Q. Fu et al., High-throughput CO₂ capture using PIM-1@MOF-based thin film composite membranes. Chem. Eng. J. **396**, 125328 (2020). <https://doi.org/10.1016/j.cej.2020.125328>

[S145] M. Yu, A. B. Foster, M. Alshurafa, J. M. Luque-Alled, P. Gorgojo et al., CO₂ separation using thin film composite membranes of acid-hydrolyzed PIM-1. J. Membr. Sci. **679**, 121697 (2023). <https://doi.org/10.1016/j.memsci.2023.121697>

[S146] R. S. Bhavsar, T. Mitra, D. J. Adams, A. I. Cooper, P. M. Budd, Ultrahigh-permeance PIM-1-based thin film nanocomposite membranes on PAN supports for CO₂ separation. J. Membr. Sci. **564**, 878–886 (2018). <https://doi.org/10.1016/j.memsci.2018.07.089>

[S147] I. Borisov, D. Bakhtin, J. M. Luque-Alled, A. Rybakova, V. Makarova et al., Synergistic enhancement of gas selectivity in thin film composite membranes of PIM-1. J. Mater. Chem. A **7**, 6417–6430 (2019). <https://doi.org/10.1039/C8TA10691F>
